# Effect of TiO_2_ on Selected Pathogenic and Opportunistic Intestinal Bacteria

**DOI:** 10.1007/s12011-021-02843-7

**Published:** 2021-07-23

**Authors:** Ewa Baranowska-Wójcik, Dominik Szwajgier, Klaudia Gustaw

**Affiliations:** Department of Biotechnology, Microbiology and Human Nutrition, University of Life Sciences, Skromna Street 8, 20-704 Lublin, Poland

**Keywords:** TiO_2_ NPs, Nanoparticles, E171, Bacterial, Microbiota

## Abstract

Food-grade titanium dioxide (TiO_2_) containing a nanoparticle fraction (TiO_2_ NPs-nanoparticles) is widely used as a food additive (E171 in the EU). In recent years, questions concerning its effect on the gastrointestinal microbiota have been raised. In the present study, we examined interactions between bacteria and TiO_2_. The study involved six pathogenic/opportunistic bacterial strains and four different-sized TiO_2_ types: three types of food-grade E171 compounds and TiO_2_ NPs (21 nm). Each bacterial strain was exposed to four concentrations of TiO_2_ (60, 150, 300, and 600 mg/L TiO_2_). The differences in the growth of the analyzed strains, caused by the type and concentration of TiO_2_, were observed. The growth of a majority of the strains was shown to be inhibited after exposure to 300 and 600 mg/L of the food-grade E171 and TiO_2_ NPs.

## Introduction

Titanium dioxide (TiO_2_) is an oxide of white metal used as a food additive (E171) due to its whitening and brightening properties. Since it contains nano-fractions [1, 2], the additive raises increasing concerns about the risk of disruption of the intestinal barrier and dysbiosis of the intestinal microflora [3, 4]. For example, France was the first country to prohibit the use of this food additive for fear of its negative effects on the human organism [5].

Besides commensal bacteria, the gastrointestinal microbiome is regularly exposed to food-borne (transient) bacteria, which may also come into contact with TiO_2_ via ingestion or passage of food through the intestine [6, 7]. This may exert an effect on the resident microbiome and, consequently, on human health. Investigation of the interactions between bacteria and TiO_2_ can provide considerable amounts of valuable information. There are only few studies assessing the interactions of NPs with intestinal microflora and their effect on host health. Most research is focused on direct interactions with intestinal epithelial cells [8, 9] and a vast majority of studies concerned modified TiO_2_.

In the present study, we focused on the effect of unmodified TiO_2_ NPs applied in food production on selected pathogenic and opportunistic intestinal bacteria (Table 1). The aim of the experiment was to reproduce, as accurately as possible, the probable conditions (i.e., bacterial and TiO_2_ concentrations) in which intestinal bacteria come into contact with this food additive.

**Table 1:** List of bacterial strains under study

Species and strain
1. *Escherichia coli* DH5α
2. *Bacillus subtilis* PCM 486
3. *Micrococcus luteus* DSM 20,030
4. *Salmonella anatum* ATCC 9270
5. *Salmonella enterica* ATCC 10,708
6. *Listeria monocytogenes* ATCC 35,160

## Material and Methods

### Nanoparticles

Four types of TiO_2_ were used in the study. Food-grade TiO_2_ (E171) was purchased from three Polish suppliers: Warchem Sp z o.o., Marki; Biomus, Lublin; and Food Colors, Piotrków Trybunalski (nos. 1, 2, and 3, respectively). For comparison purposes, TiO_2_ NPs were purchased from Sigma-Aldrich (CAS Number: 718467-100G. Titanium (IV) oxide, nanopowder, 21 nm) (no. 4). The characteristics of the studied TiO_2_ are presented in our earlier work [10].

### Sample Preparation

Aqueous suspensions of each TiO_2_ type were prepared daily, before each experiment, in deionized water (60, 150, 300, and 600 mg/L; a, b, c, and d, respectively). Next, each sample was subjected to 30-min sonication in an ultrasonic bath (25 °C, 250 W, 50 Hz).

### Bacterial Cultures

Pure MRS media, as well as media with the addition of nanoparticles at four concentrations (a, b, c, d), were prepared. The growth of bacteria (Table 1) was controlled for 72 h by measurements of the optical density (OD) at 600 nm every 2 h using Bioscreen C (Labsystem, Helsinki, Finland) as in Gustaw et al. [11]. Control variants were performed in each experiment (samples lacking bacteria or NPs). The comparison of these values showed the inhibitory properties of the particular types of nanoparticles and their concentrations. On the basis of the results obtained, growth kinetics values were determined using the PYTHON script for individual strains and each medium variant used (Supplementary materials, Fig. 2S1-2S6) [12].

## Results

**Fig. 1:**
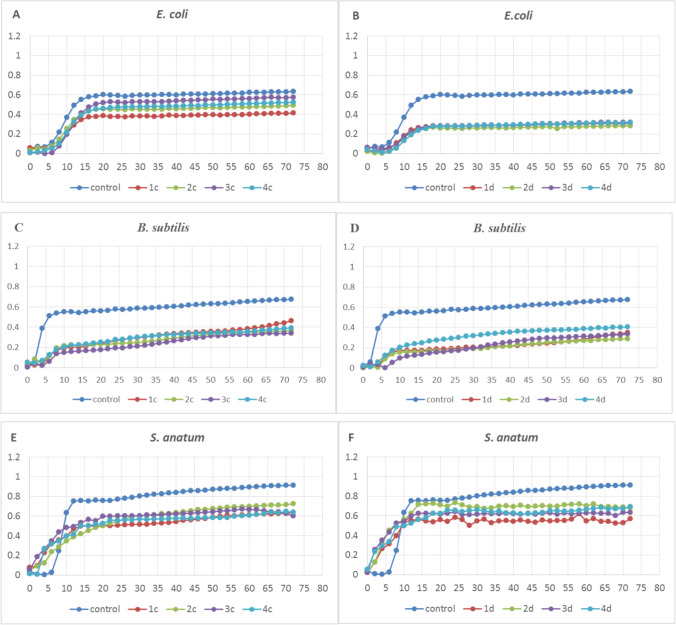
Growth of selected bacteria after application of four types of TiO_2_ at the concentration of 300 (**A**, **C**, **E**), 600 (**B**, **D**, **F**) mg/L; E171 (nos. 1, 2, 3), TiO_2_ NPs (no. 4)

**Fig. 2:**
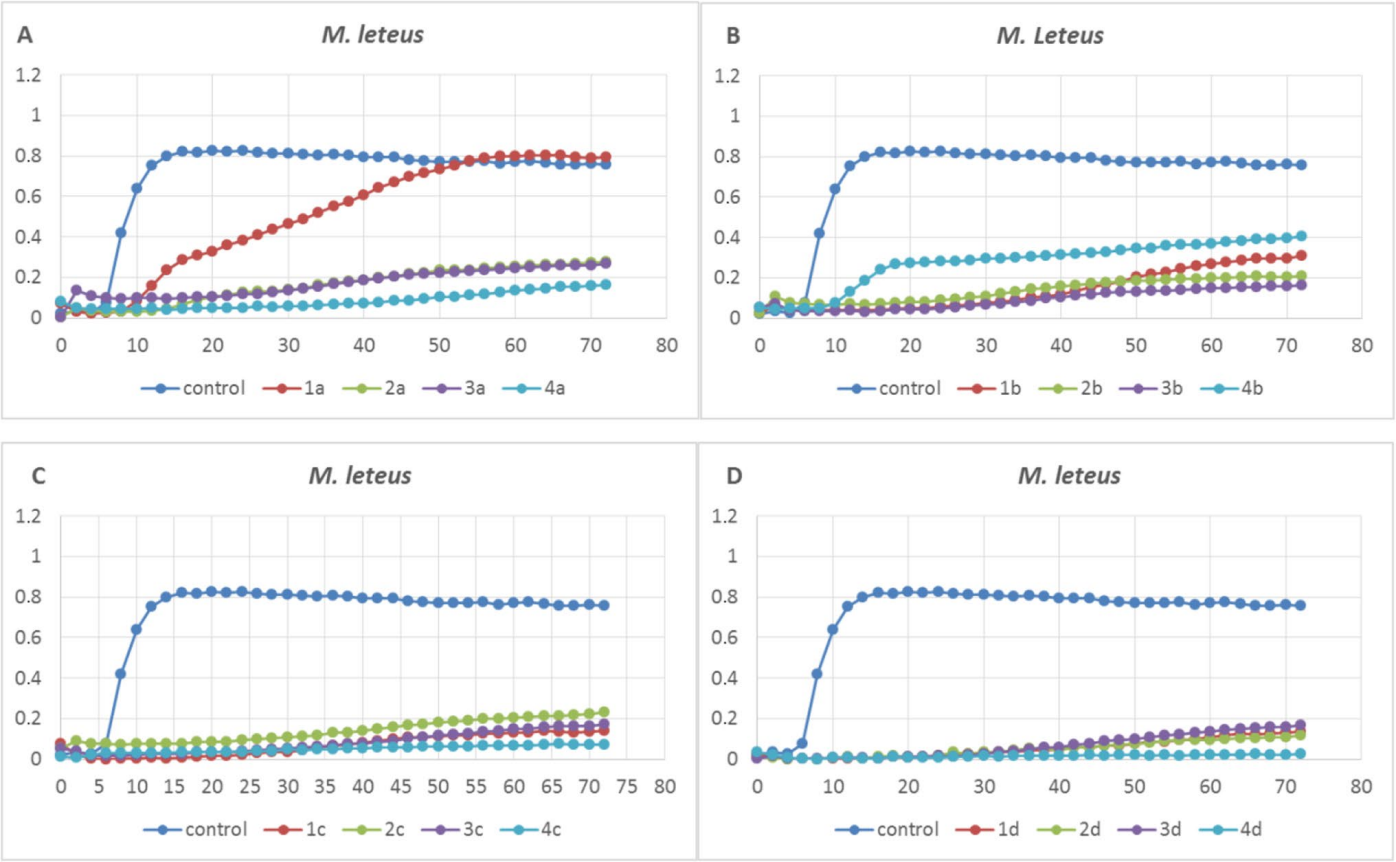
Growth of selected bacteria (*M. luteus*) after application of four types of TiO_2_ at the concentrations of 60 (**A**), 150 (**B**), 300 (**C**), and 600 (**D**) mg/L; E171 (nos. 1, 2, 3), TiO_2_ NPs (no. 4)

The monitoring of the bacterial growth for 72 h revealed the differences in the inhibition of the strains, depending on the type and concentration of TiO_2_ (Figs. 1, 2; Supplementary materials, Fig. 1S1). The percentage inhibition was directly proportional to the increasing concentration of the nanoparticles, in comparison with the control.

There were differences in the growth of *E. coli*, *B. subtilis*, and *S. anatum* on the Luria–Bertani medium supplemented with the four different types of TiO_2_ at the concentration of 300 mg/L (Fig. 1A, C, E) and relatively high differences at the exposure to the highest concentration of 600 mg/L (Fig. 1B, D, F). *Escherichia coli* turned out to be the most resistant of these three species to the negative effects of the NPs, as no growth inhibition was detected at the concentrations of 30 and 150 mg/L, and the max OD value at the concentration of 600 mg/L decreased by half (Table 2). This was also visible in the growth kinetics of this strain, as the duration of the lag phase differed by only 1 h at the highest concentration. In turn, *B. subtilis* proved to be almost as susceptible to growth inhibition as *M. luteus*. Evident growth inhibition was noted at the lowest concentration; however, this species adapted to the unfavorable environment over time and exhibited a similar growth rate as that in the control.

**Table 2:** Bacterial growth parameters (600 mg/L); E 171 (nos. 1, 2, 3), TiO_2_ NPs (no. 4)

species	Types of TiO_2_ NPs	Lag time (hours)	Max specific growth rate (hours^−1^)	Doubling time (hours)	Max OD	Min OD	R2
*M. luteus*	Control	6.67	0.16	4.38	0.83	0.02	1.00
	1	n.g	n.g	n.g	0.14	0.00	n.g
	2	n.g	n.g	n.g	0.12	0.00	n.g
	3	n.g	n.g	n.g	0.17	0.00	n.g
	4	n.g	n.g	n.g	0.04	0.00	n.g
*E. coli*	Control	6.77	0.07	9.38	0.64	0.05	1.00
	1	6.56	0.04	19.45	0.31	0.02	0.99
	2	7.20	0.04	17.41	0.28	0.00	0.99
	3	7.32	0.03	23.35	0.32	0.04	0.98
	4	7.91	0.03	21.49	0.32	0.01	0.98
*B. subtilis*	Control	6.77	0.07	9.38	0.64	0.05	1.00
	1	n.g	n.g	n.g	0.19	0.02	n.g
	2	n.g	n.g	n.g	0.21	0.00	n.g
	3	n.g	n.g	n.g	0.19	0.00	n.g
	4	n.g	n.g	n.g	0.17	0.00	n.g
*S. anatum*	Control	7.82	0.19	3.57	0.91	0.00	0.97
	1	0.00	0.06	11.63	0.62	0.02	0.98
	2	0.00	0.07	9.65	0.74	0.03	0.99
	3	0.00	0.07	9.66	0.65	0.02	0.98
	4	0.00	0.04	15.45	0.70	0.05	0.96

*n.g.*, no growth

The *S. enterica* and *L. monocytogenes* strains did not substantially inhibit the growth of bacteria after the application of all types and concentrations of TiO_2_. The slightest differences in the case of all other strains were noted after the application of the lowest concentrations (60 and 150 mg/L) (Fig. 1S1). The growth curve for the bacteria cultured on the medium supplemented with these two concentrations of all types of nanoparticles (1, 2, 3, 4) was similar to that in the control. An exception was the *M. luteus* strain, whose growth was inhibited already at the lowest concentration (60 mg/L) (Fig. 2A), and significant growth inhibition was noted after the exposure to nanoparticle 1 (Fig. 2). This strain exhibited substantial growth reduction in all experimental variants, and the increasing concentration was accompanied by an increasing percentage of growth inhibition, relative to the control, until the growth was suppressed completely at the highest concentration.

## Discussion

This study demonstrated that the four different types of TiO_2_ applied at the concentrations of 300 mg/L and 600 mg/L inhibited the growth of four bacterial strains. Interestingly, at the 300 mg/L concentration, there were evident differences in the bacterial responses to the different E171/NPs TiO_2_ forms used in the experiment (Figs. 1 and 2). As demonstrated in our work as well as by other authors in the past, both the type and concentration of TiO_2_ may have an influence on the outcome of the experiment. However, taking into account our results and the results of other authors cited in this discussion, it can certainly be assumed that, indeed, TiO_2_ influences the growth of the discussed microorganisms. As reported by Sani et al. [13], the minimum inhibition concentrations of TiO_2_ nanoparticles (anatase, purity < 99%) for *L. monocytogenes* (IBRC-M 10,671), *E. coli* O_157_:H_7_ (IBRC-M 10,698), and *S. enteritidis* (IBRC-M 10,954) were 2, 3, and 3 mg/mL, respectively. *Bacillus subtilis* (wild type 3610 strain) growth was slightly decreased by 13 μg/mL of TiO_2_ NPs (Sigma-Aldrich, 700,347, anatase:rutile 80:20, < 150-nm particle size), after 72 h of cultivation (zones of inhibition in soft-solid agar LB medium) [14]. Both food-grade E171 TiO_2_ NPs and Aeroxyde P25 (NM-105, mixed crystalline, 85% anatase:15% rutile, mean particle diameter of 22 ± 1 nm) were added at 32–320 μg/mL to batch cultures in non-irradiated conditions induced dose-dependent inhibition of the growth of *E. coli* K12 MG1655. In the case of food-grade E171 at 320 μg/mL, TiO_2_ NPs caused complete inhibition of the growth of *E. coli* after 20 h of the cultivation [15]. Pure TiO_2_ NPs (*D* = 13 nm) inhibited the growth of *E. coli* and *Enterobacter cloacae*, as shown using the disc diffusion method, but no sophisticated calculations using statistical tools were performed in that study [16].

In another study, TiO_2_ NPs (40 ± 10 nm, 60 ± 10 nm, and 80 ± 10 nm) were tested against *S. typhimurium* and *E. coli*. At 50 μg/mL, the 40 nm, 60 nm, and 80 nm TiO_2_ NPs induced 3.96, 4.45, and 7.15% cell death, respectively. At the concentration of 250 μg/mL, TiO_2_ NPs caused a 10% increase in the killing rate, compared to 50 μg TiO_2_/mL, but the trend was similar at both concentrations applied. Similar results were obtained for the *S. typhimurium* population, where the killing percentage was nearly 3% at the 50 μg/mL concentration and varied to 2 decimal places only. However, in the S*. typhimurium* population treated with TiO_2_ NPs with mean diameters of 60 nm and 80 nm, the killing rate increased 2–threefold along with the higher dose of NPs applied (250 vs. 50 μg NPs/mL) [17]. Khan et al. [18] isolated from chocolate and characterized TiO_2_ NPs with an average size of ~ 40 nm. The isolated TiO_2_ NPs decreased the growth of probiotic bacteria (*Bacillus coagulans*, *Enterococcus faecalis*, and *Enterococcus faecium*) over a concentration range of 125, 250, and 500 μg/mL in vitro (plate method). At the concentration of 500 μg TiO_2_/mL, NPs inhibited the growth of the probiotic formulation by 66 ± 6.1% and 71 ± 5.6% in anaerobic and aerobic conditions, respectively. Significant reduction in the count of *E. coli* (ATCC 8739), *Salmonella paratyphi* (ATCC 9150), and *L. monocytogenes* (ATCC 15,313) (reduction log CFU 0.95 ± 0.01, 0.66 ± 0.01, and 0.58 ± 0.01, respectively) by TiO_2_ NPs (15–50 nm, 0.1 mg/mL in the solution) was observed by Anaya-Esparza et al. [19]. Ripolles-Avila et al. [20] reported that both anatase (crystal phase, 7 nm) and a combination of anatase–rutile (80:20 wt/wt, 21-nm size) in the range from 0.78 to 100 μg TiO_2_/mL of nutrient broth significantly reduced the growth of *S. enterica* var. *enteridis*, *E. coli*, *S. aureus*, and *B. cereus* in a dose-dependent manner. Sroila et al. [21] prepared 5-, 16-, and 26-nm TiO_2_ NPs and showed that, in the dark, 1–6 nm TiO_2_ NPs (crystal form of anatase, mean diameter 5 nm, at the highest concentrations applied equally to ~ 22.73 μg/mL) were efficient inhibitors of the growth of *E. coli* after 4 h of incubation. The authors concluded that TiO_2_ NPs can diffuse into the cell membrane of *E. coli* and disorganize the cell membrane, leading to deformation of the cell and disorganization of intracellular structures. The minimum inhibition concentrations of TiO_2_ NPs (purity 99%, tetragonal shape, 10 ~ 25 nm in diameter) for the growth of *E. coli*, *S. enteritidis*, and *L. monocytogenes* were 2.00 ± 0.33, 2.50 ± 0.17, and 1.00 ± 0.14 mg/mL, respectively [22]. Lately, Arezoo et al. [23] reported an inhibitory effect of TiO_2_ NPs (with particle size < 20 nm), incorporated in sago-based starch film on the growth of *E. coli* and *Salmonella typhimurium.* However, in their experiments, the film was complex and cannot be directly compared with our studies.

As it can be seen, the above-cited authors reported on the reduction in the growth of bacteria in the presence of TiO_2_. In selected cases, we obtained similar results as other authors, concerning the effective concentrations of TiO_2_ that may have an influence on bacterial growth [15, 17, 20], 22. However, there are also some discrepancies between our results and other, above-cited works [13, 14, 19, 21]. These differences can be due to several reasons that must be discussed. First, the authors used various methods of bacterial detection (qualitative disc method, zones of inhibition, absorbance measurement, etc.). Secondly, various titanium dioxides were used (food-grade mixtures from various producers, with an undefined particle size distribution, or TiO_2_ NPs with a well-defined size in a very narrow range). Thirdly, although we and the cited authors have tested bacteria of the same genus, the outcome may be species/strain dependent. Due to all these factors, the comparison of the results must be made with the awareness of their existence. Another issue is the analysis of how TiO_2_ affects the growth of bacteria. Unfortunately, we do not have more detailed information yet, what factors determine the reduction of growth and propagation of bacteria in the presence of TiO_2_, this knowledge is also not included in the cited works.

Last but not least, some studies demonstrated that TiO_2_ NPs had no influence on the growth of discussed microorganisms. *Escherichia coli* (ATCC8099) was not inhibited by P25 TiO_2_ NPs (anatase–rutile, 21 ± 5 nm) at 0.15 mg NPs/plate [24]. Similarly, Liu et al. [25] observed no inhibition of the growth of *E. coli* by pure TiO_2_ NPs at 0.5 g/L in the dark and under the light. Rokicka-Konieczna et al. [26] showed no inhibition of the growth of *E. coli* K12 (ATCC 29,425) in the dark in the presence of 0.1 g/L of TiO_2_ NPs (anatase, crystallite size 14 nm). Sethi and Sakthivel [27] showed no inhibition of the growth of *E. coli* by pure anatase (titania) in the dark in the presence of 0.5 mg/mL of TiO_2_ NPs. Aguas et al. [28] showed no inhibition of the growth of *E. coli* by commercial TiO_2_ (P25, anatase/rutile = 80/20) suspended in the solution at the concentration of 50 mg TiO_2_ NPs/L. No inhibition of the growth of *E. coli* and *S. paratyphi* A in the dark was observed in the presence of TiO_2_ NPs [29]. The average nanoparticle size specified by the authors based on their previous study was 8–20 nm [30].

## Conclusion

The complexity and variability of microbiota species in each human impede the assessment of the effect of food additives on this ecosystem. The present study has demonstrated inhibition of bacterial growth caused by both food-grade E171 and TiO_2_ NPs in most of the analyzed strains, at concentrations similar to those reported by a number of other authors. However, there are also discrepancies between works, concerning the effective concentrations of TiO_2_. The methods of detection of microorganisms, the type of TiO_2_ (food grade or NPs), or species/strain variability may influence the outcome of the study. Due to these factors, it is necessary to check the mechanism of action of TiO_2_ on the bacterial cell; therefore, further investigations in this field are indispensable for elucidation of the potential toxicity of NPs to the human microbiome.

## Supplementary Information

ESM1 (ZIP 8.20 MB)

ESM2 (DOCX 438 KB)

## Data Availability

Not applicable.
