# Supplementary material for: Effect of TiO2 on Selected Pathogenic and Opportunistic Intestinal Bacteria
Source: Biol Trace Elem Res. 2021 Jul 23;200(5):2468–74. doi: 10.1007/s12011-021-02843-7 (PMC9023387; doi:10.1007/s12011-021-02843-7)
Supplement: Supplementary file 1 — (ZIP 8.20 MB) [file 12011_2021_2843_MOESM1_ESM.zip › 2S6 (L. monocytogenes)_ESM.docx]

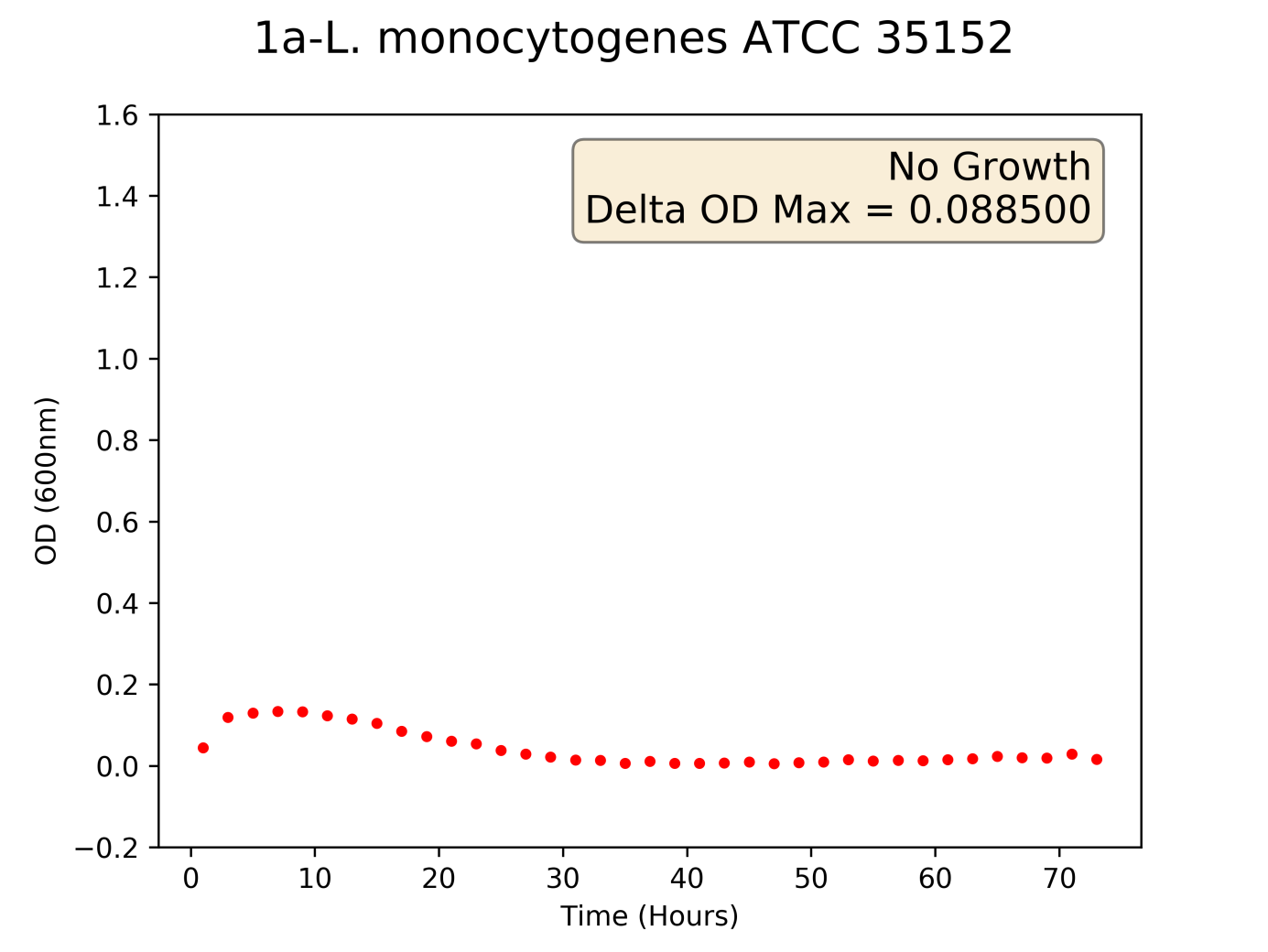


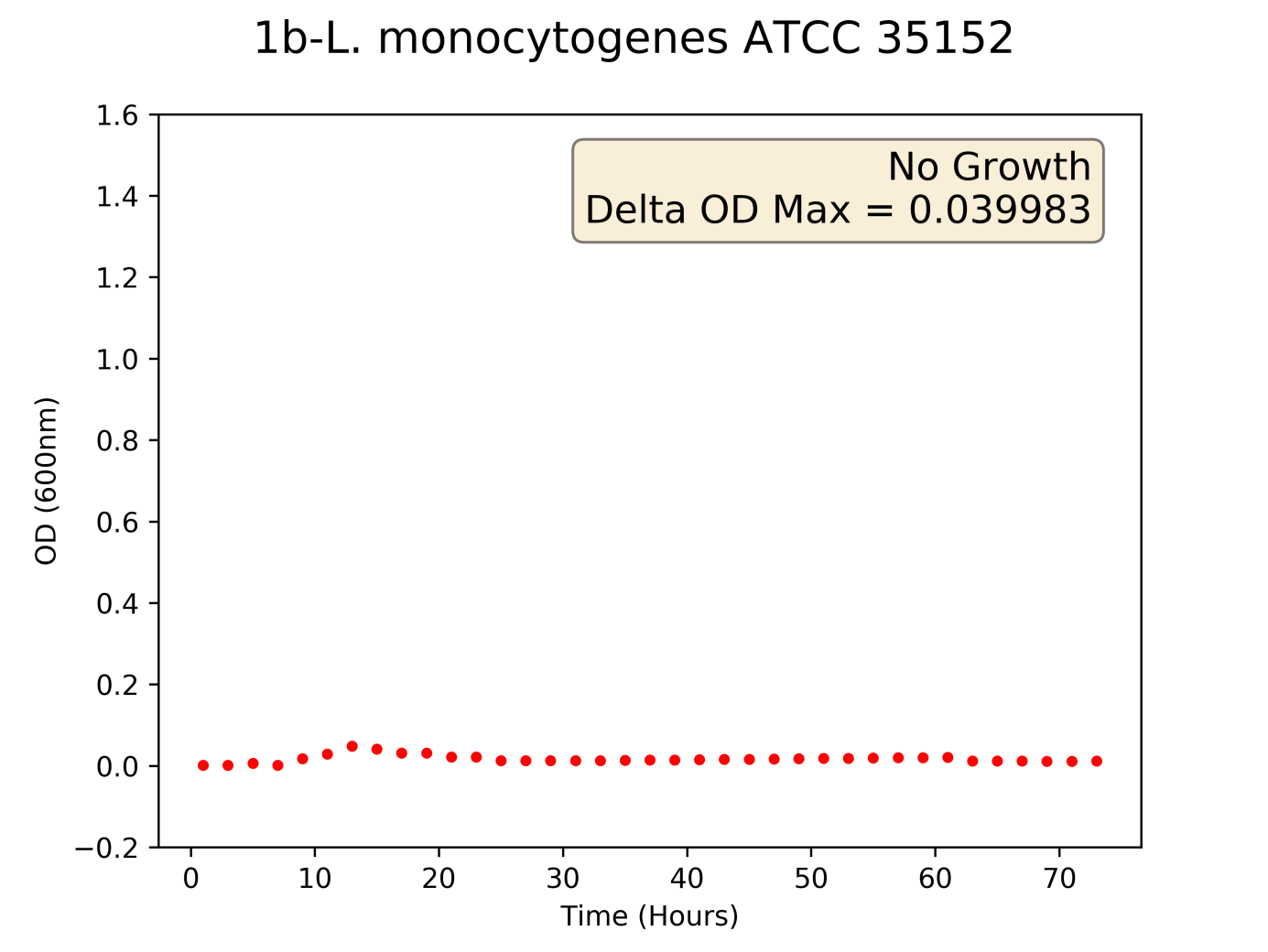


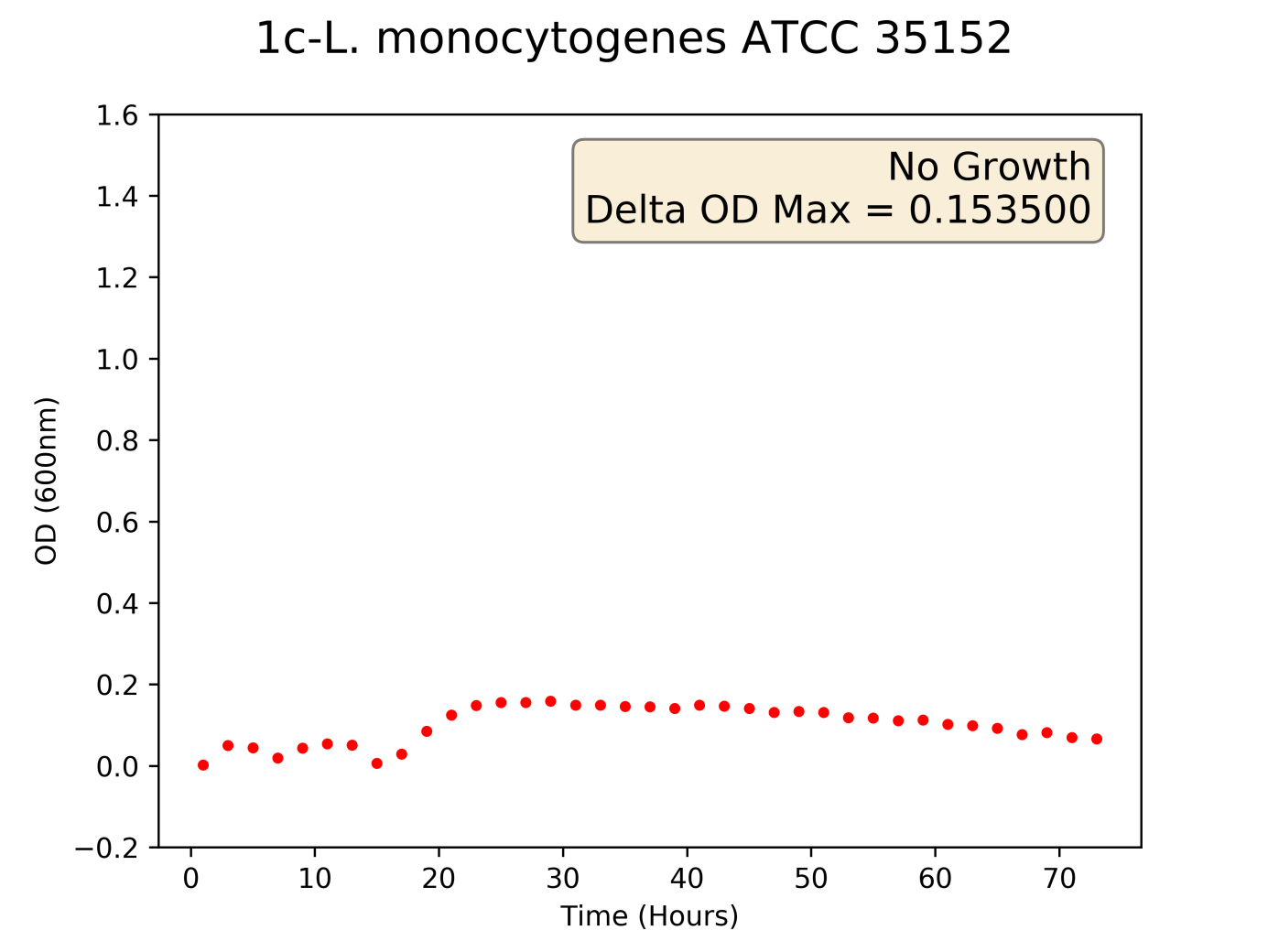


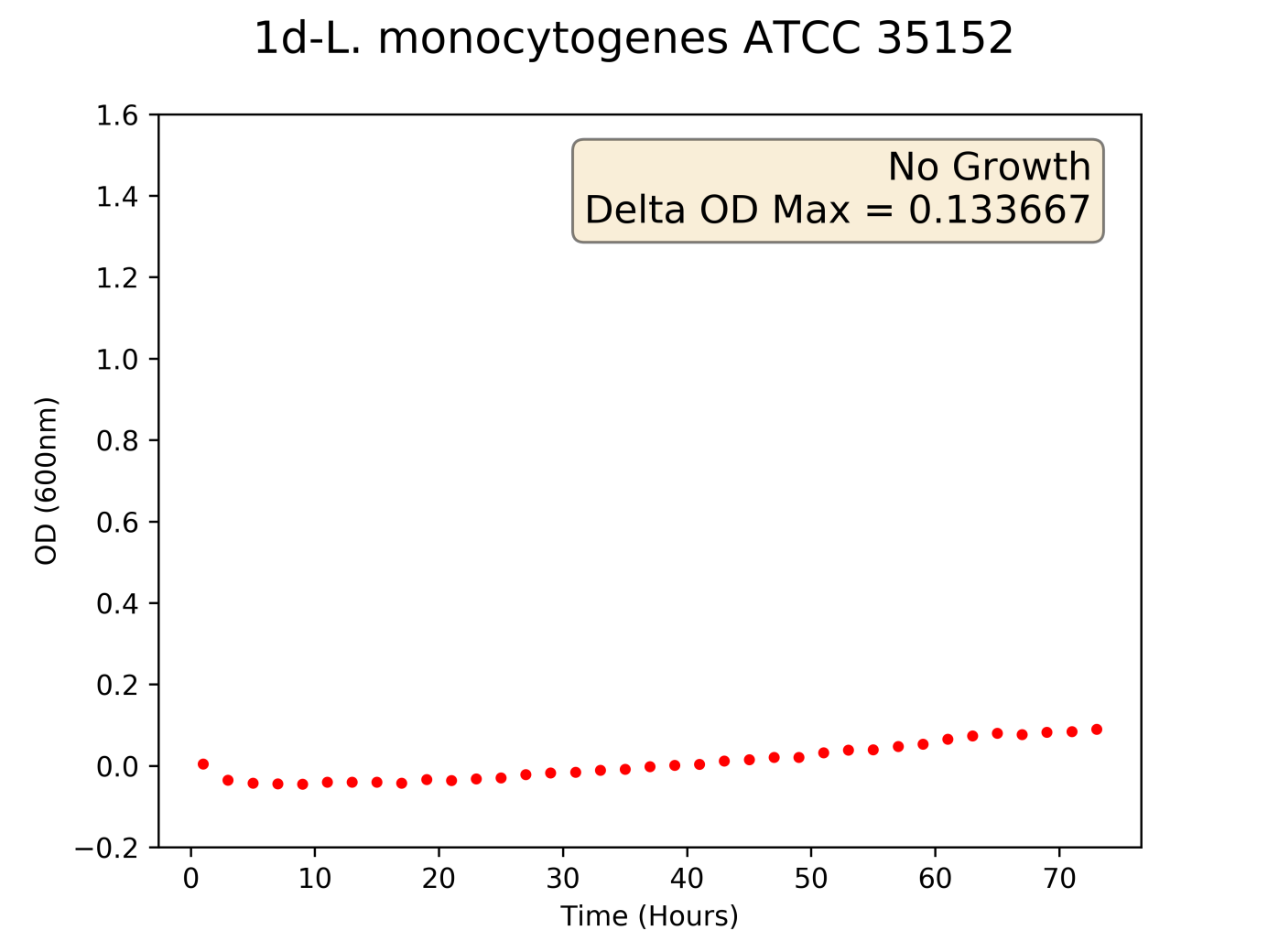


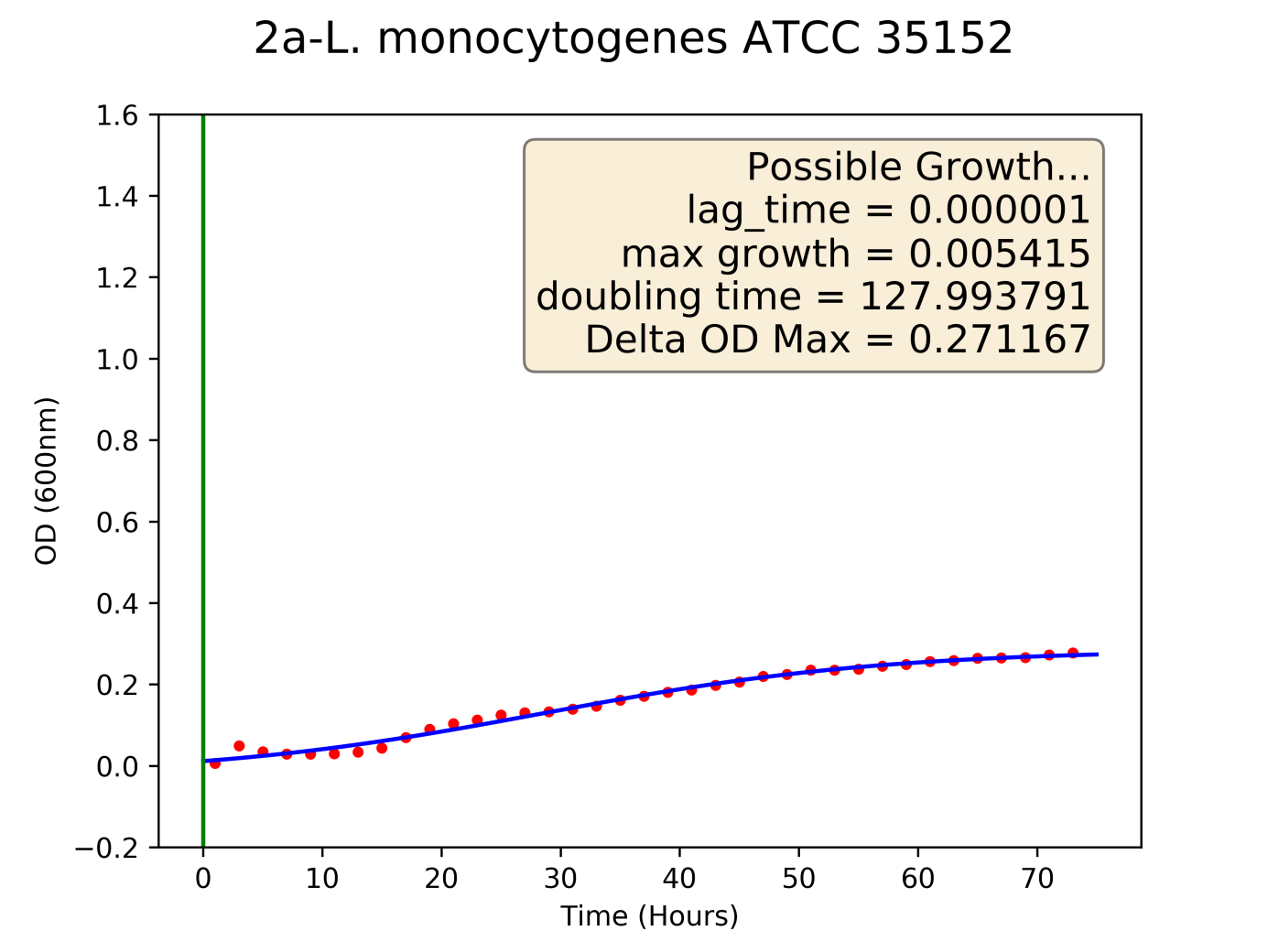


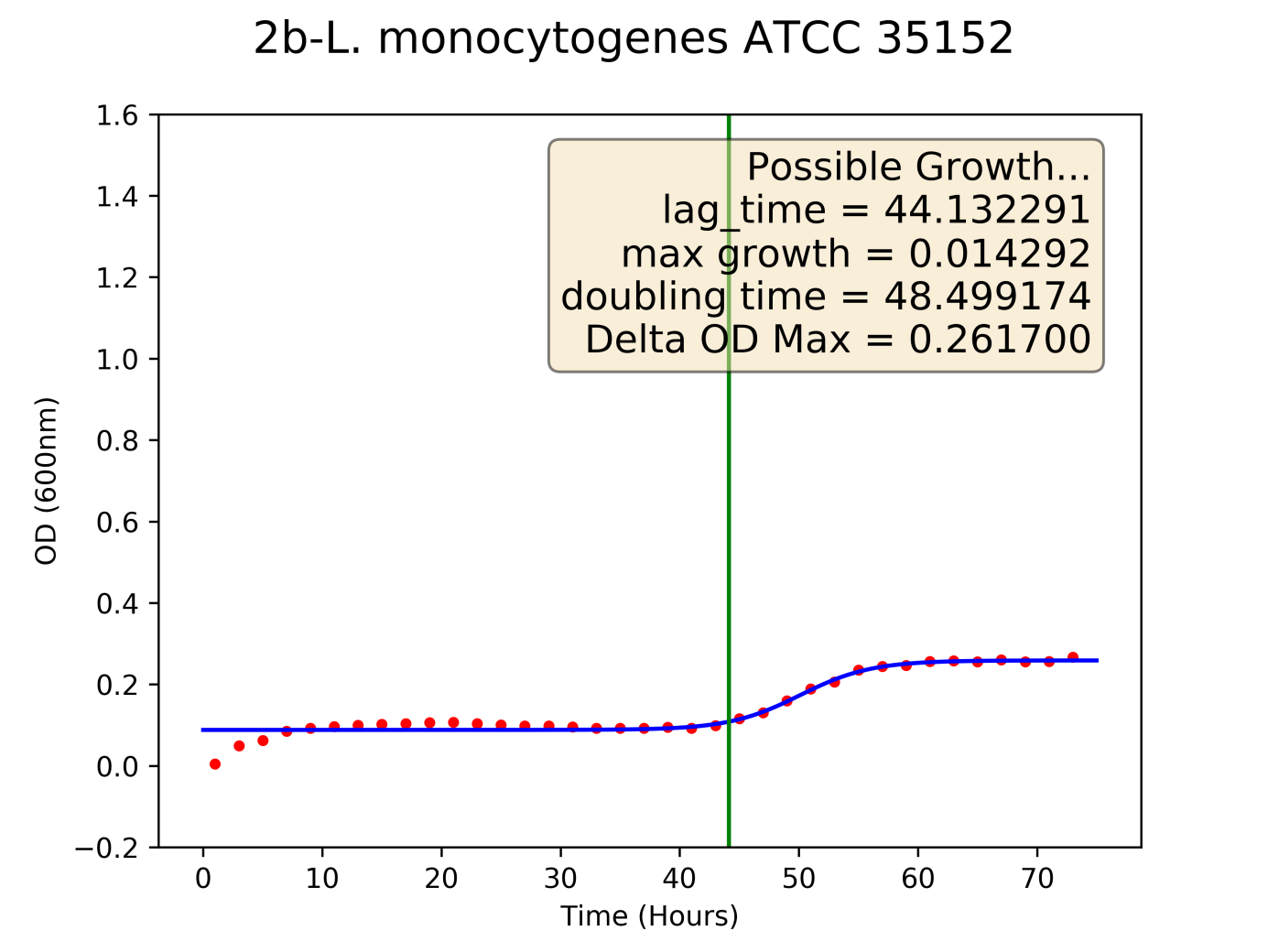


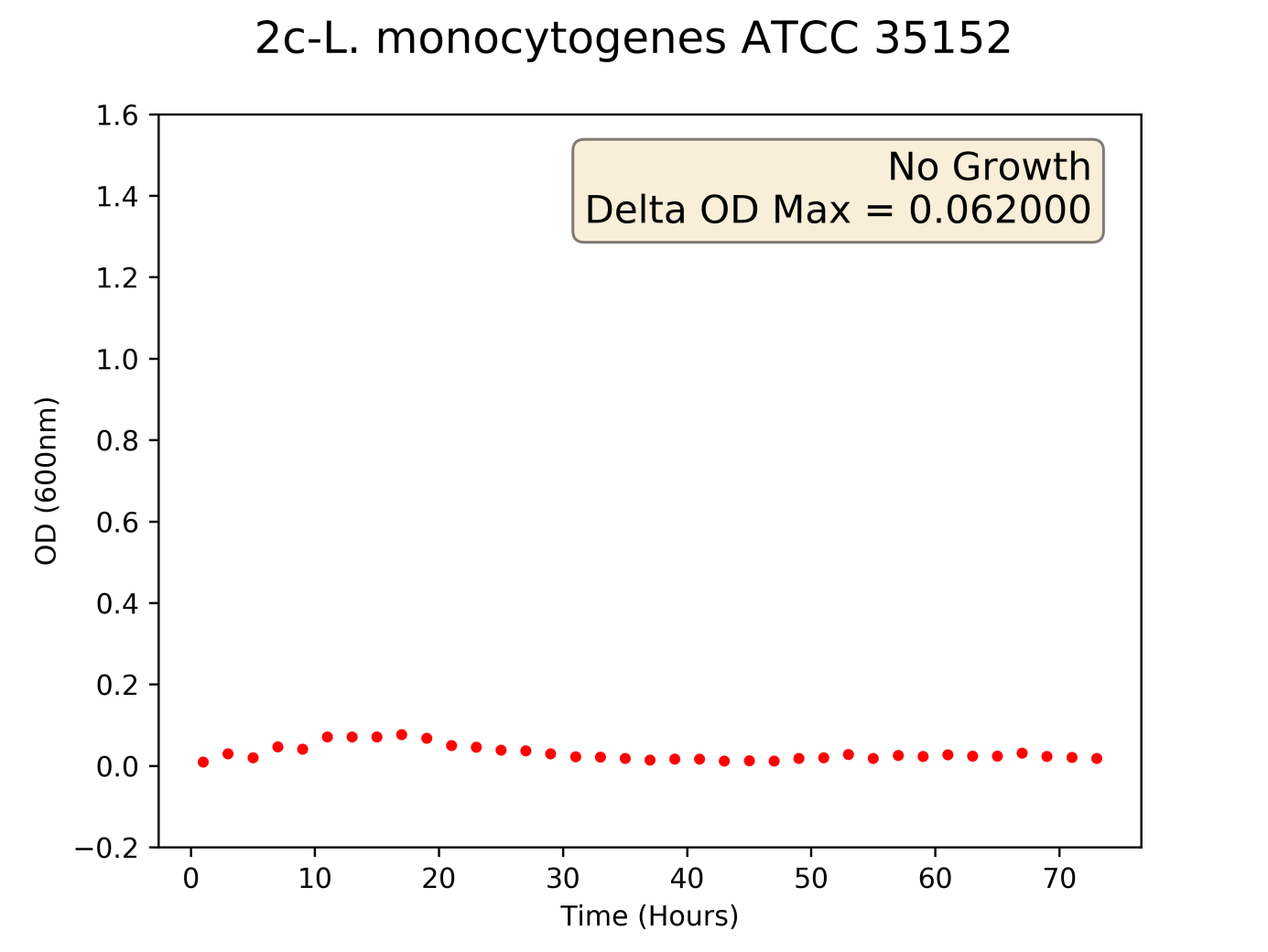


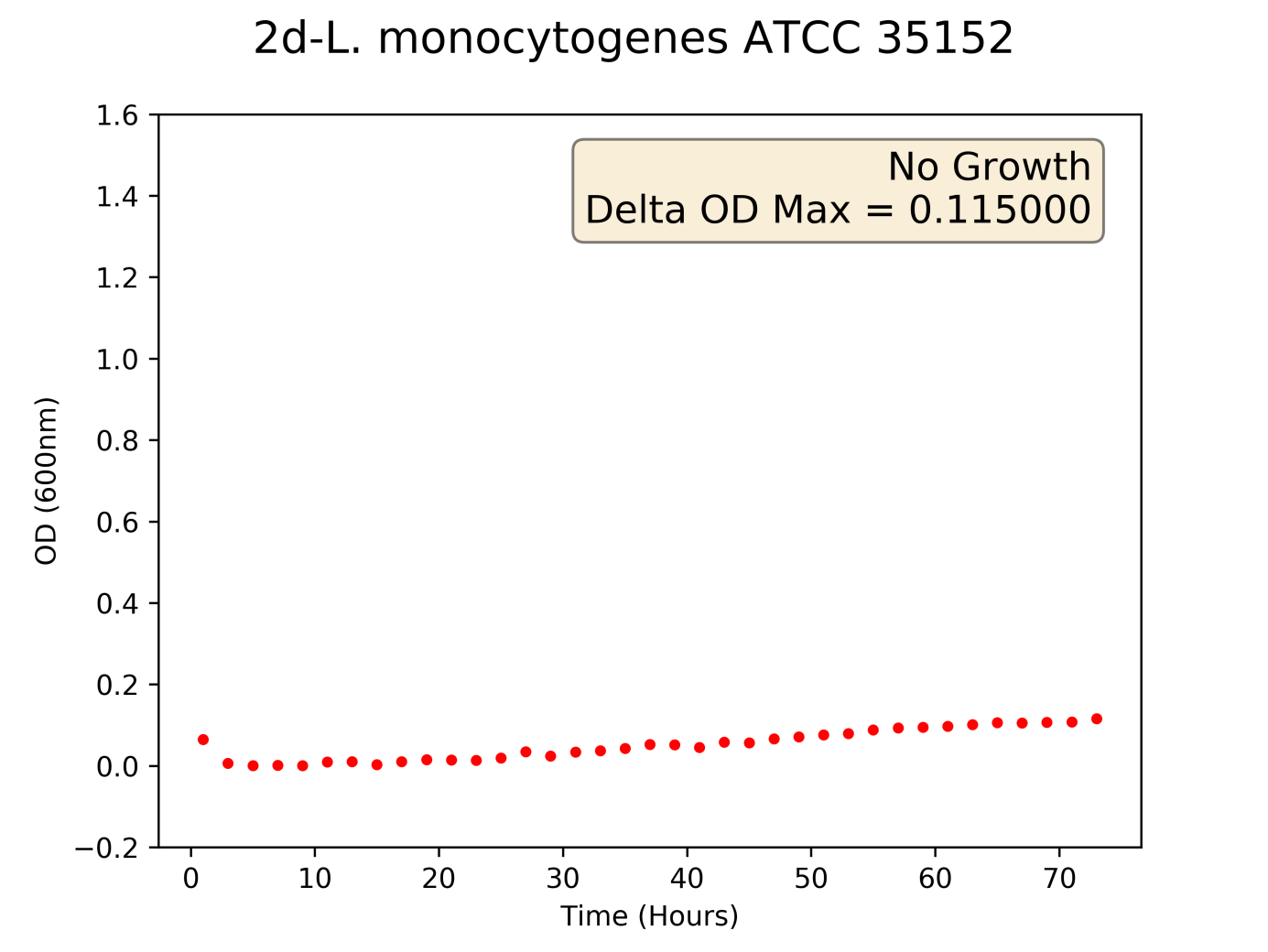


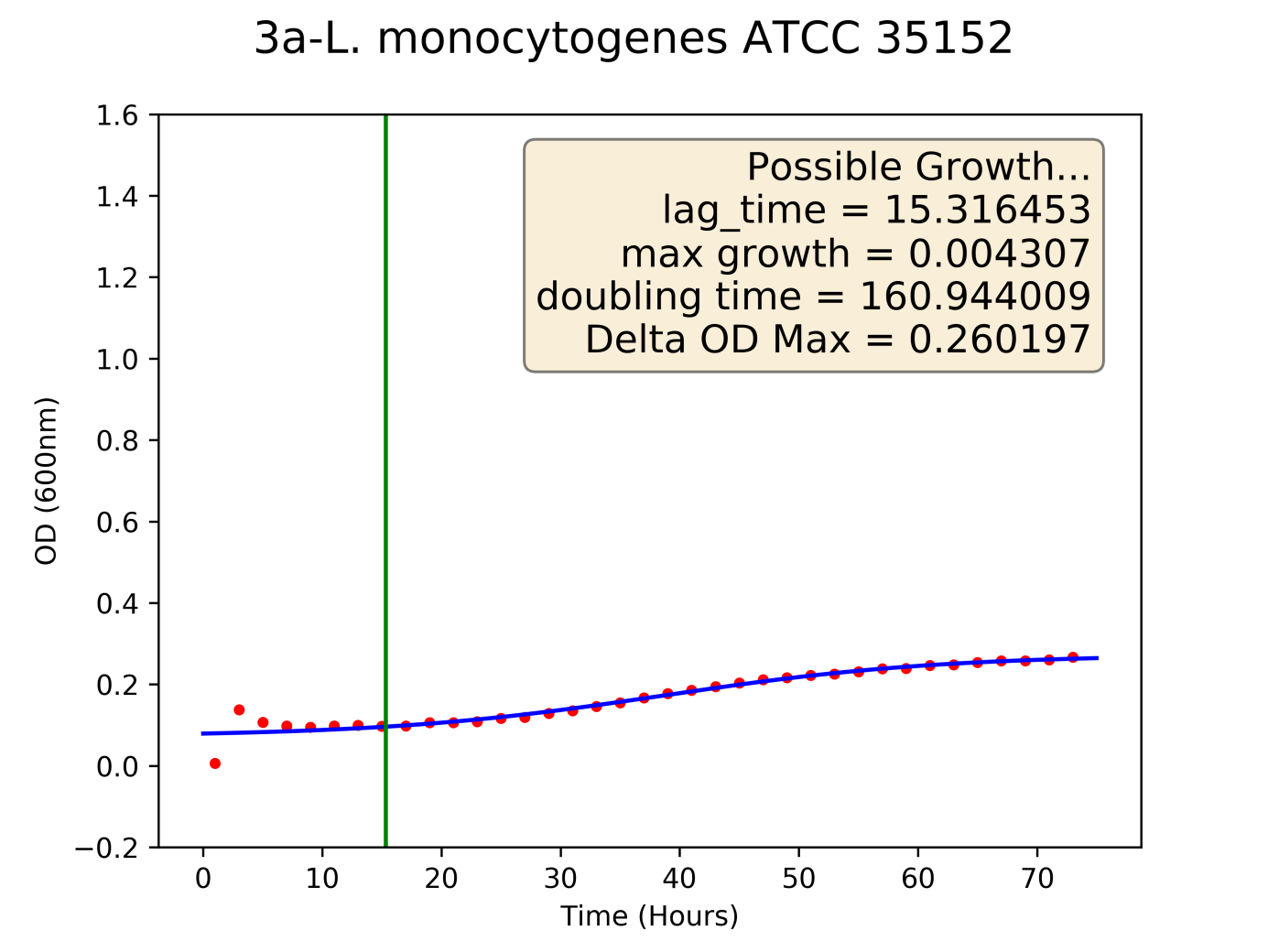


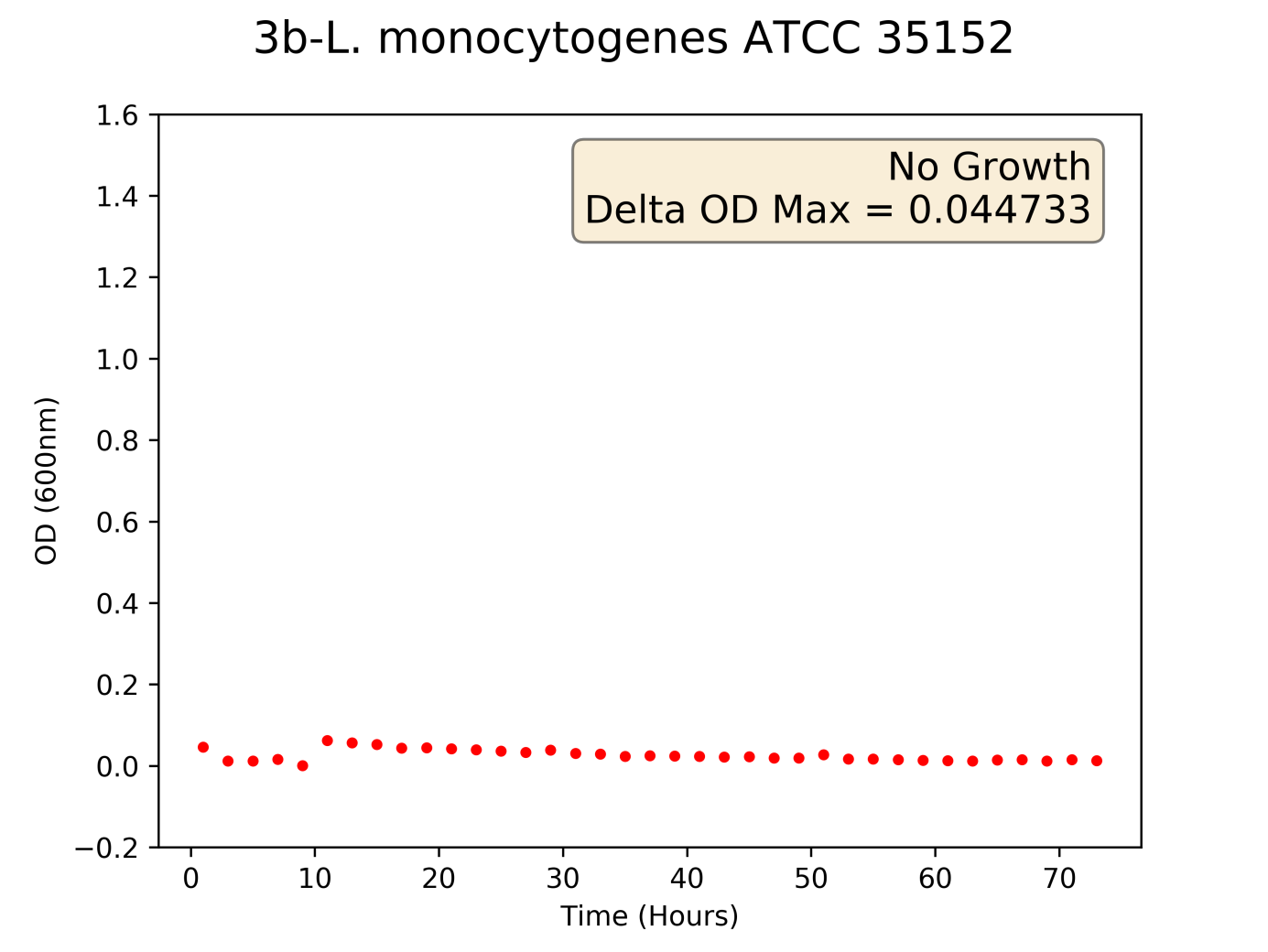


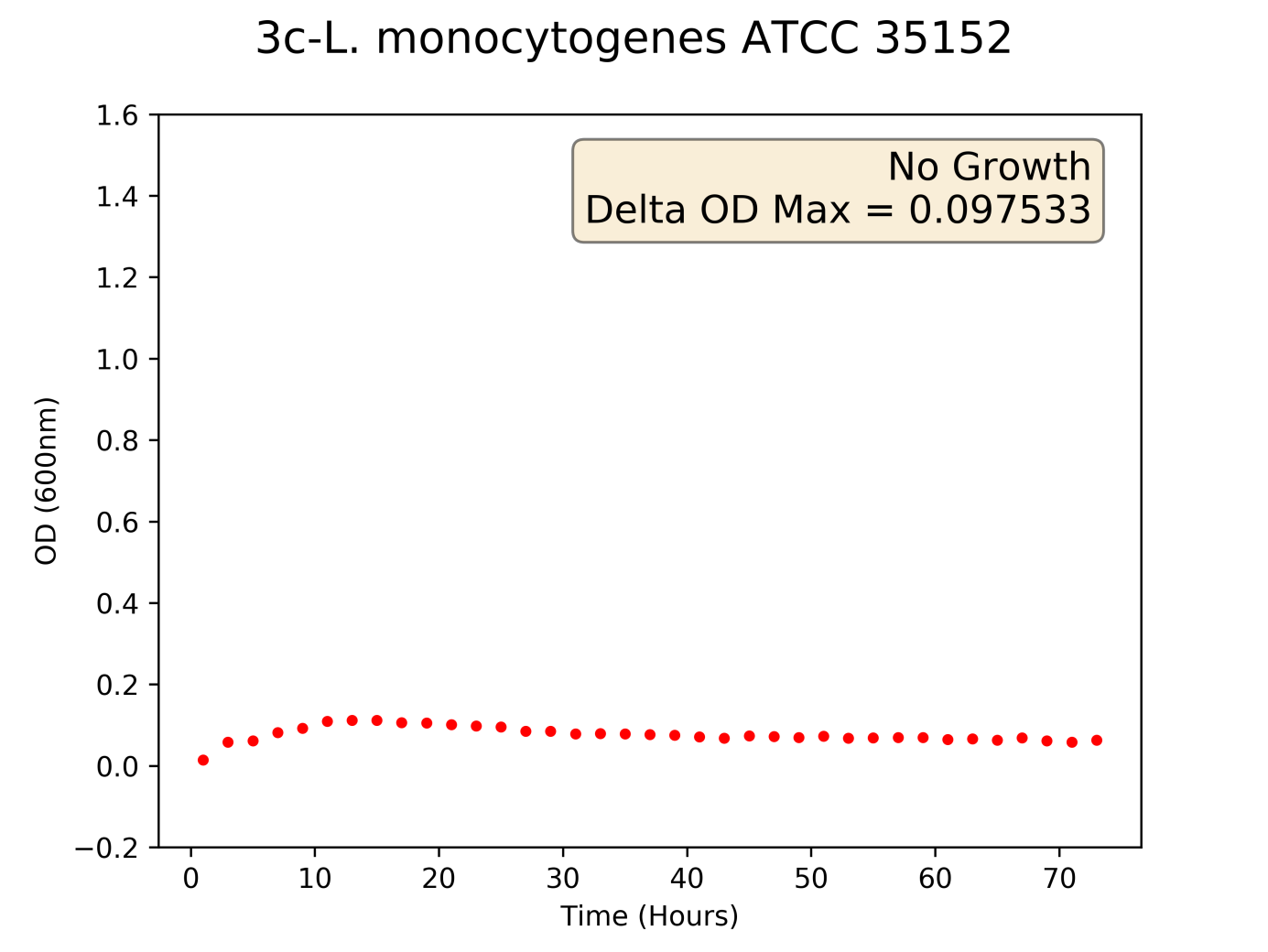


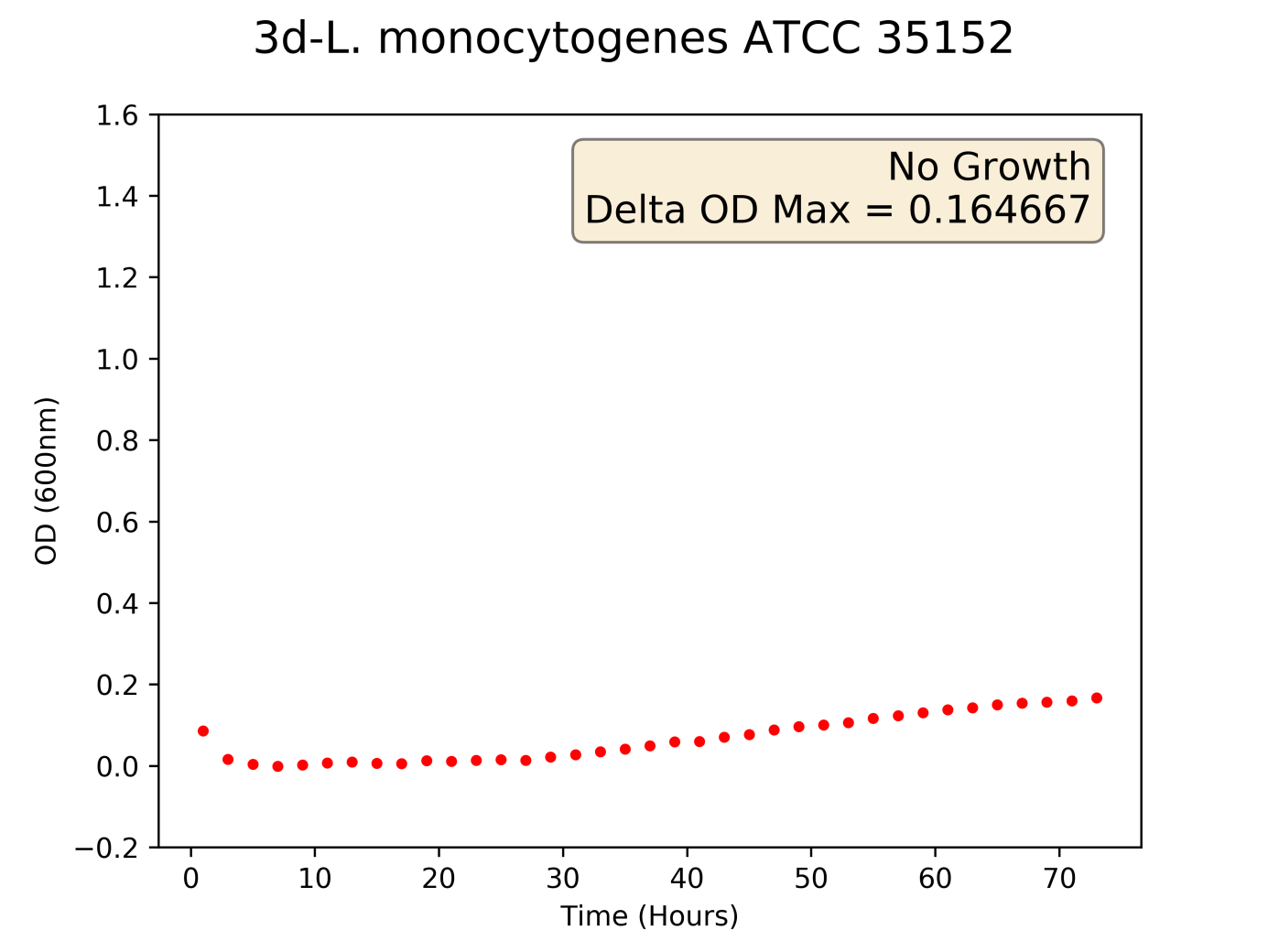


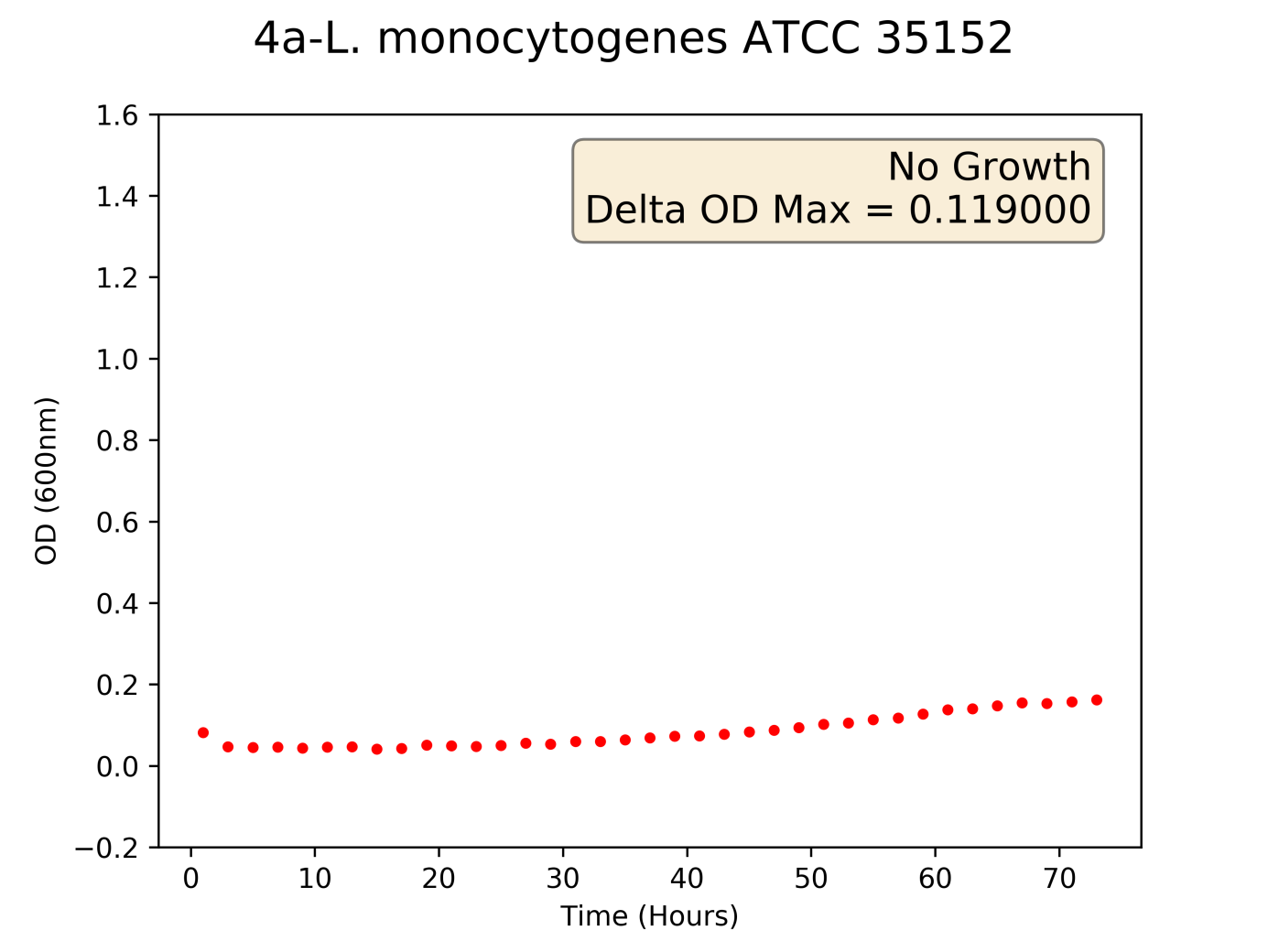


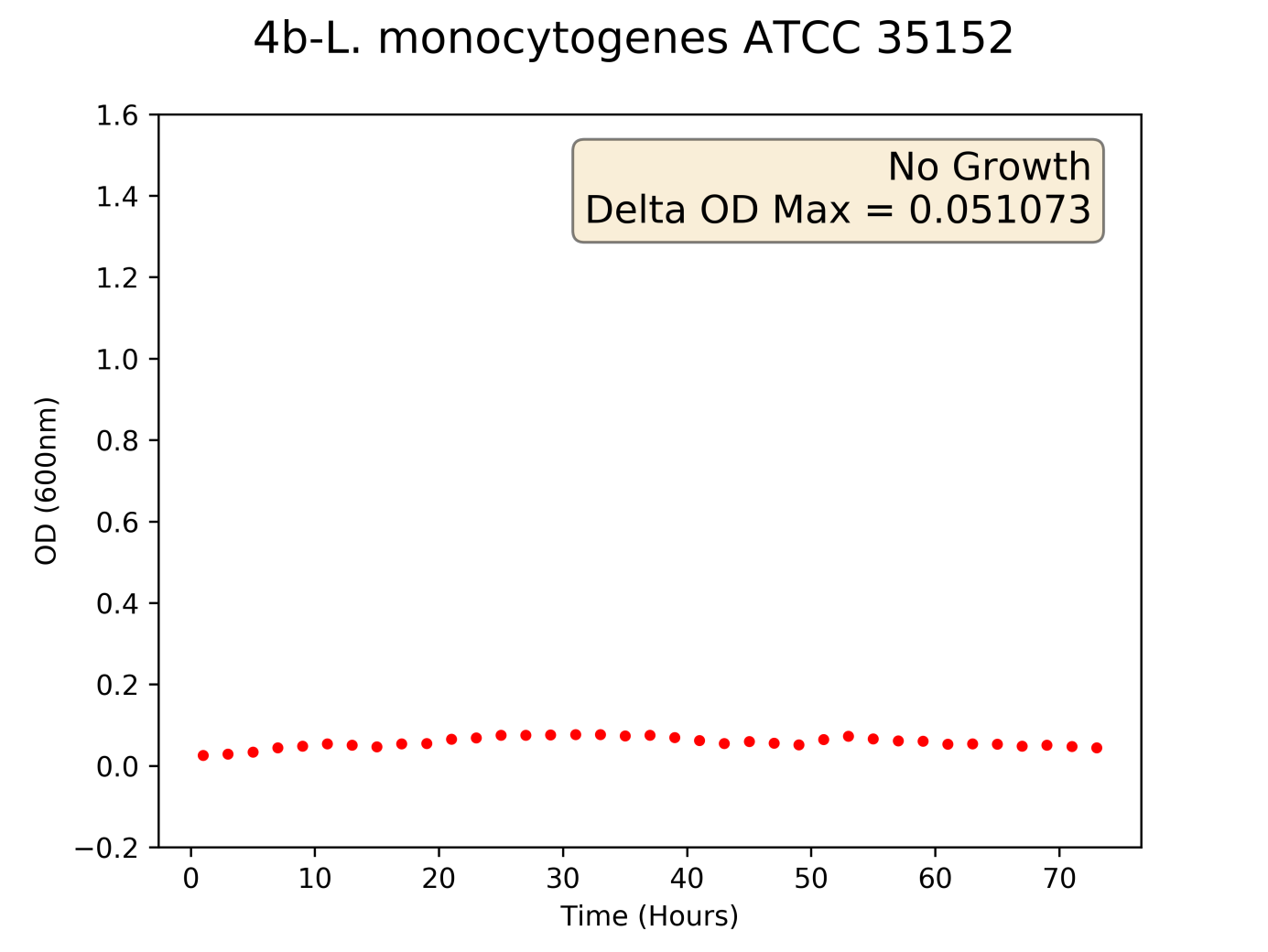


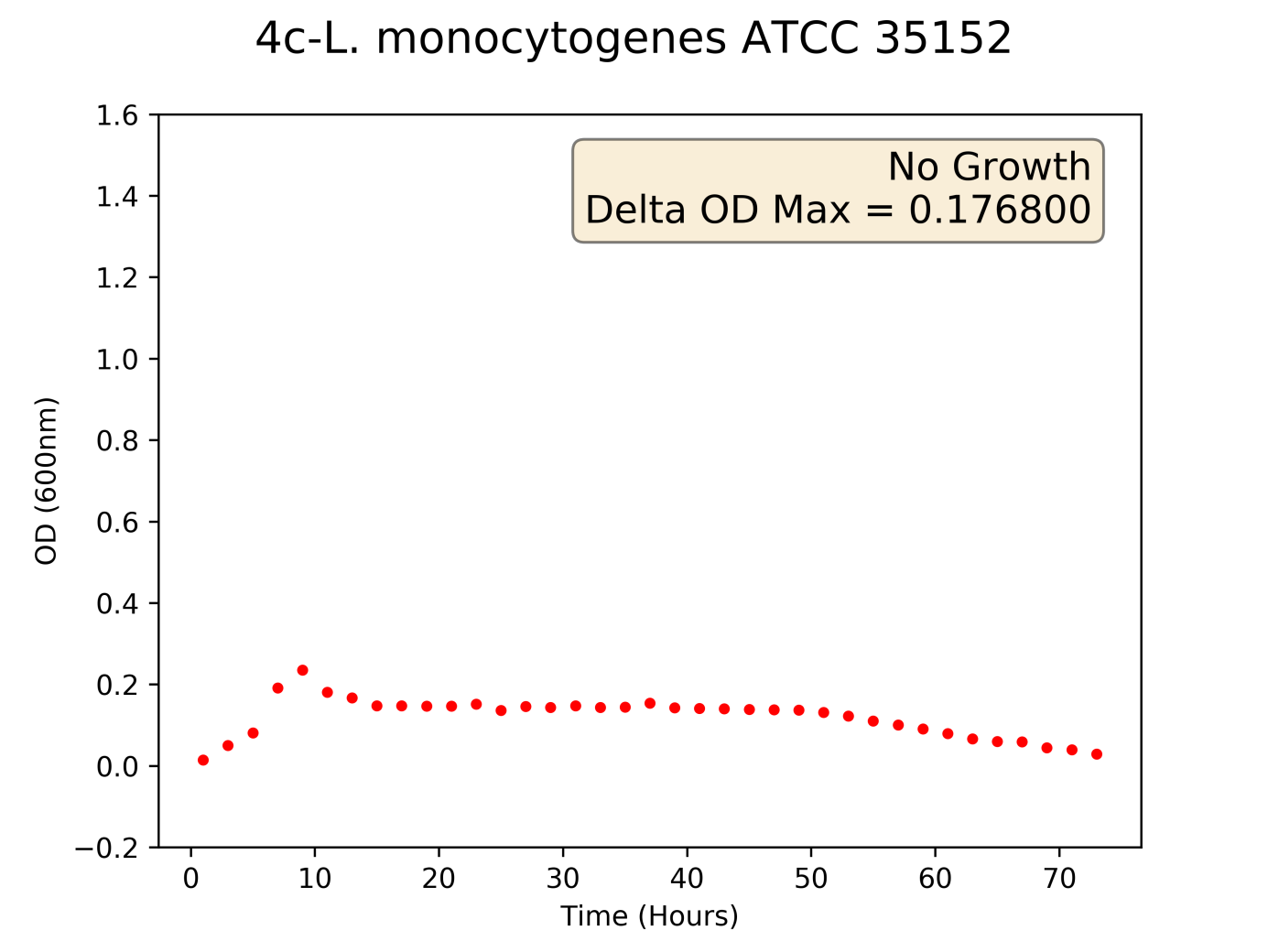


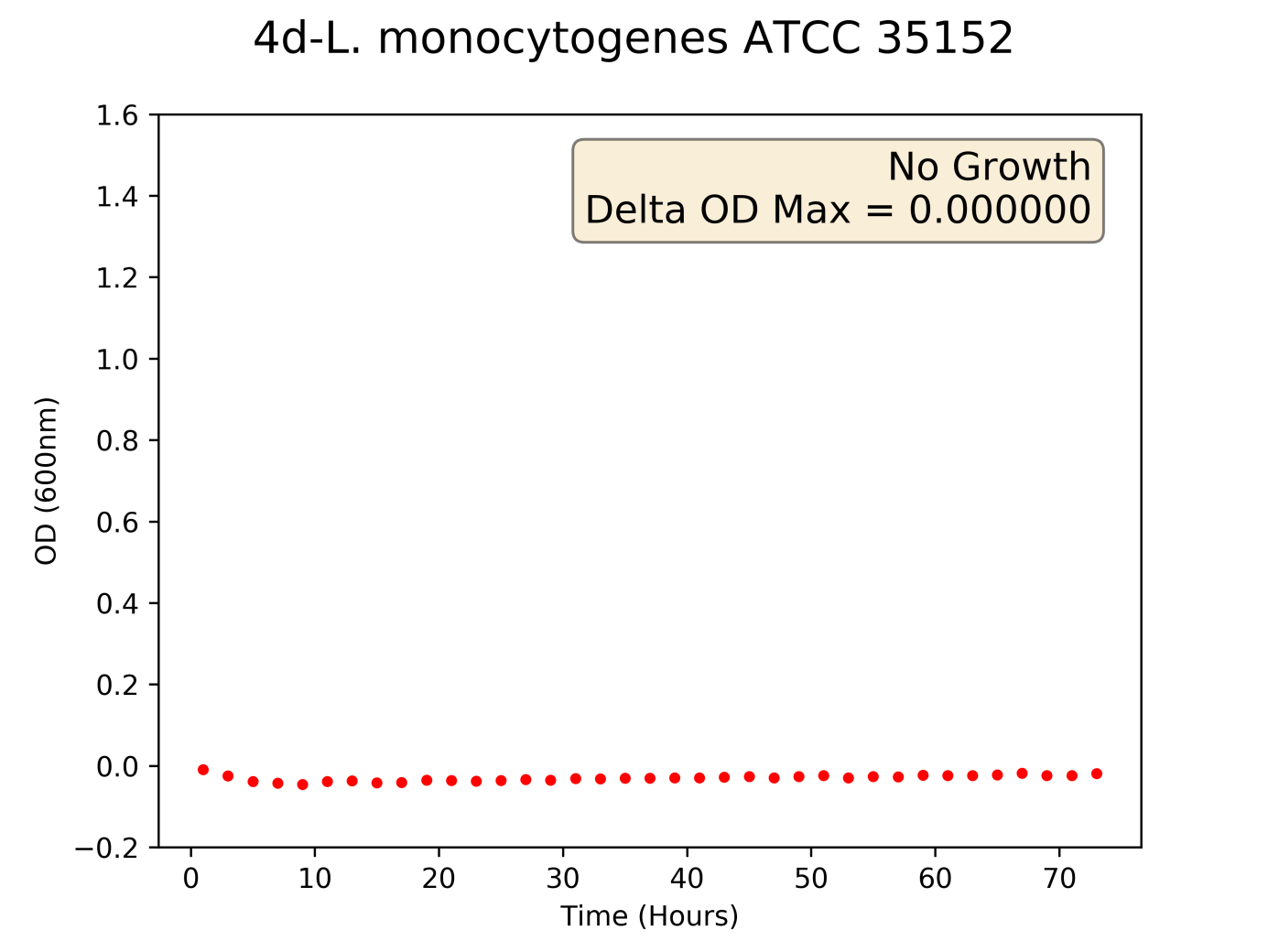


**Fig. S1.** Regression curves of selected data. *L. monocytogenes* (1, 2, 3, 4 – types of TiO_2_; a, b, c, d – concentration of TiO_2_ : 60, 150, 300 i 600 mg/L)
