# Supplementary figures and images for: Effect of TiO2 on Selected Pathogenic and Opportunistic Intestinal Bacteria
Source: Biol Trace Elem Res. 2021 Jul 23;200(5):2468–74. doi: 10.1007/s12011-021-02843-7 (PMC9023387; doi:10.1007/s12011-021-02843-7)

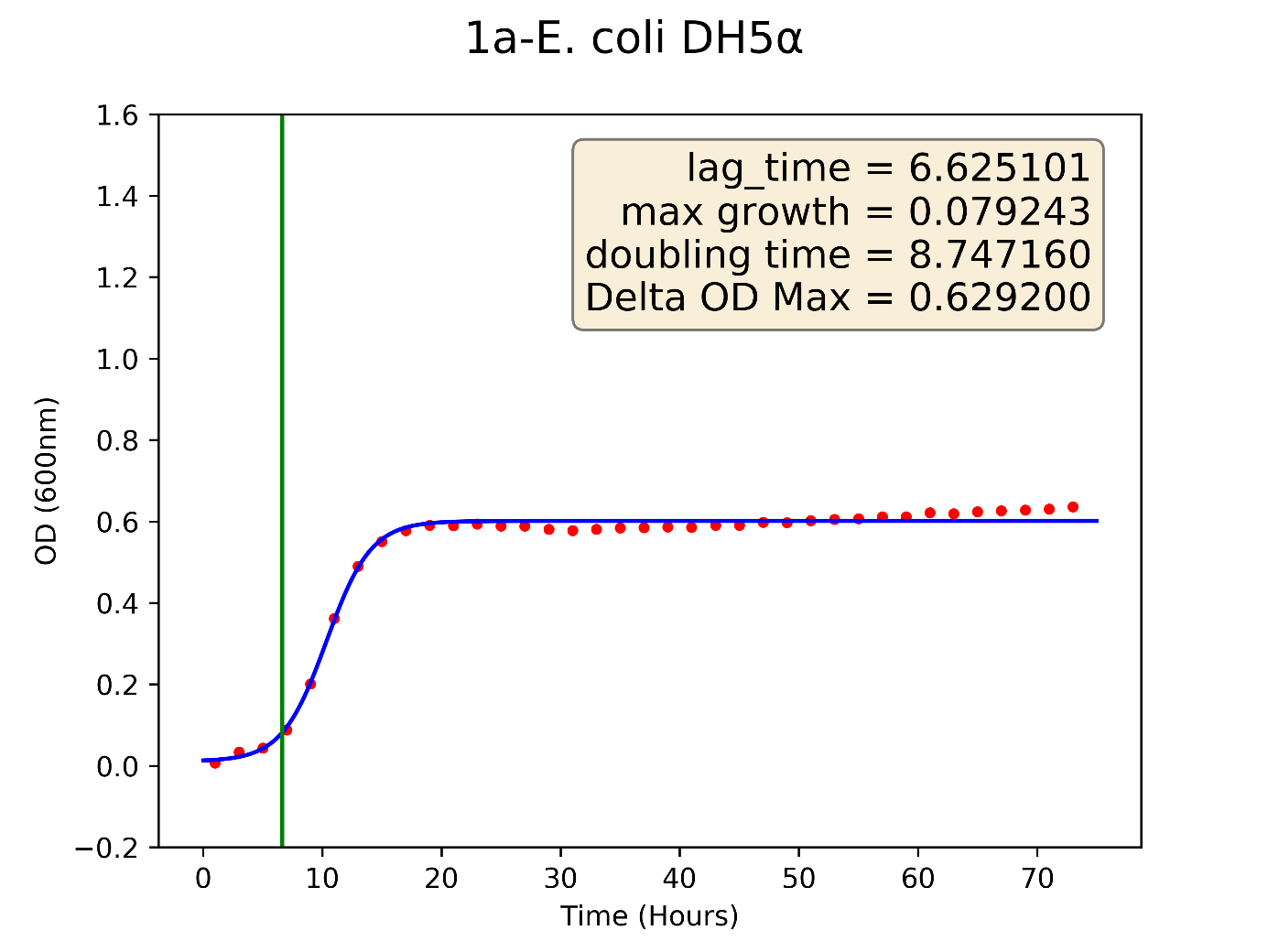


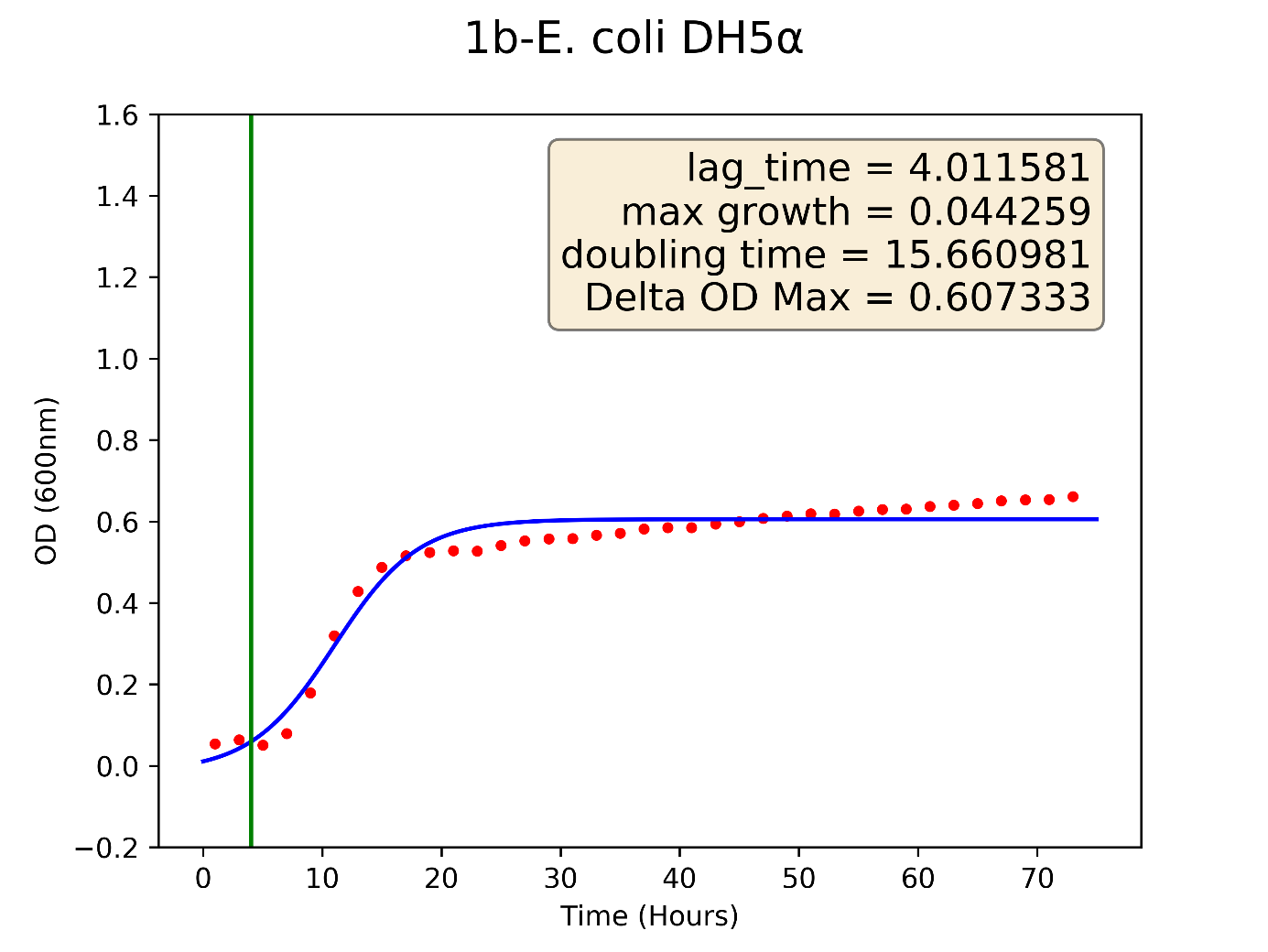


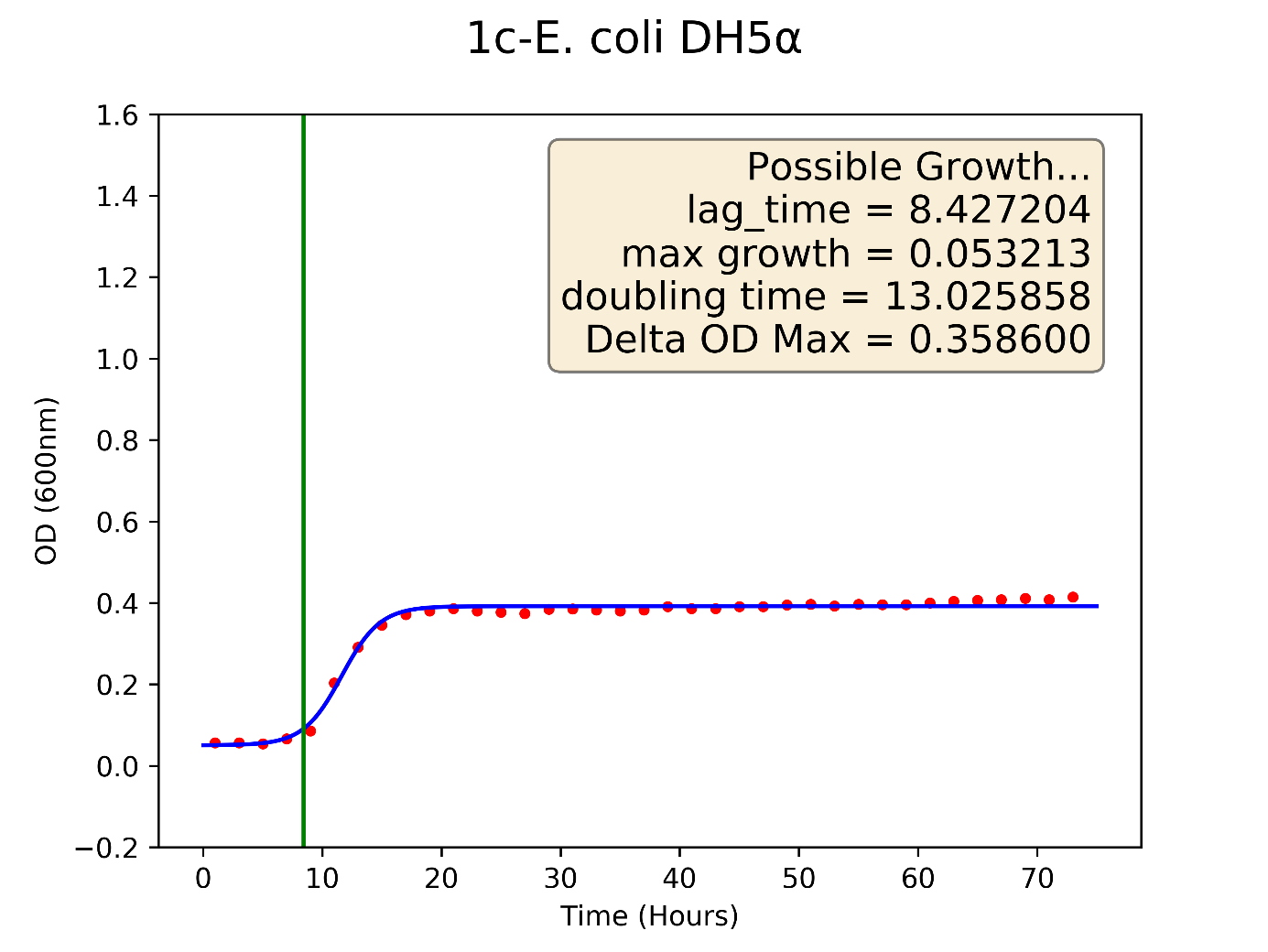


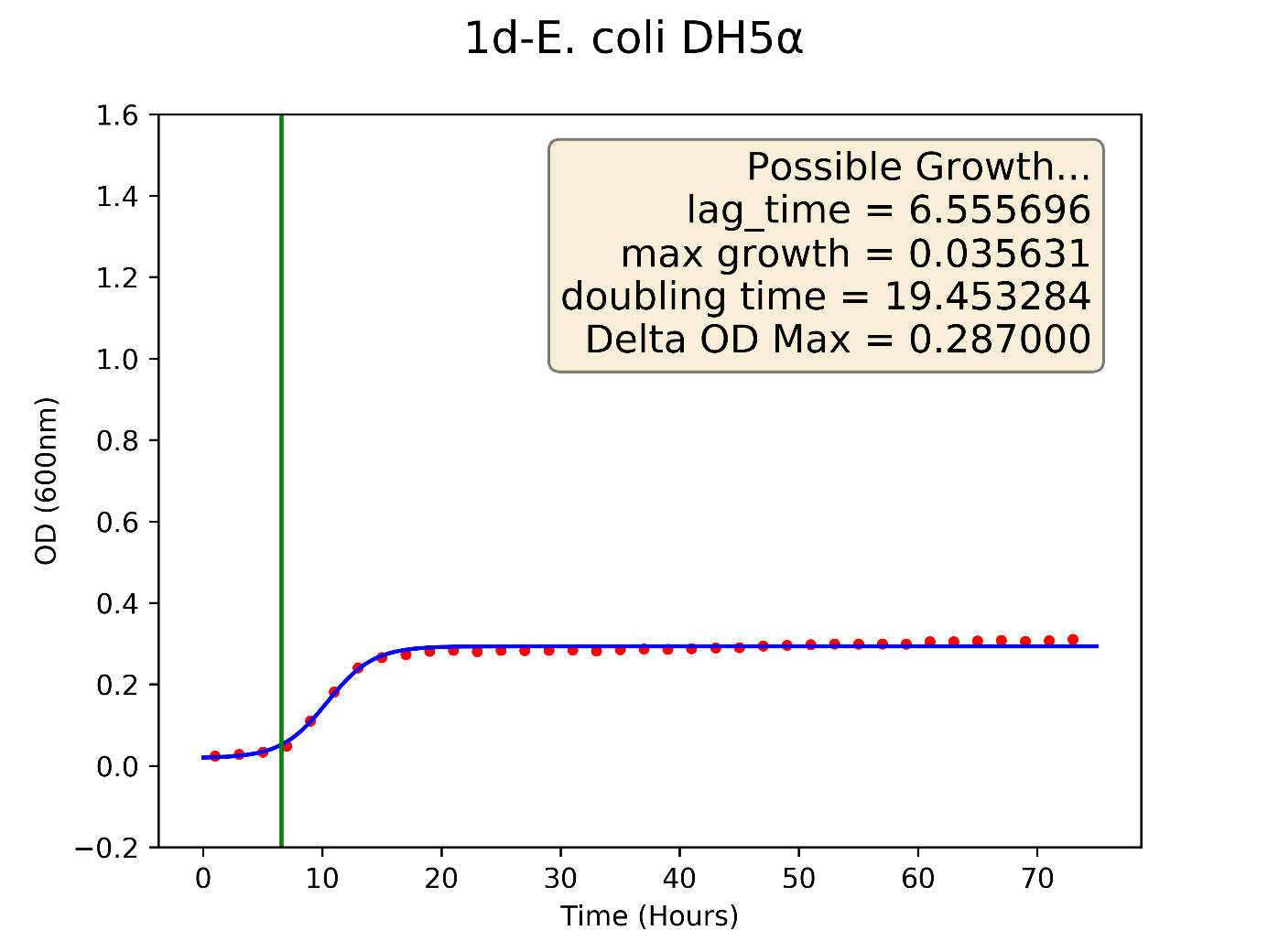


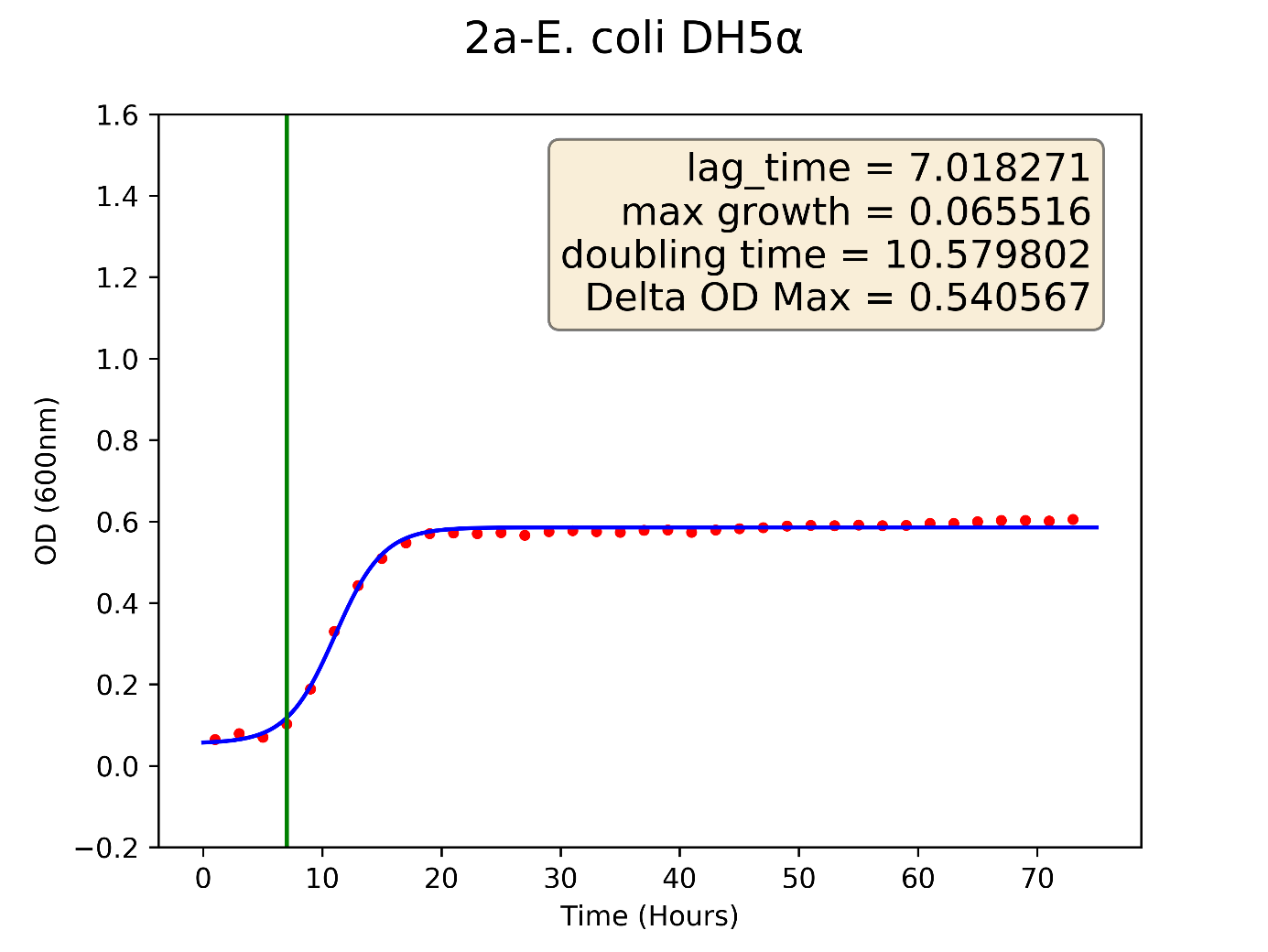


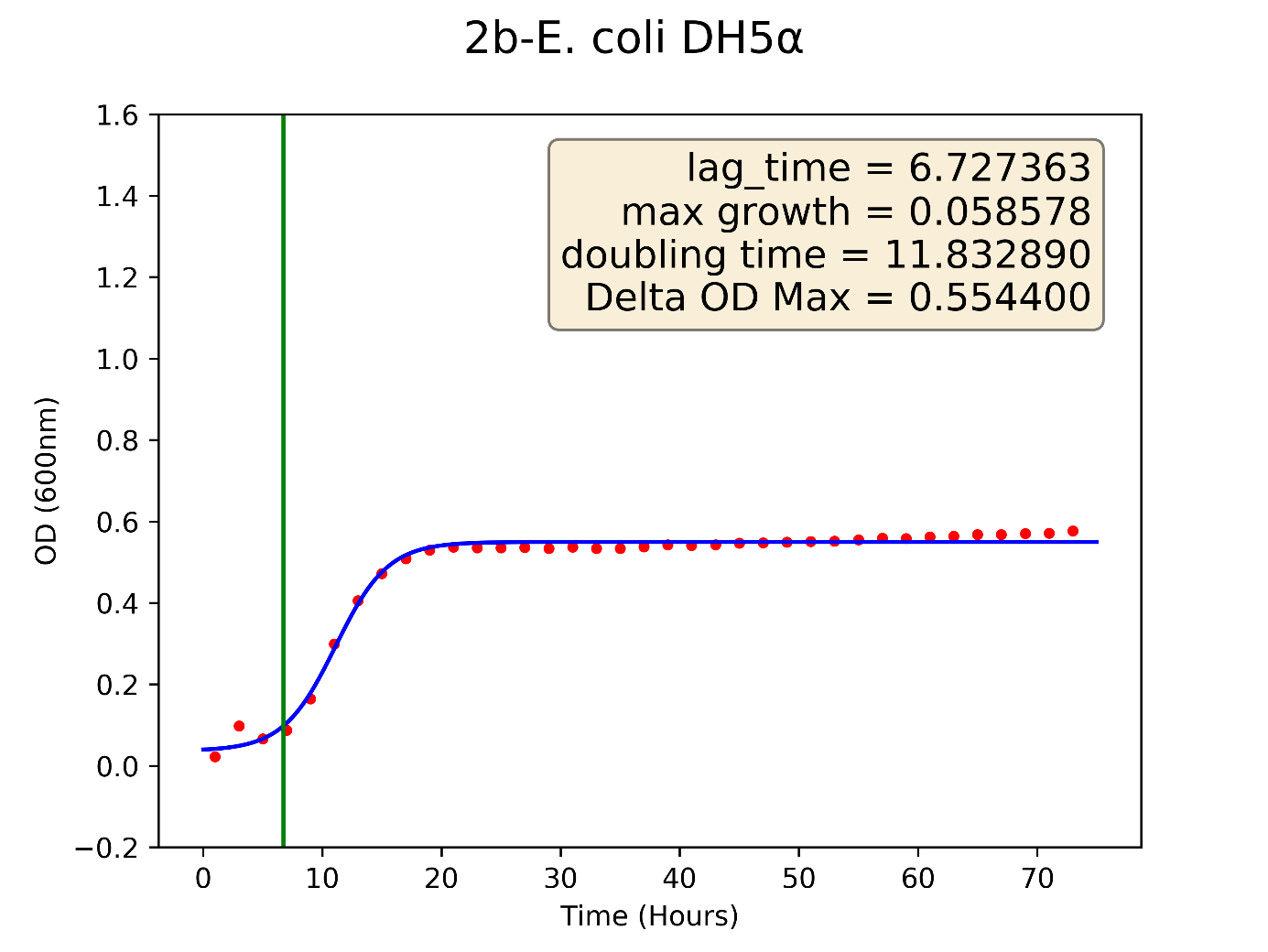


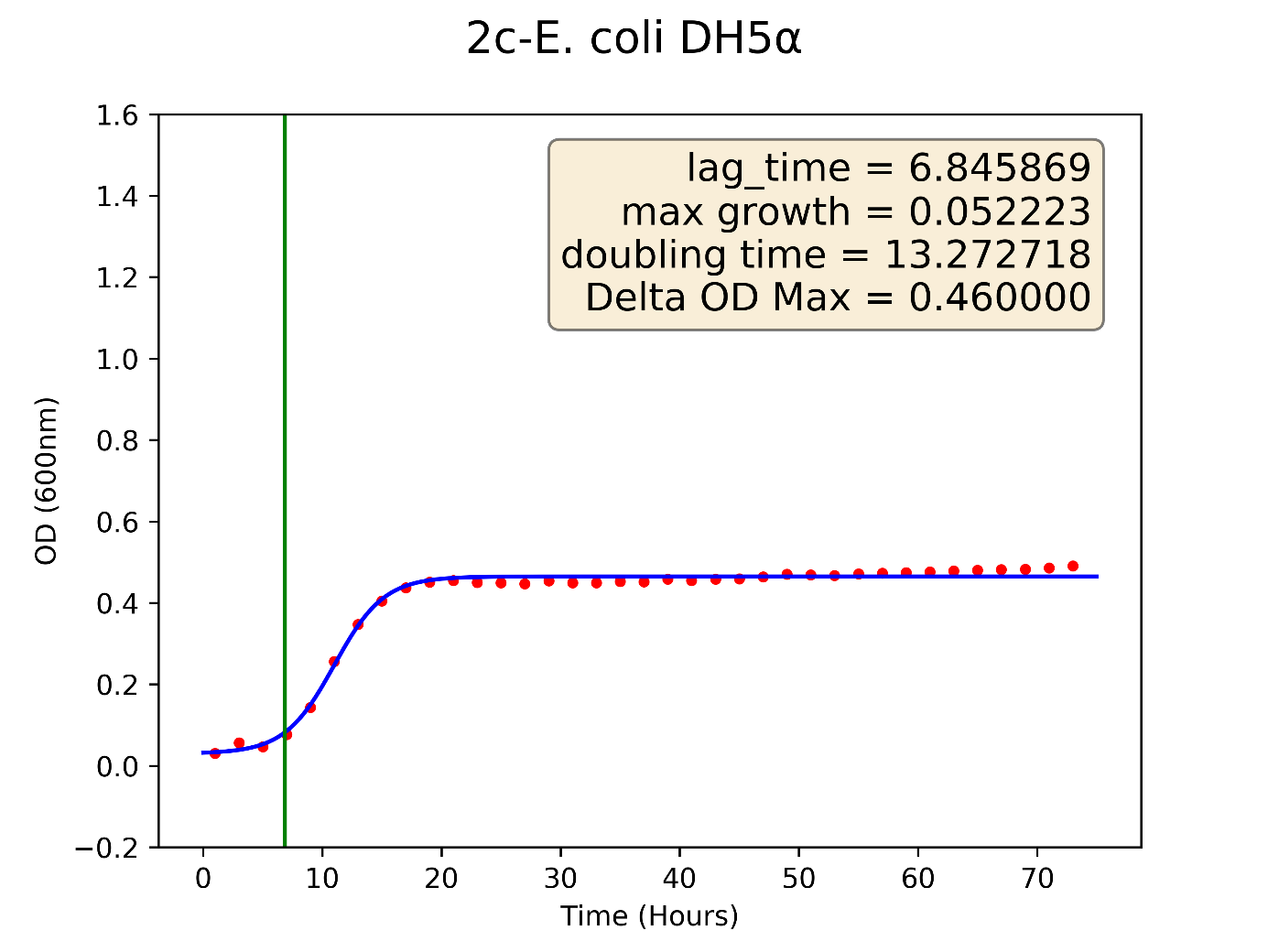


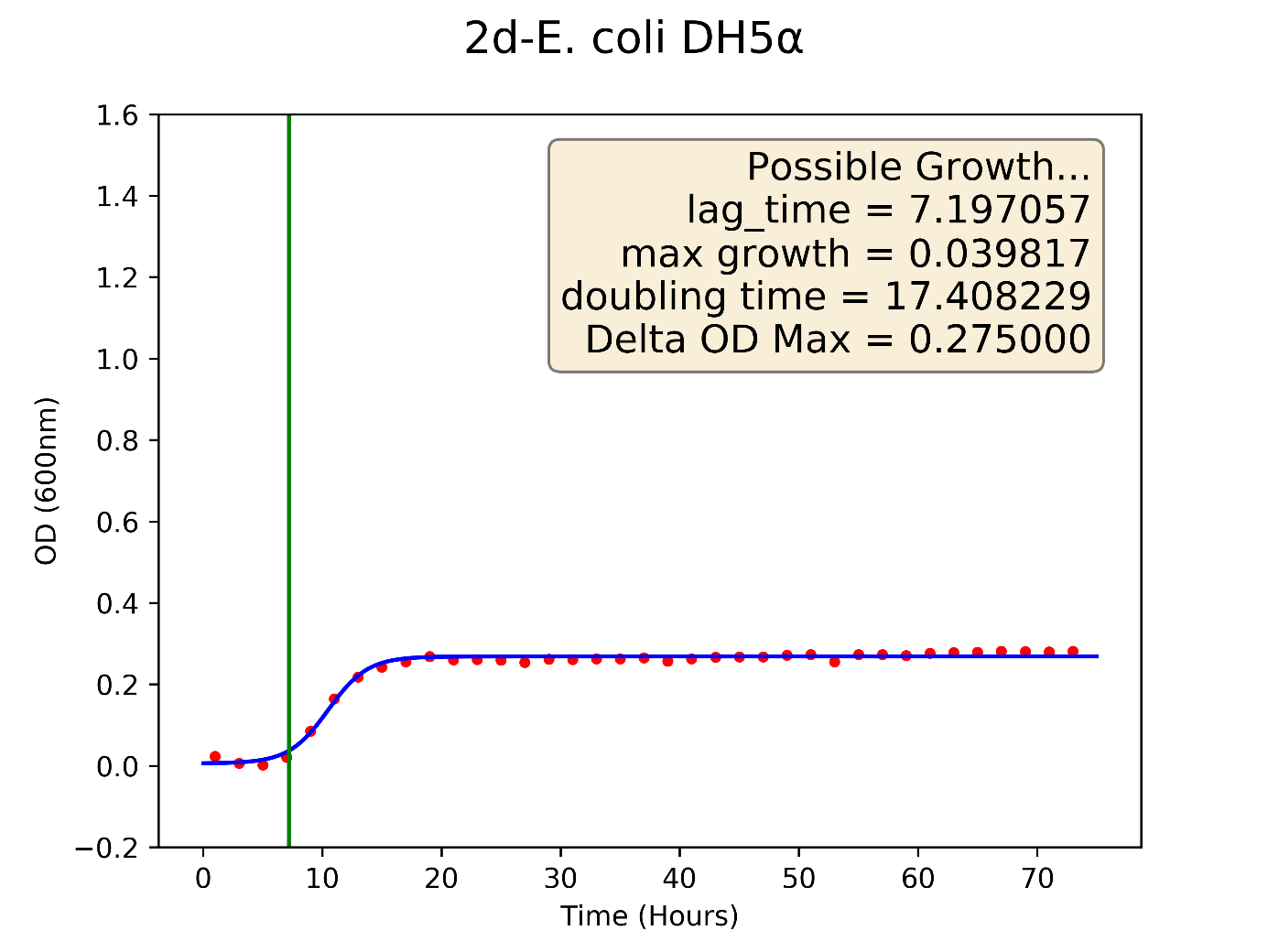


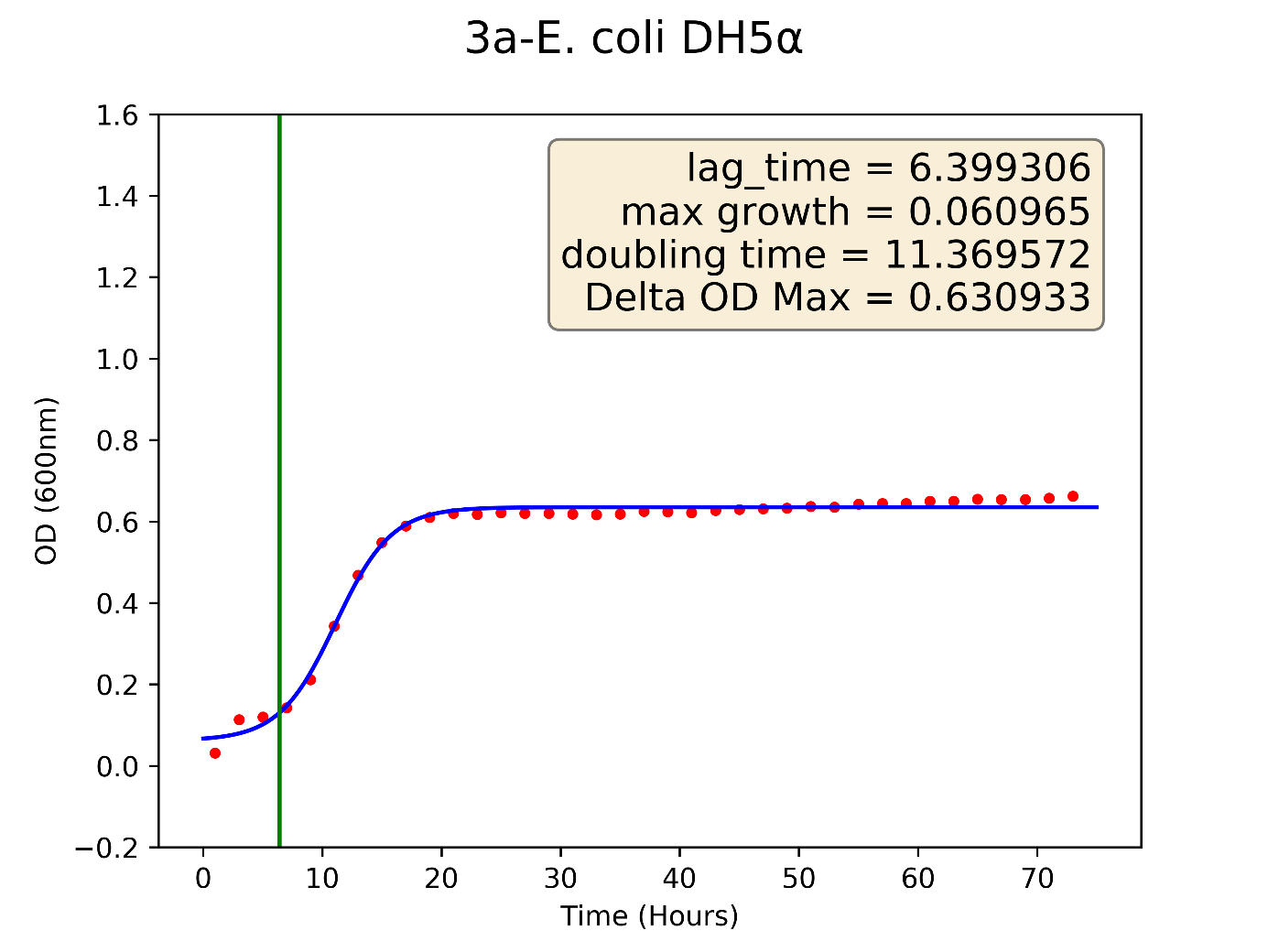


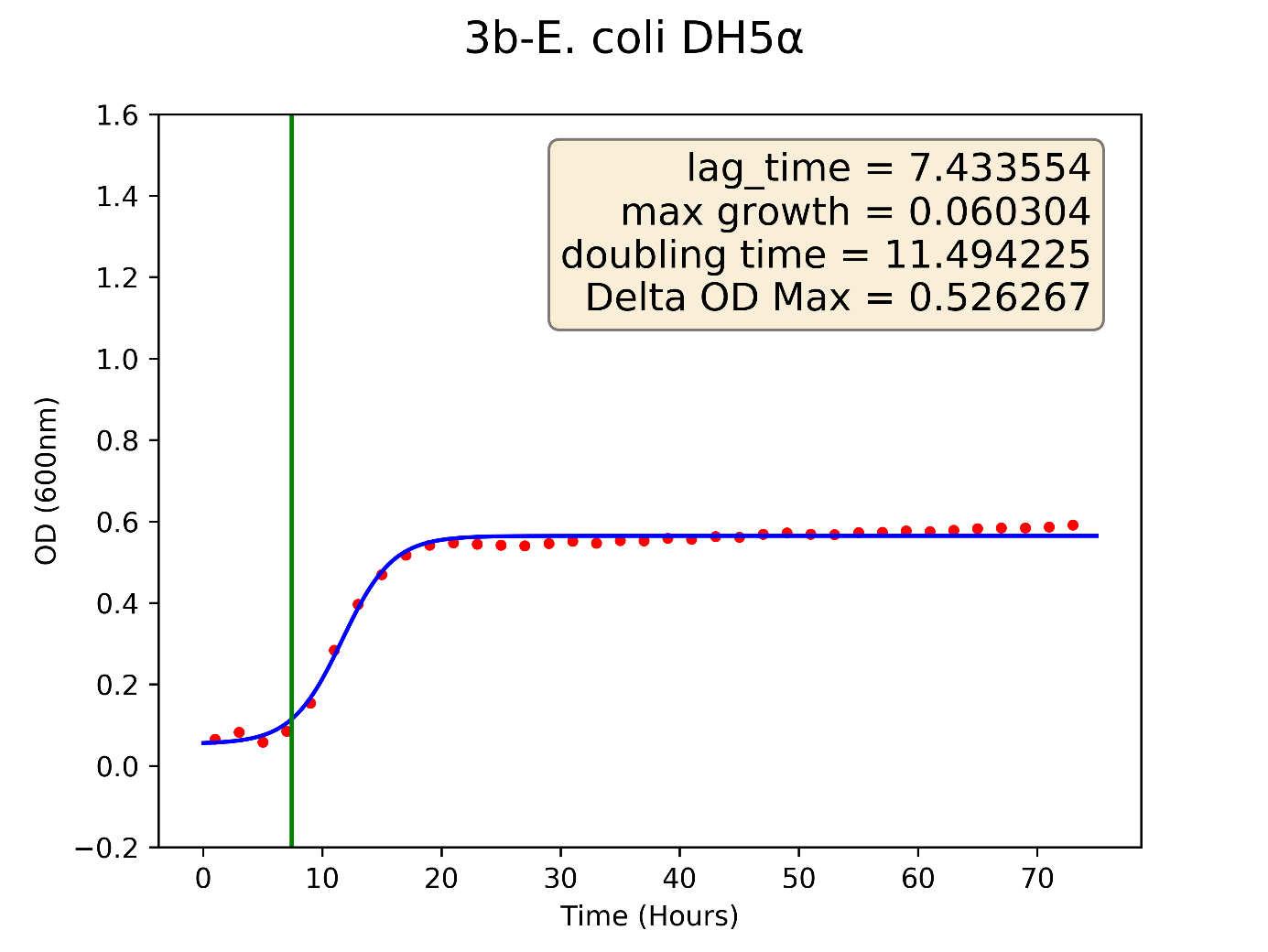


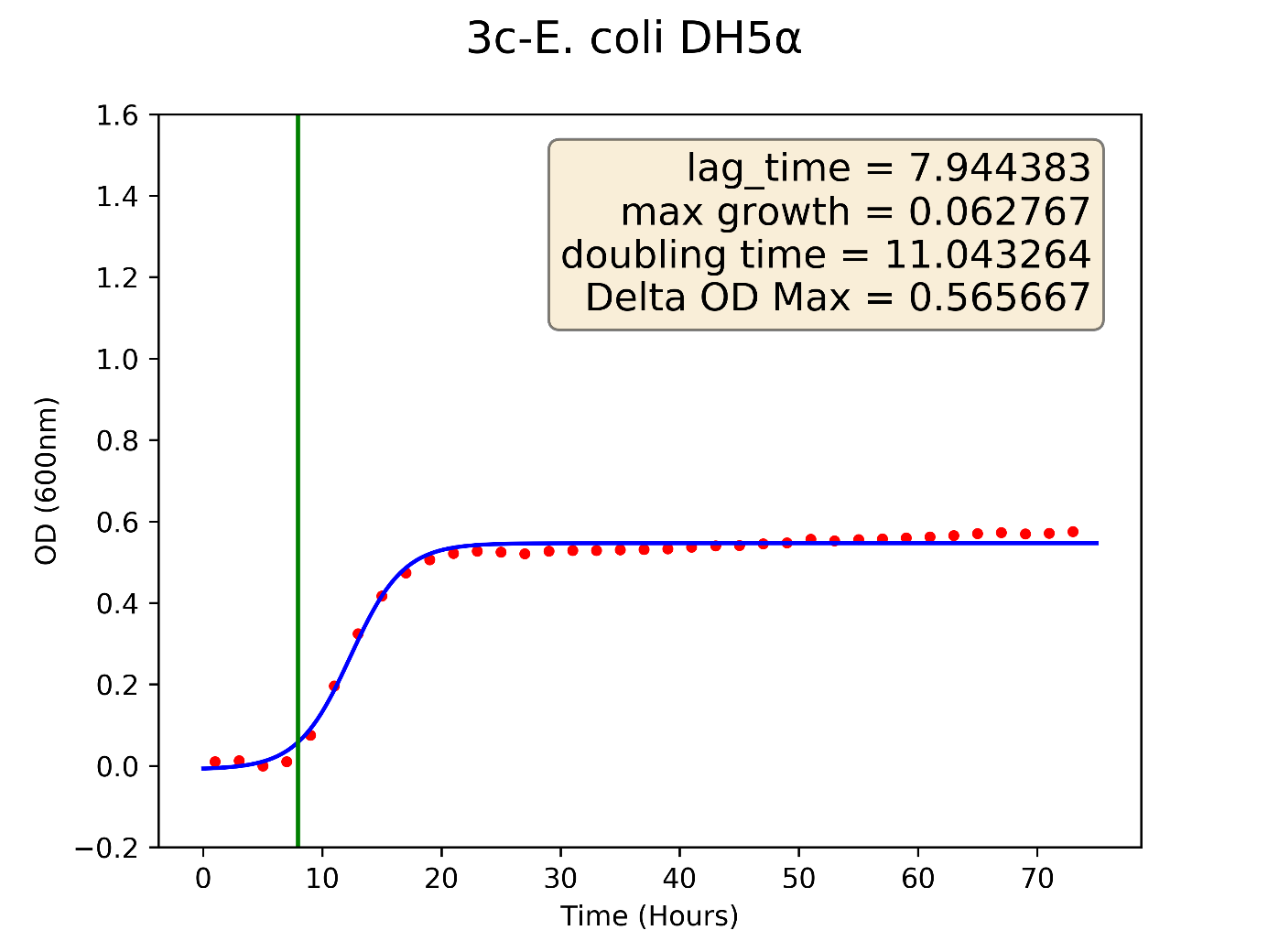


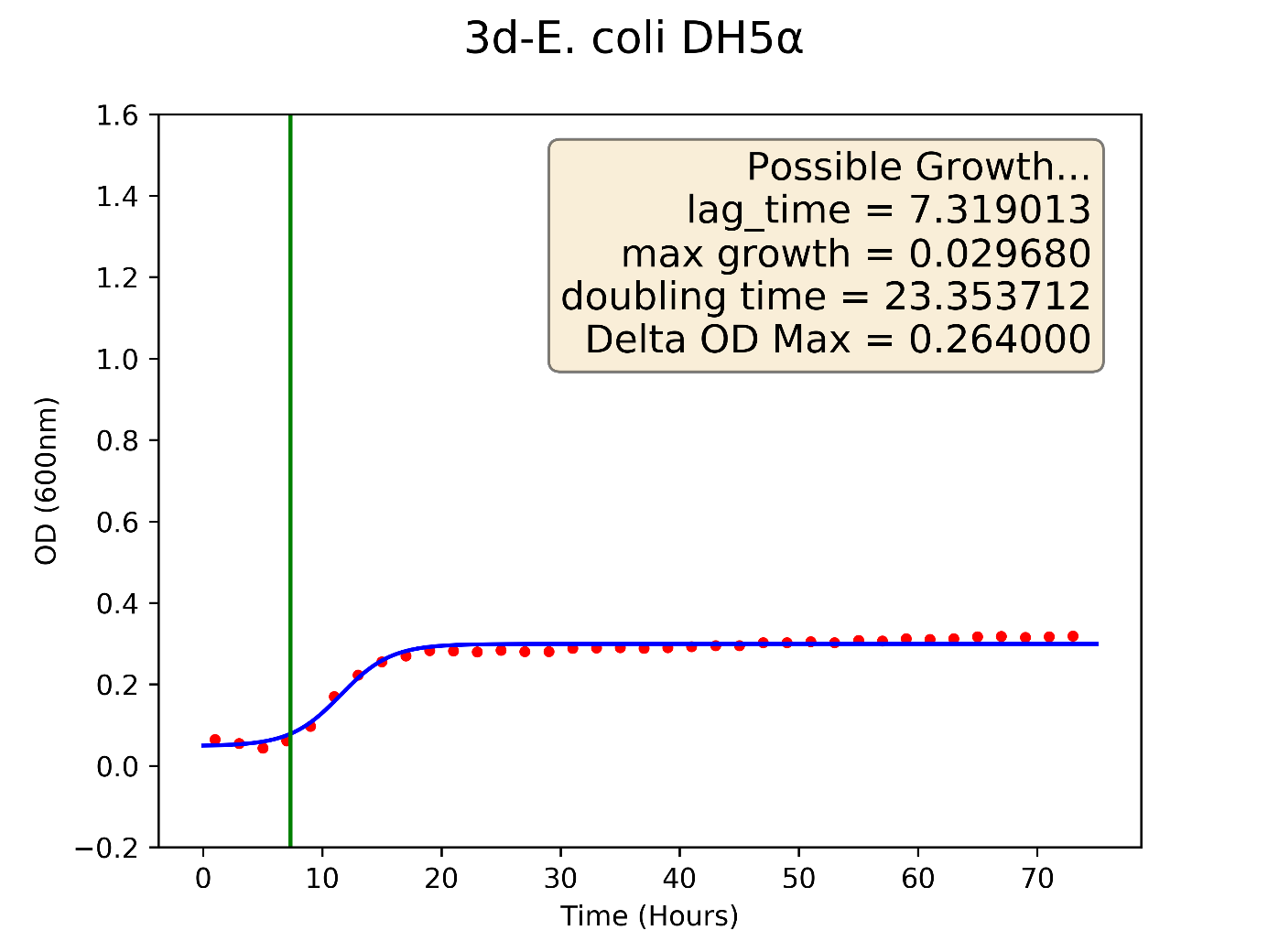


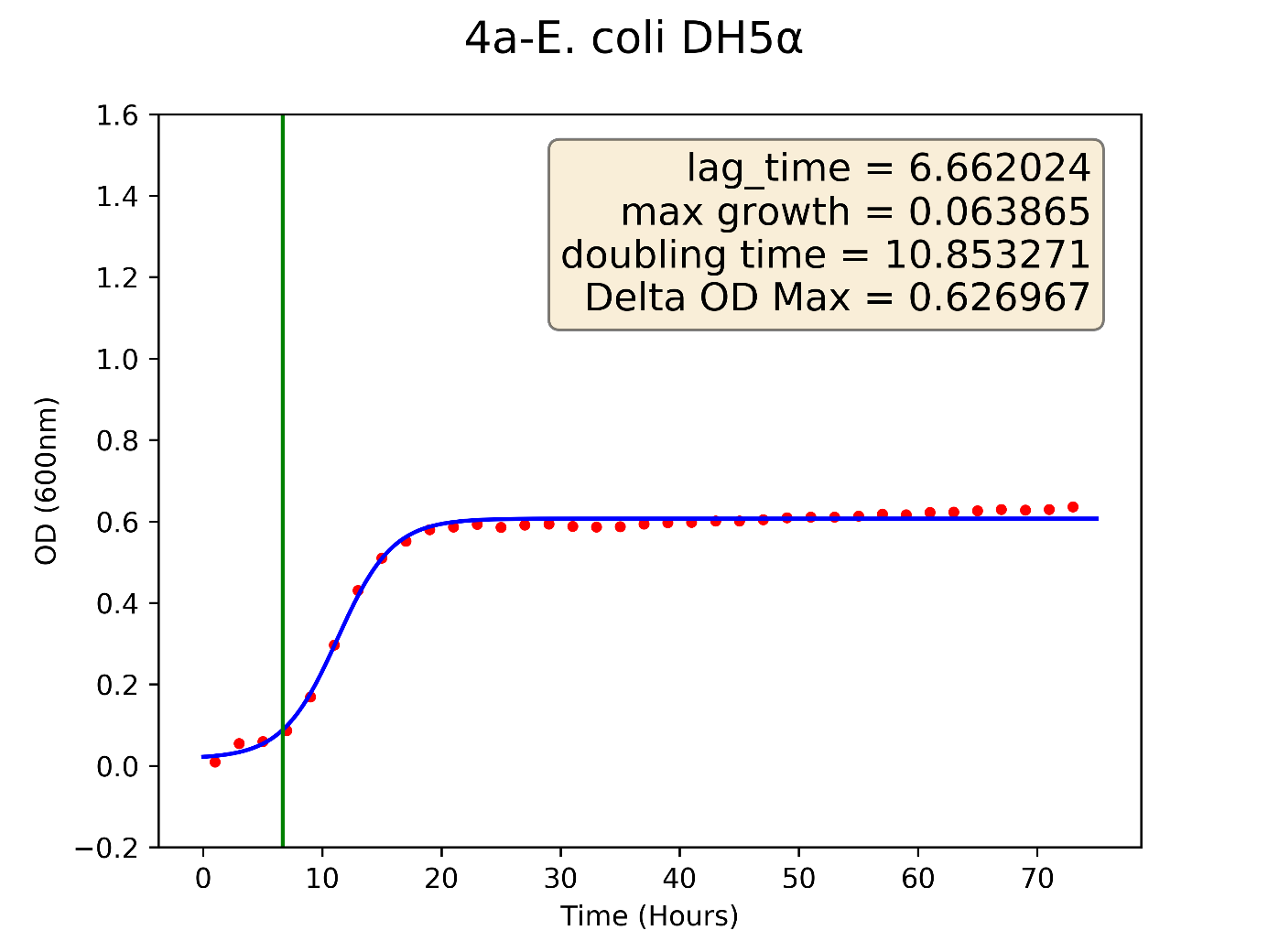


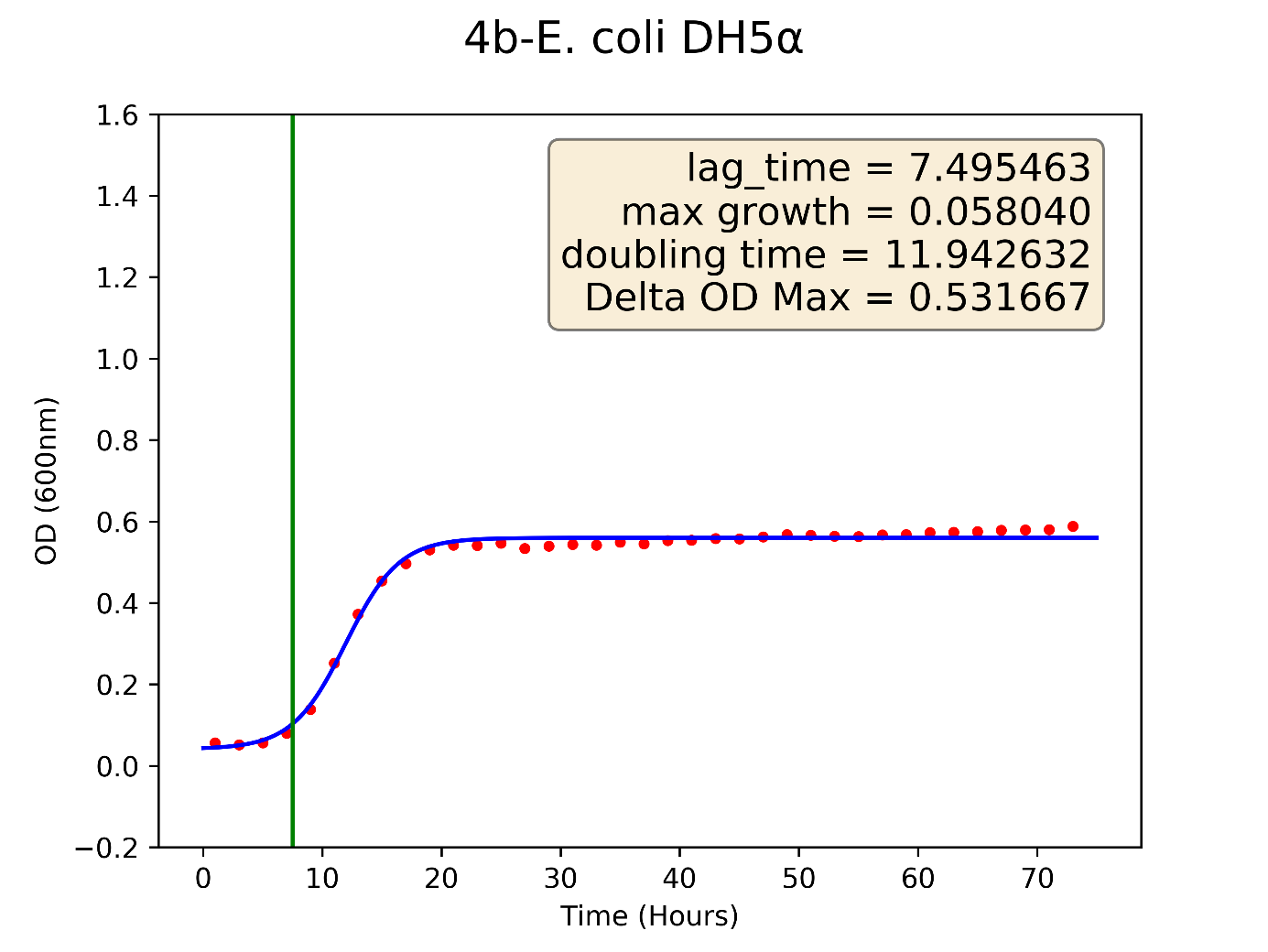


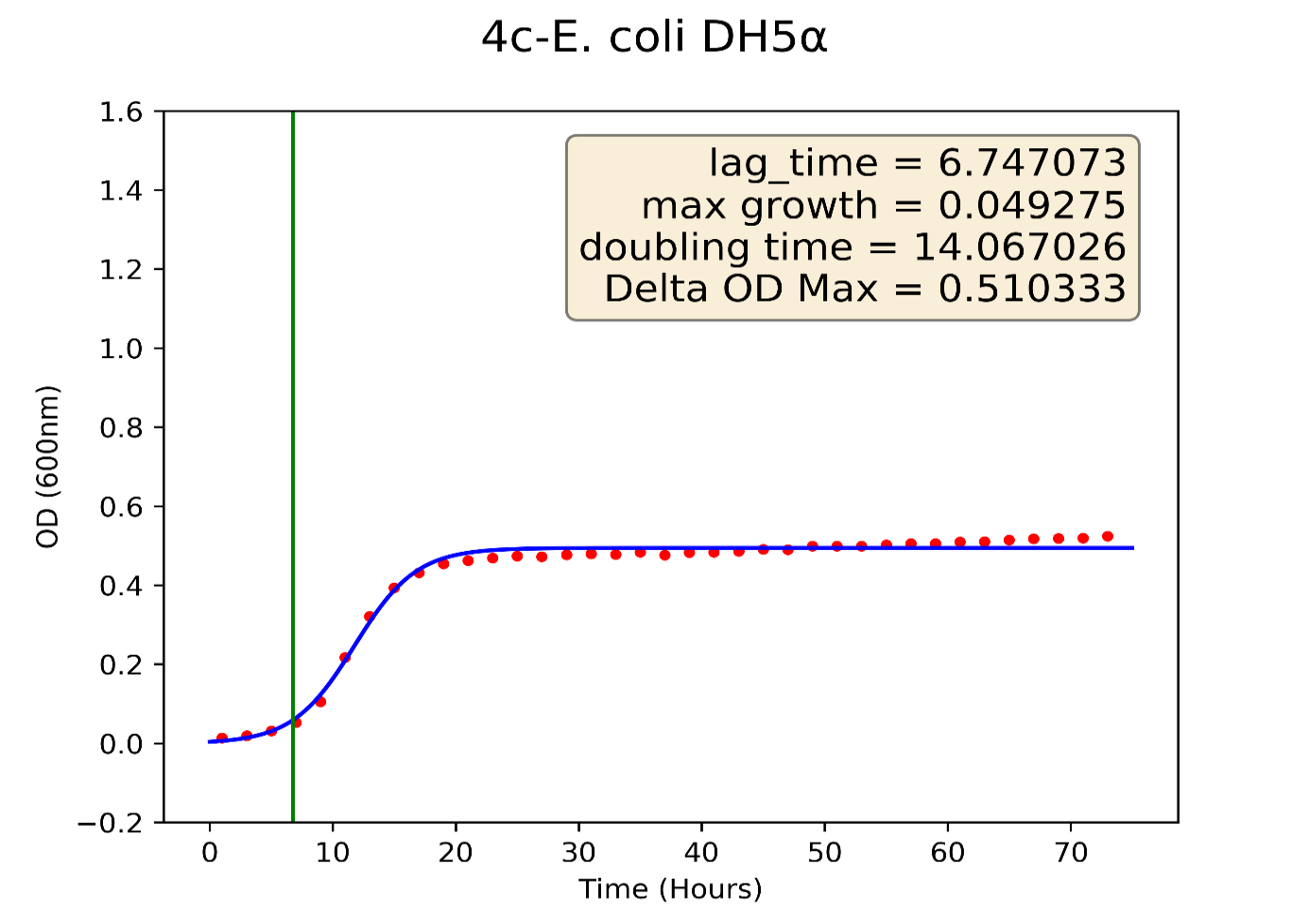


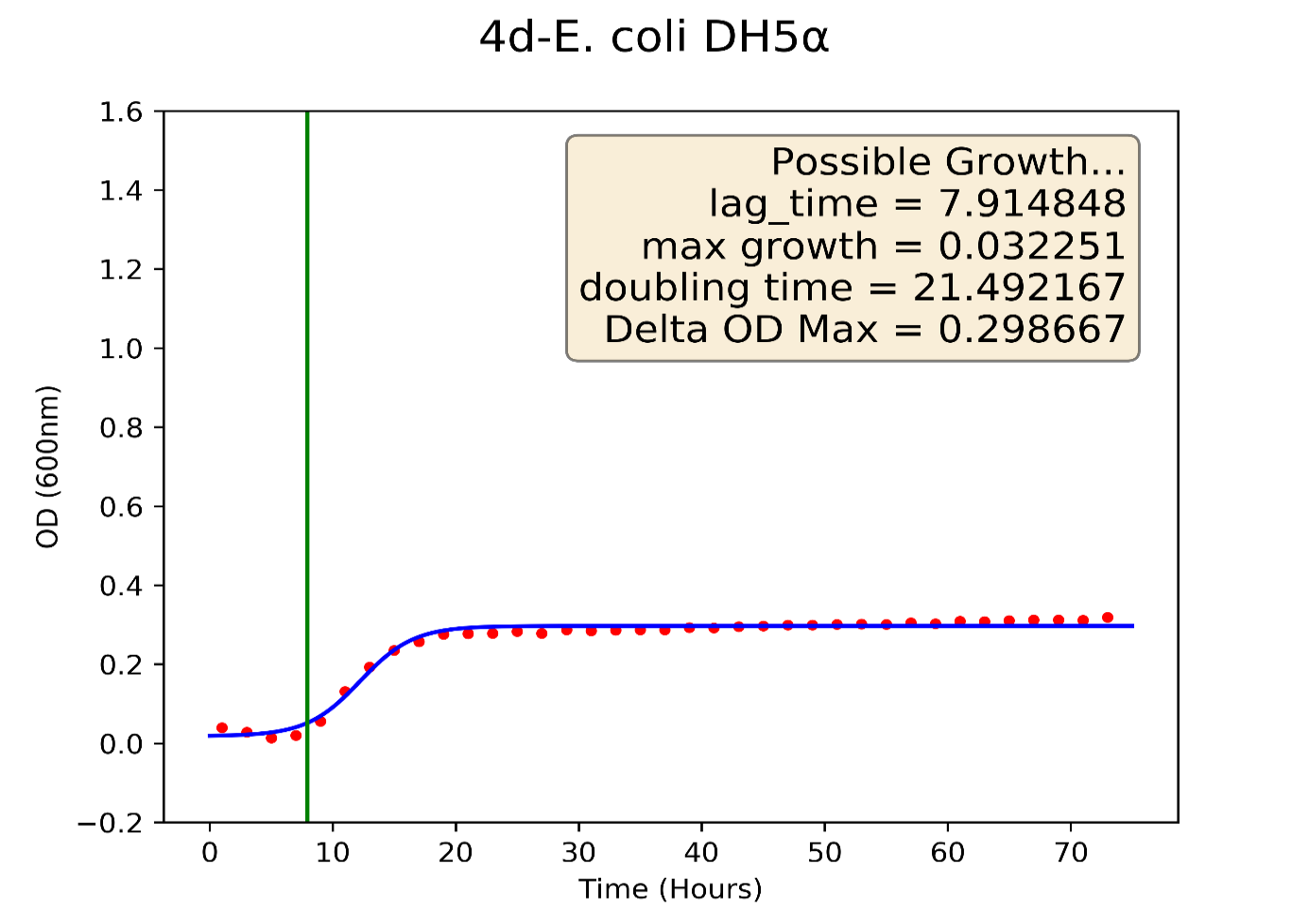


**Fig. S1.** Regression curves of selected data. *E. coli* (1, 2, 3, 4 – types of TiO2; a, b, c, d – concentration of TiO2 : 60, 150, 300 i 600 mg/L)

Supplement: Supplementary file 1 — (ZIP 8.20 MB) [file 12011_2021_2843_MOESM1_ESM.zip › 2S1 (E. coli)_ESM.docx]

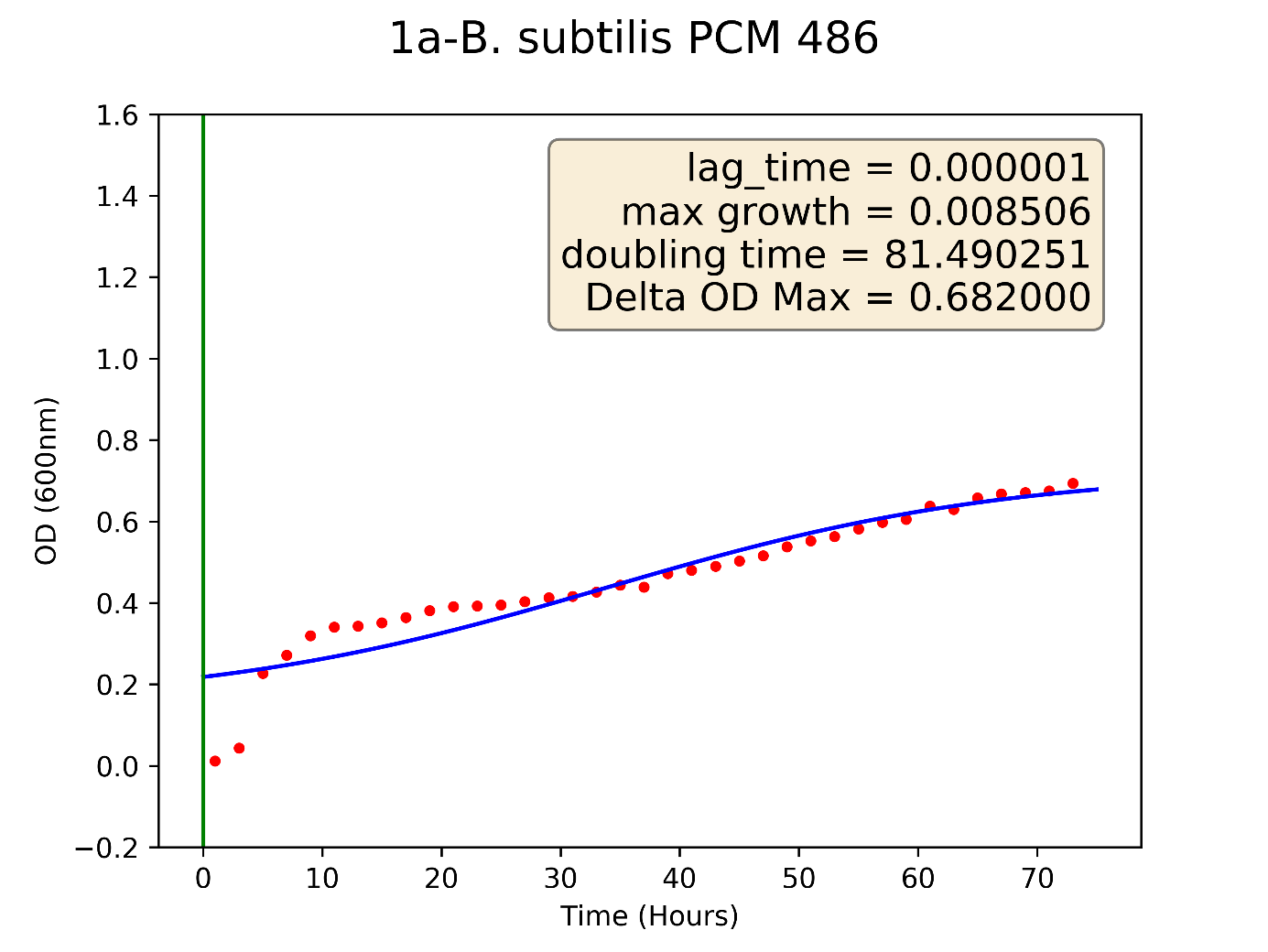


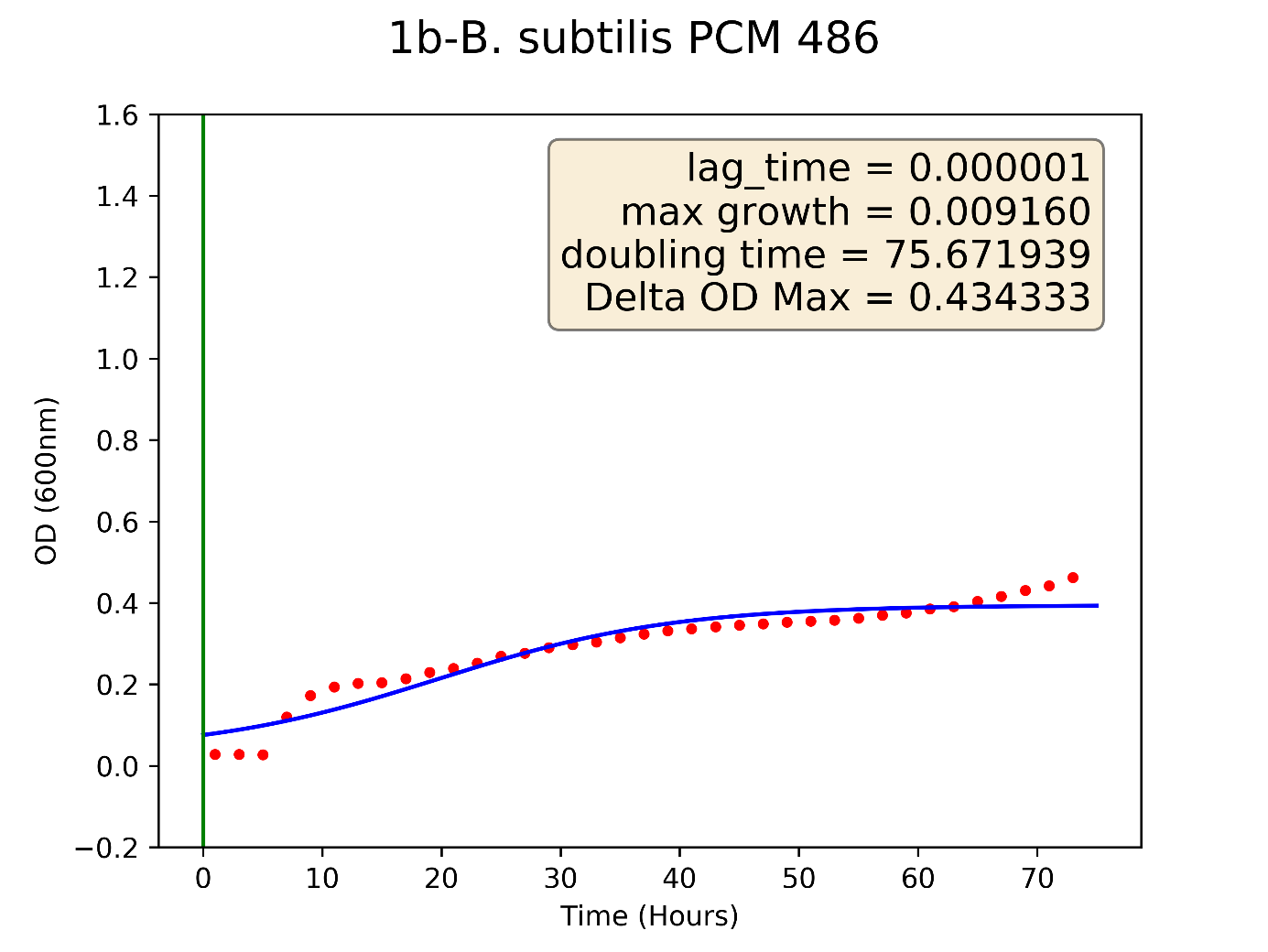


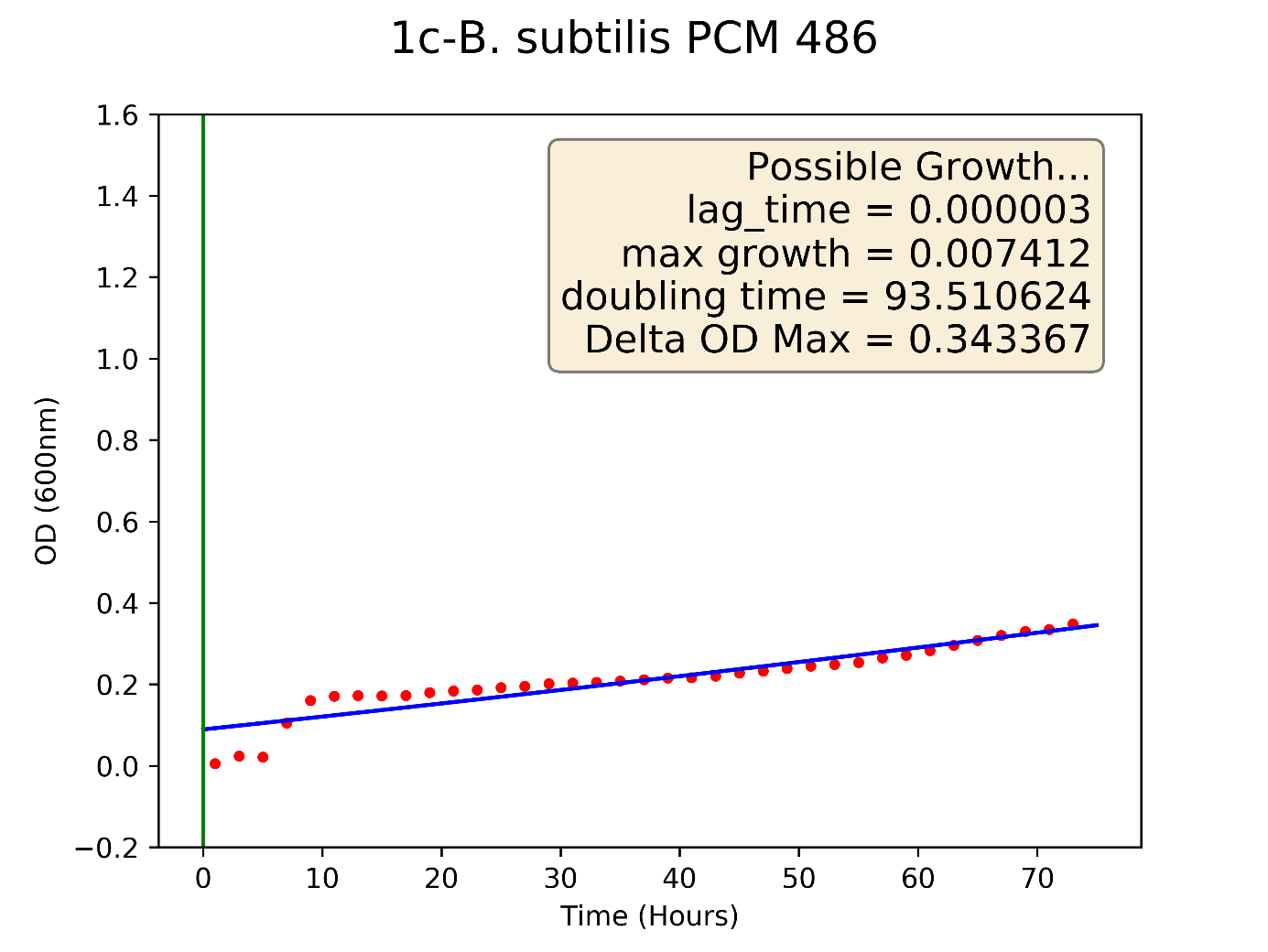


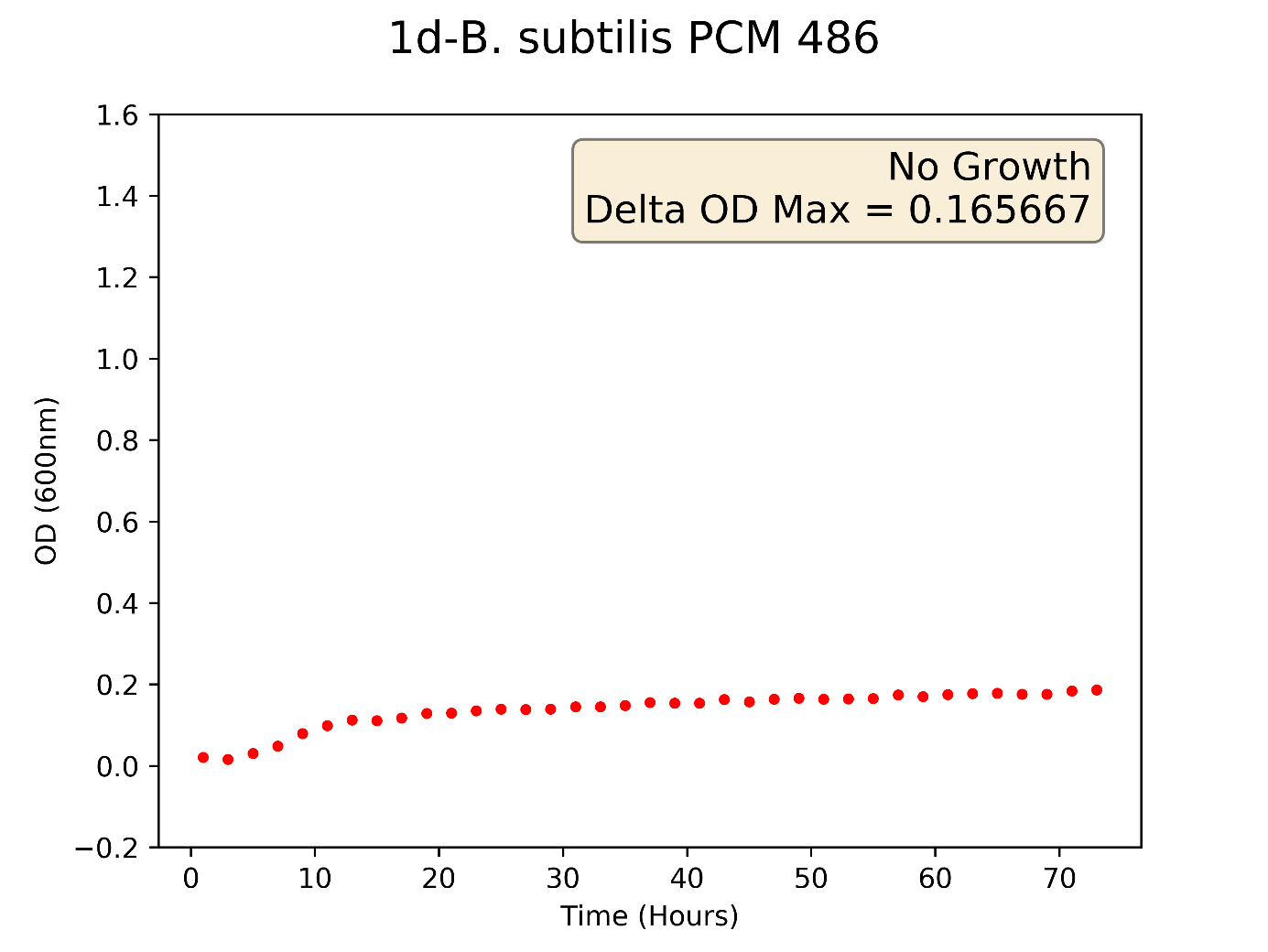


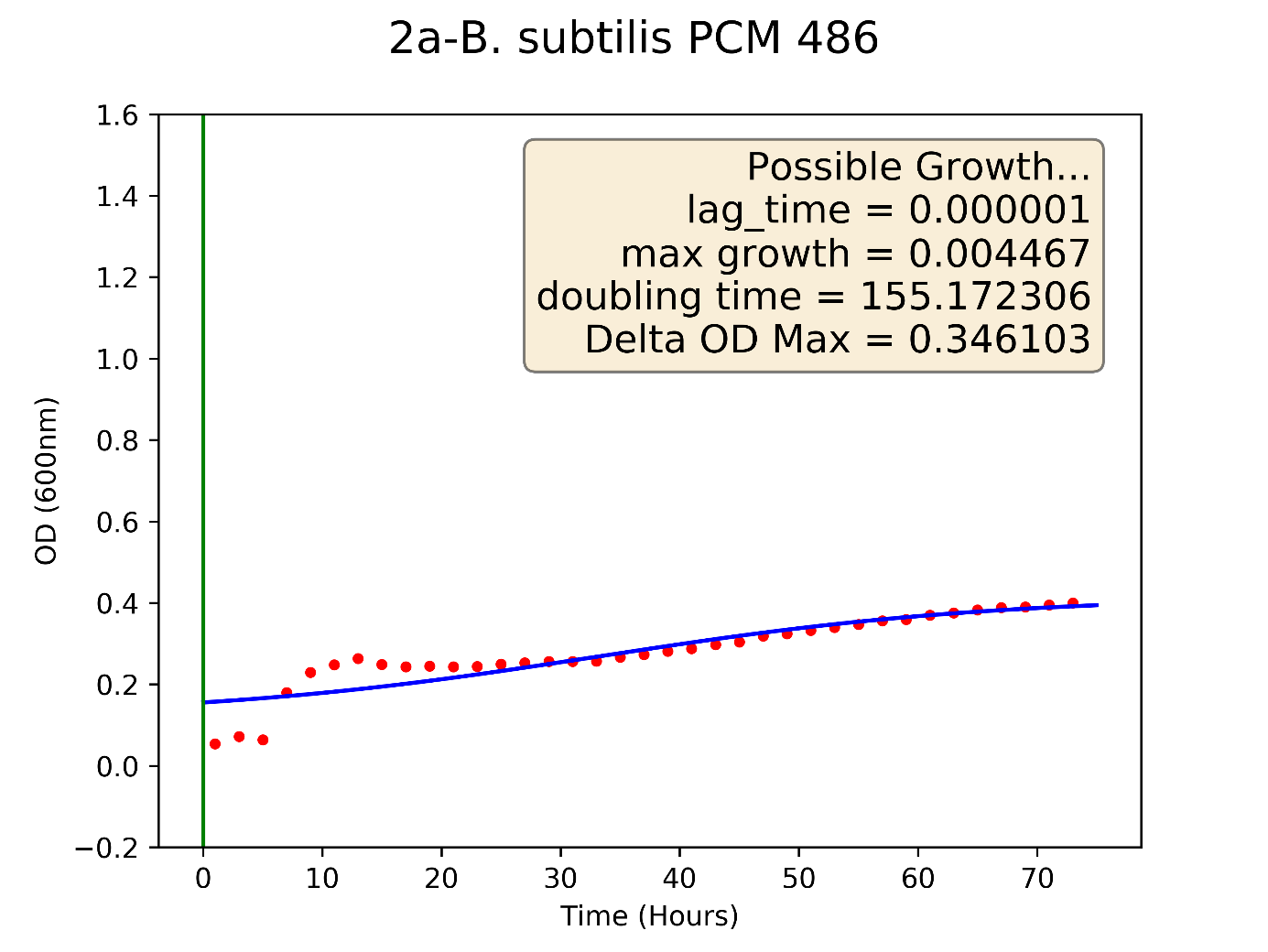


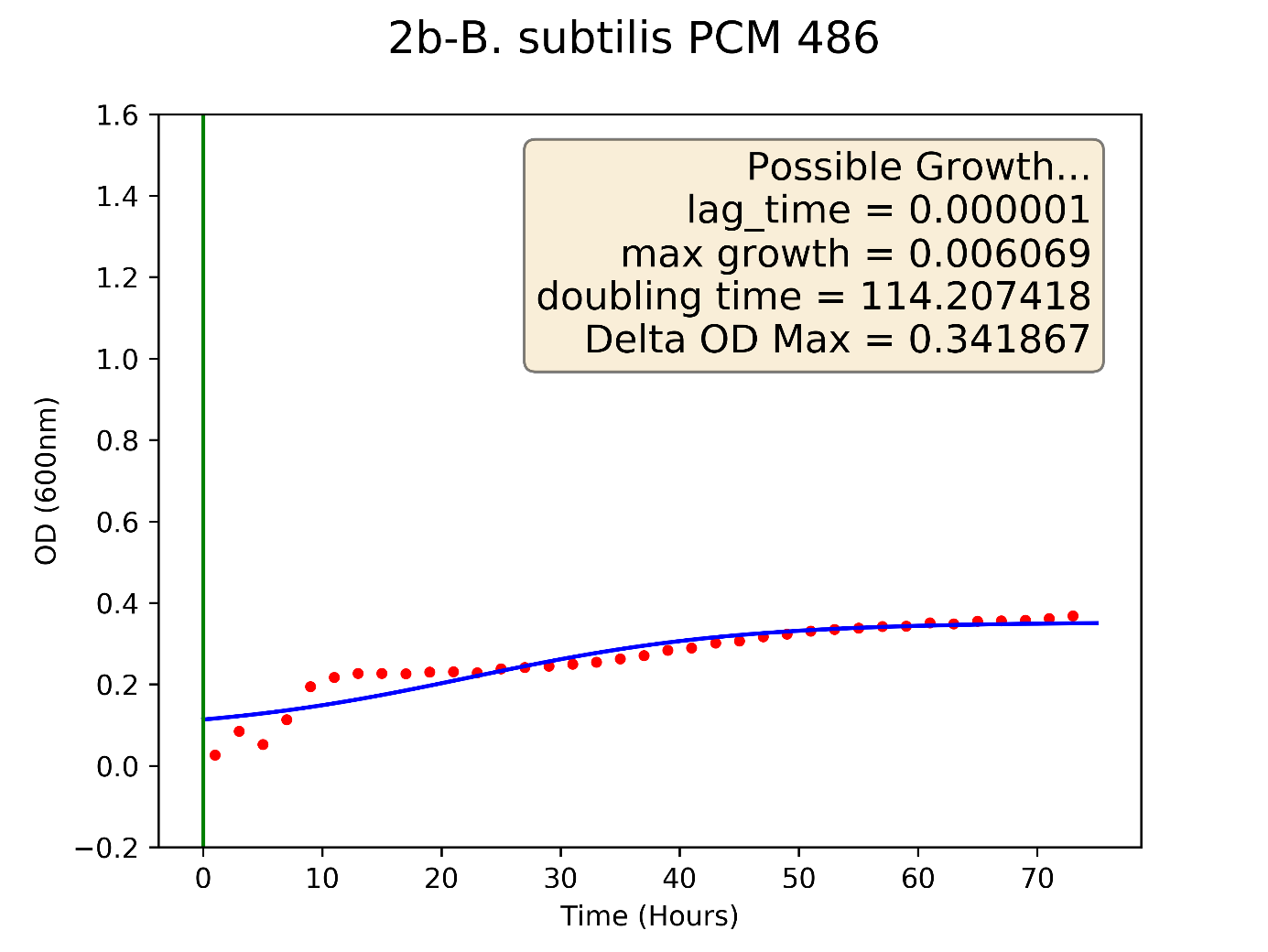


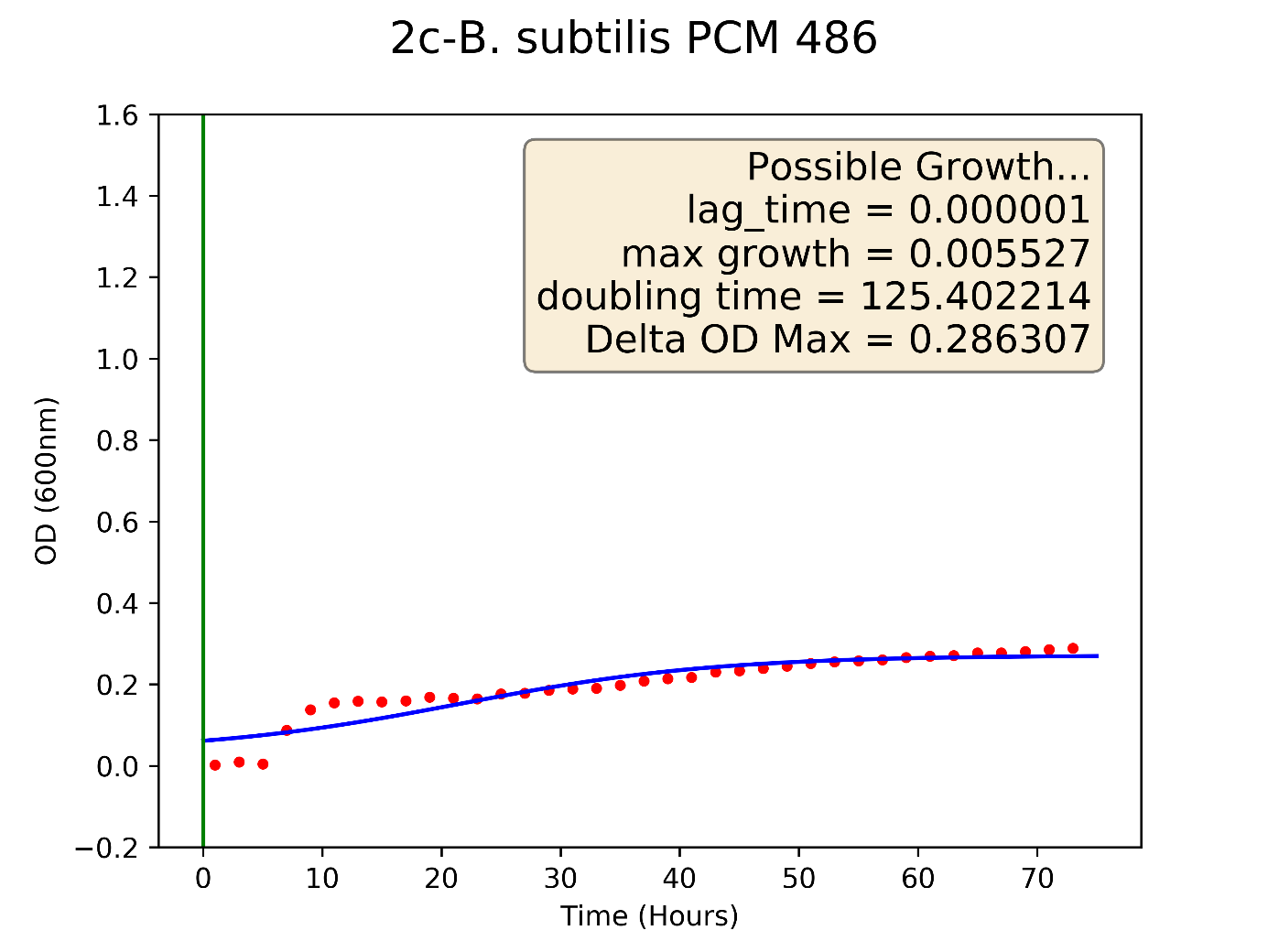


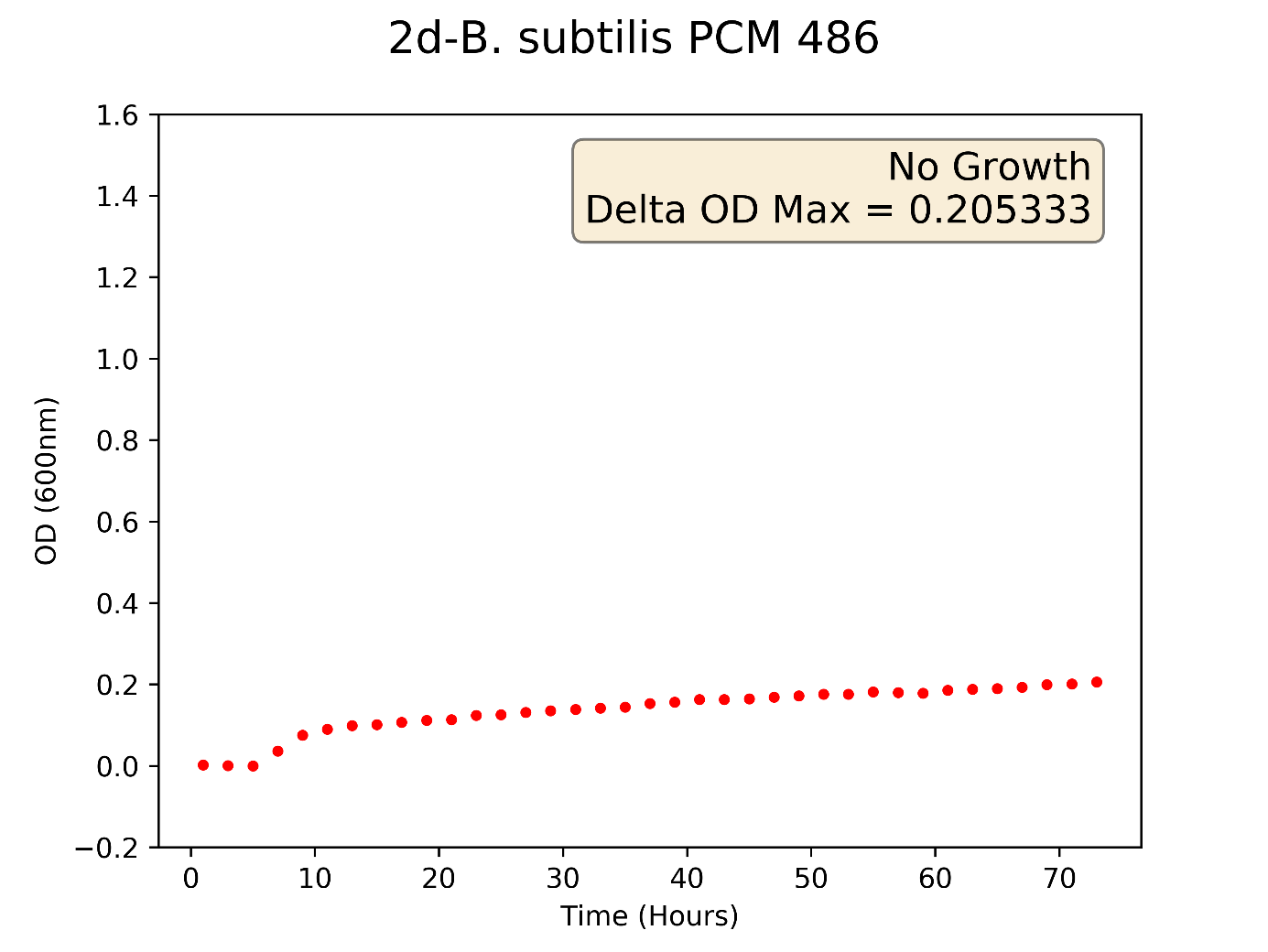


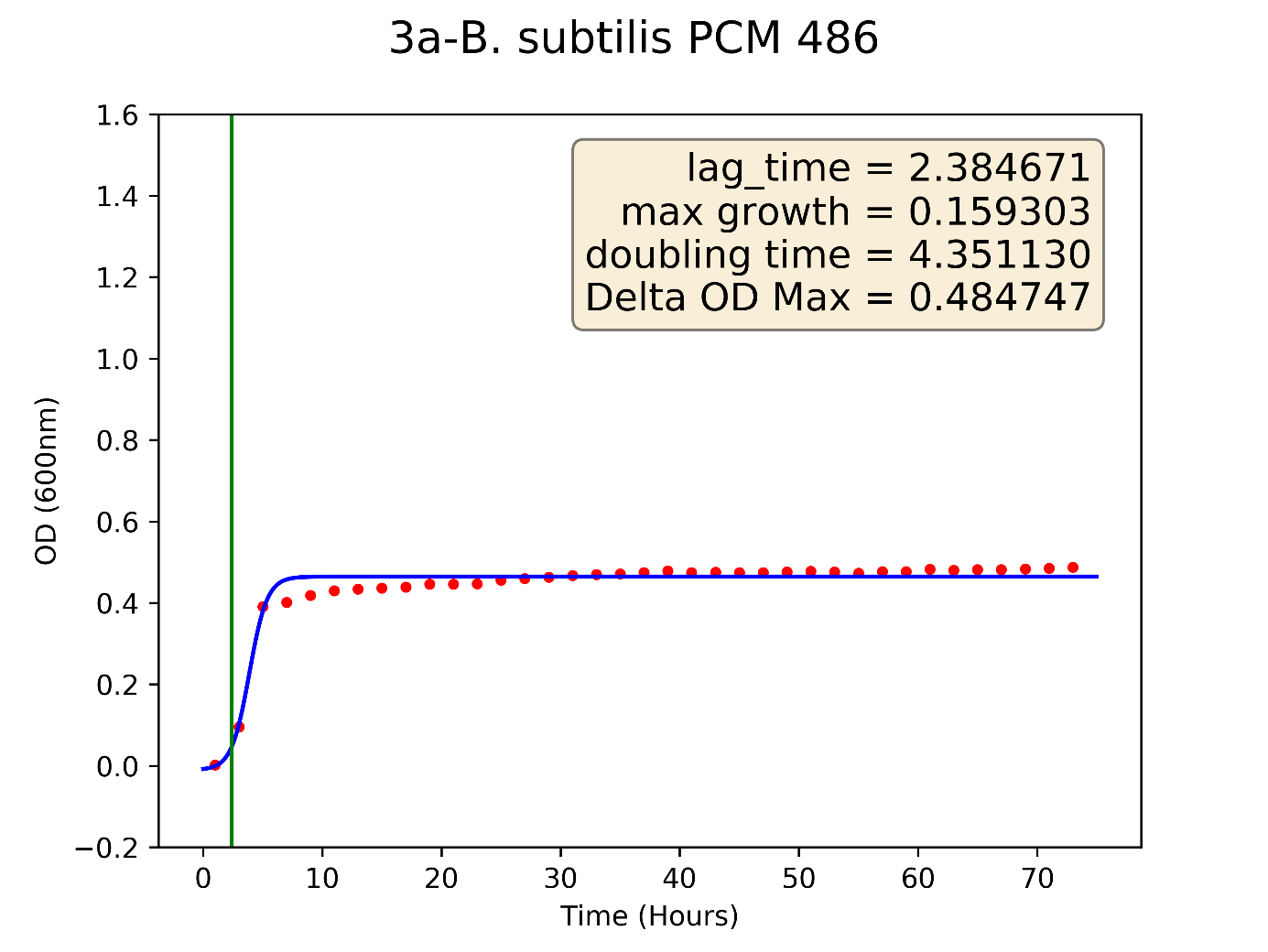


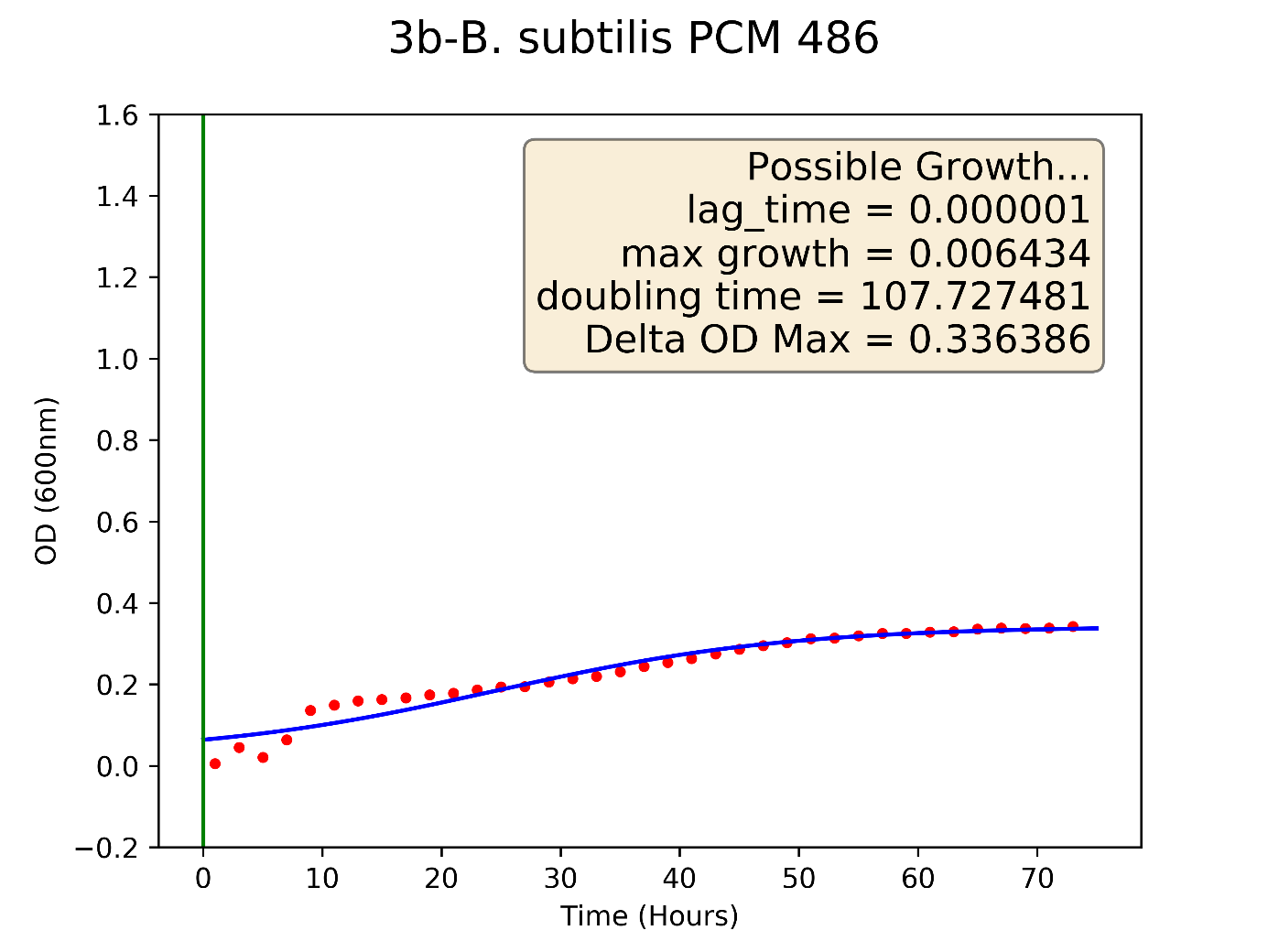


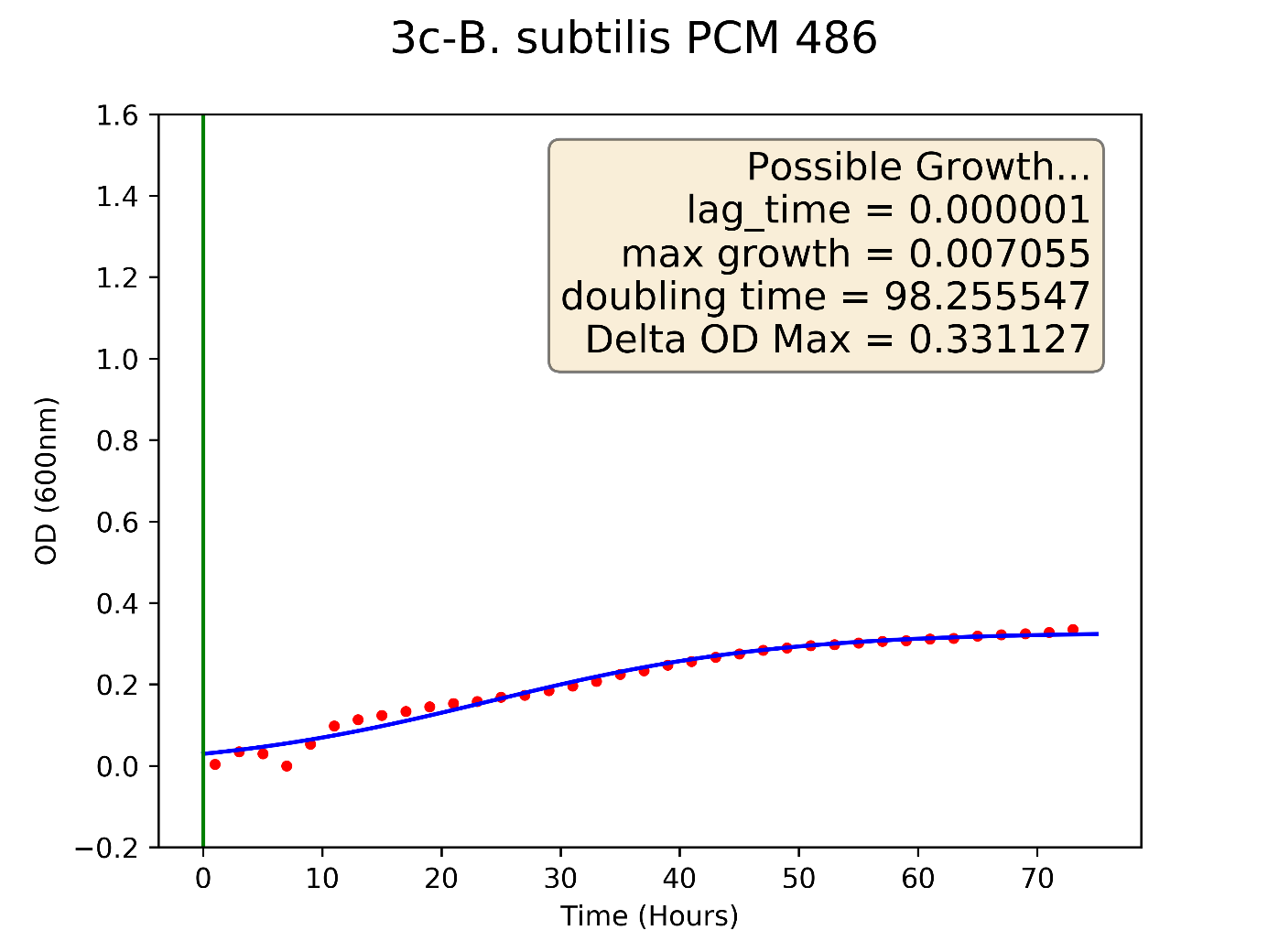


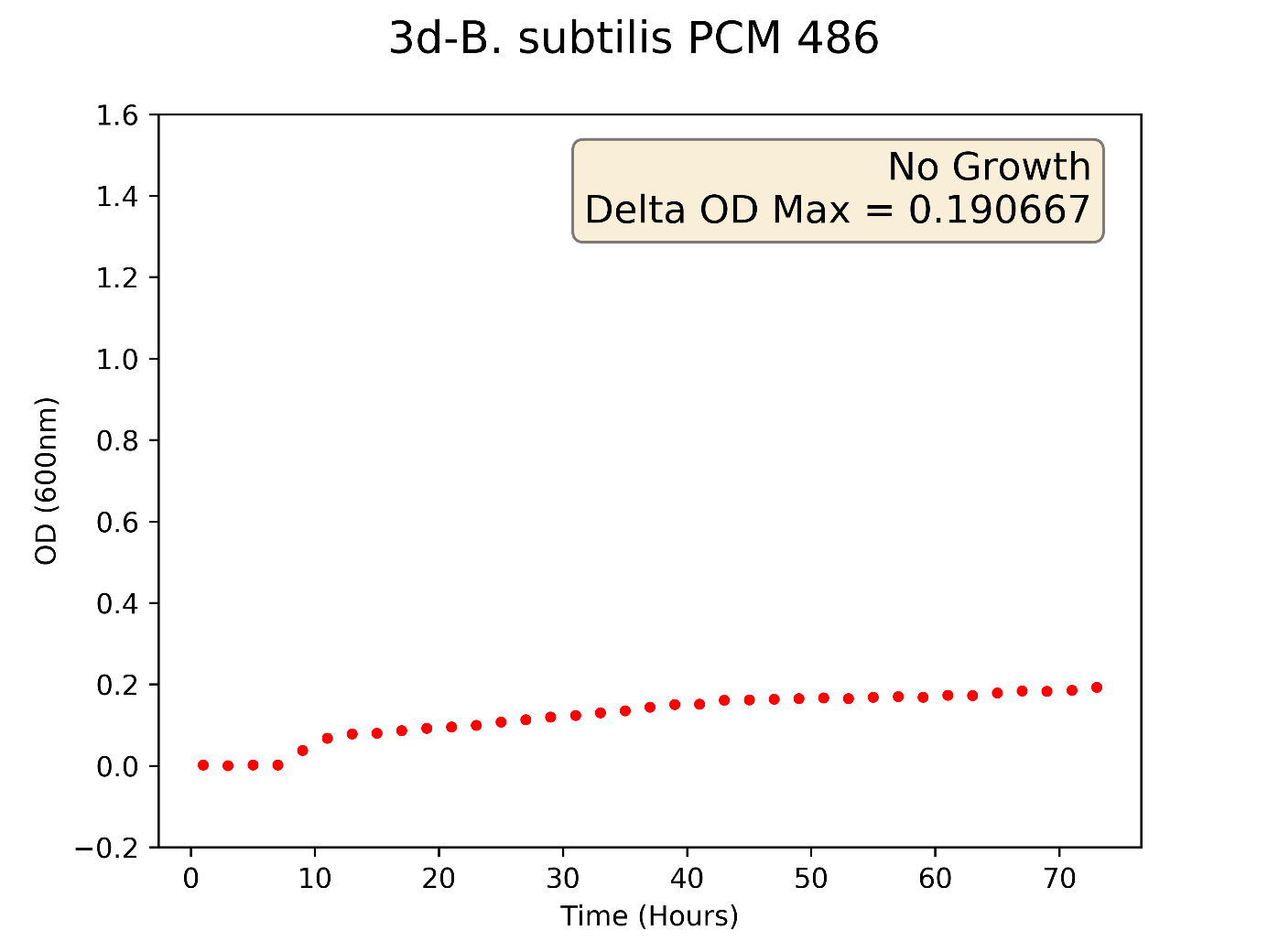


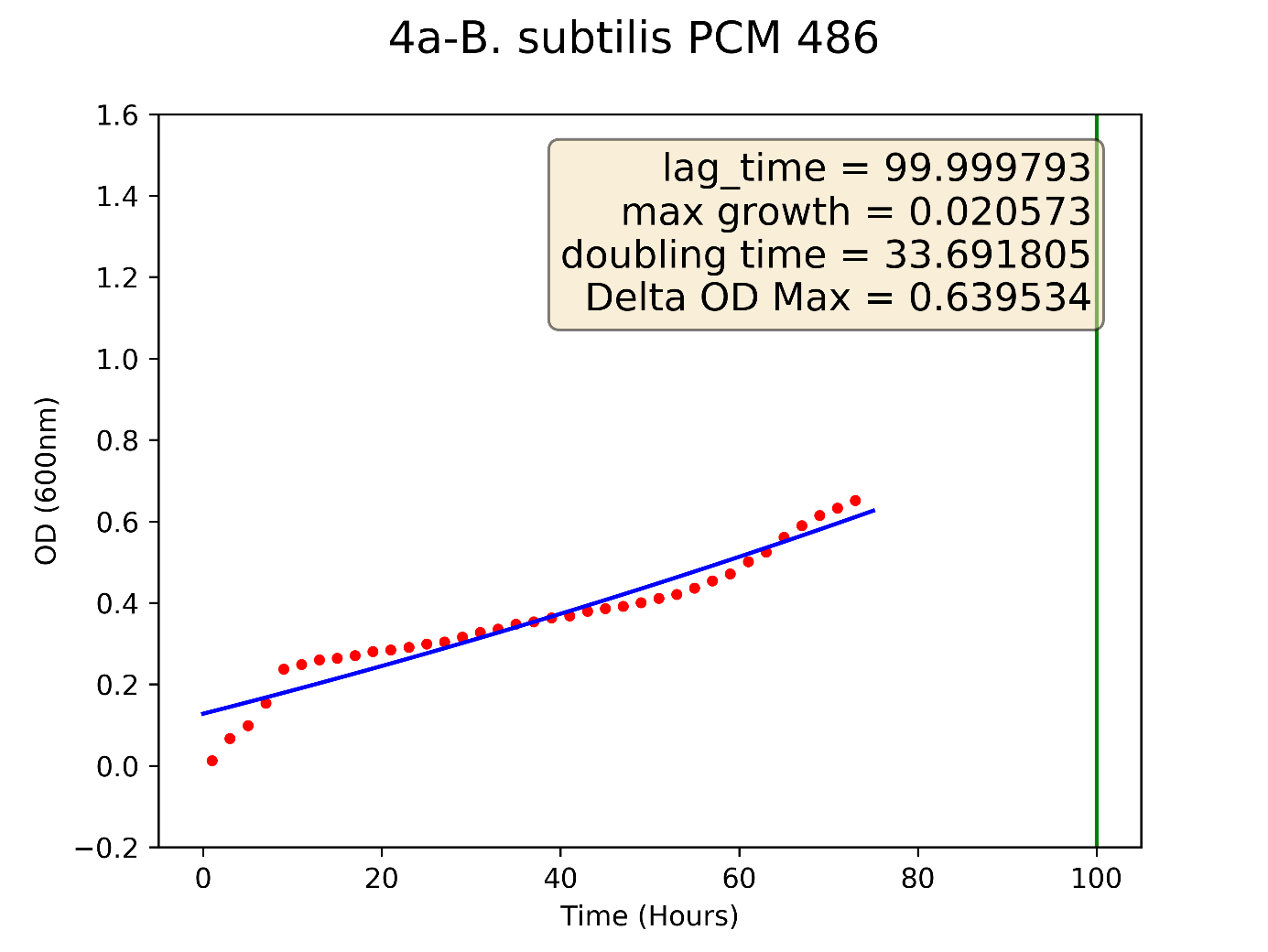


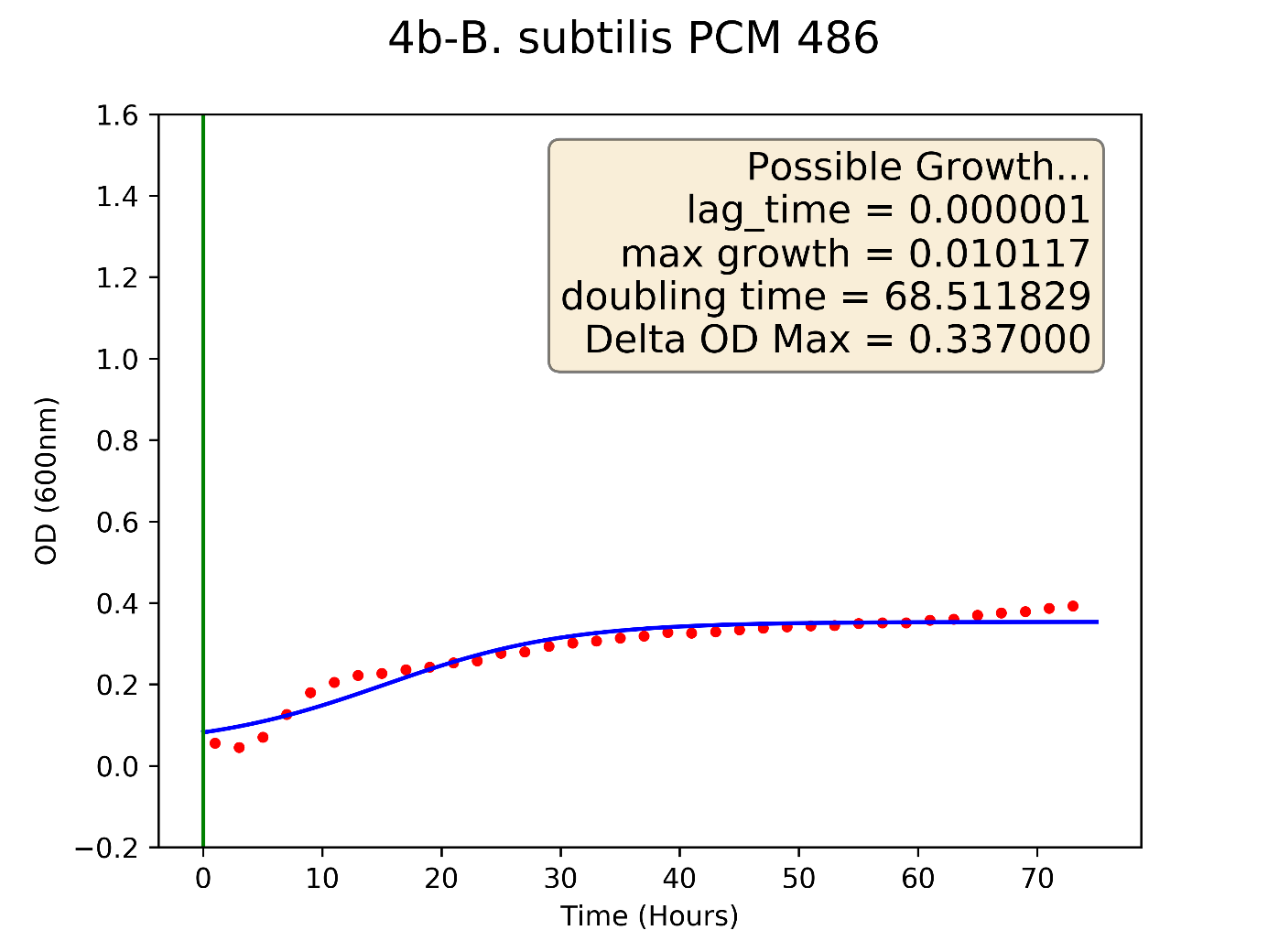


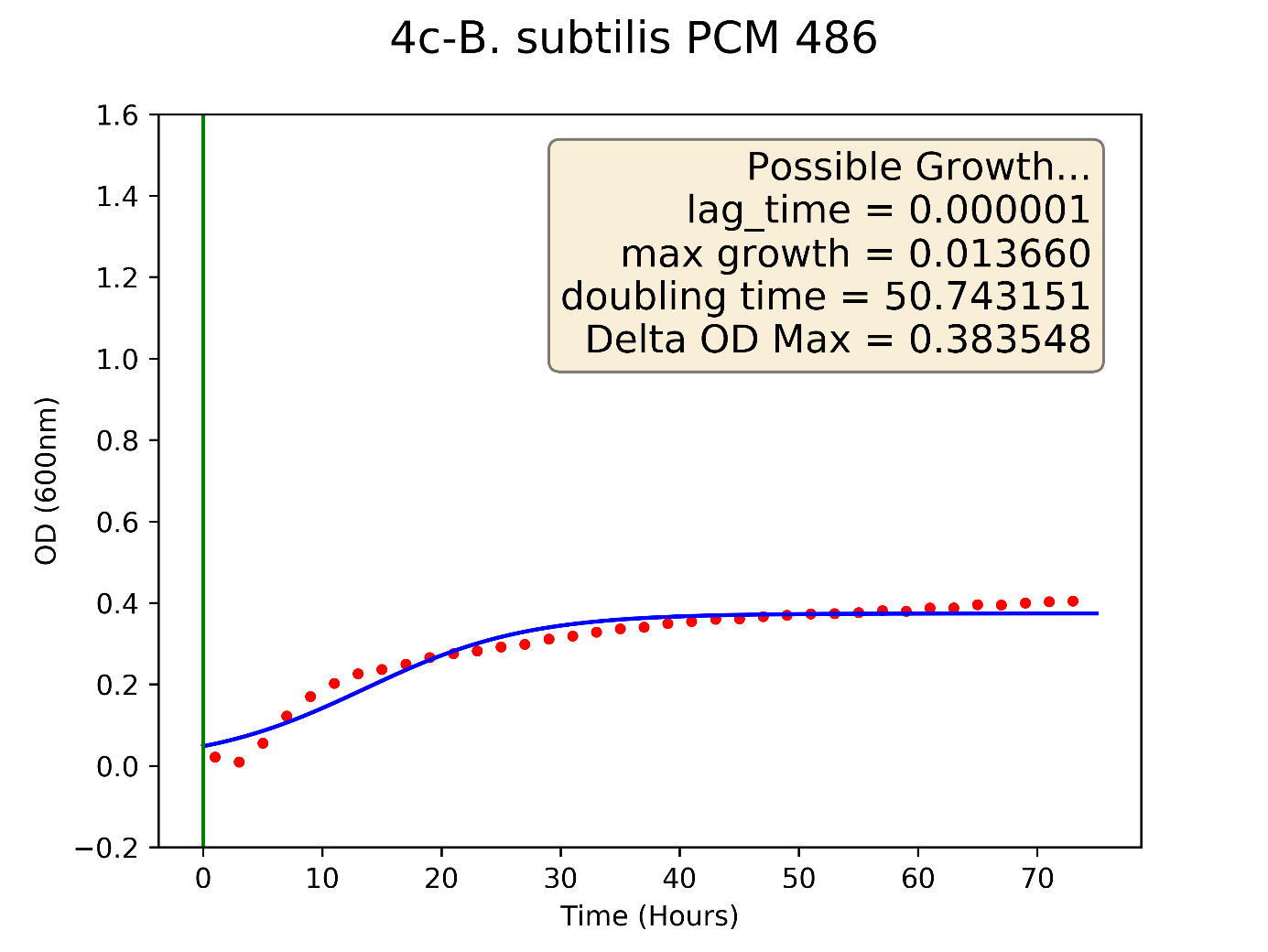


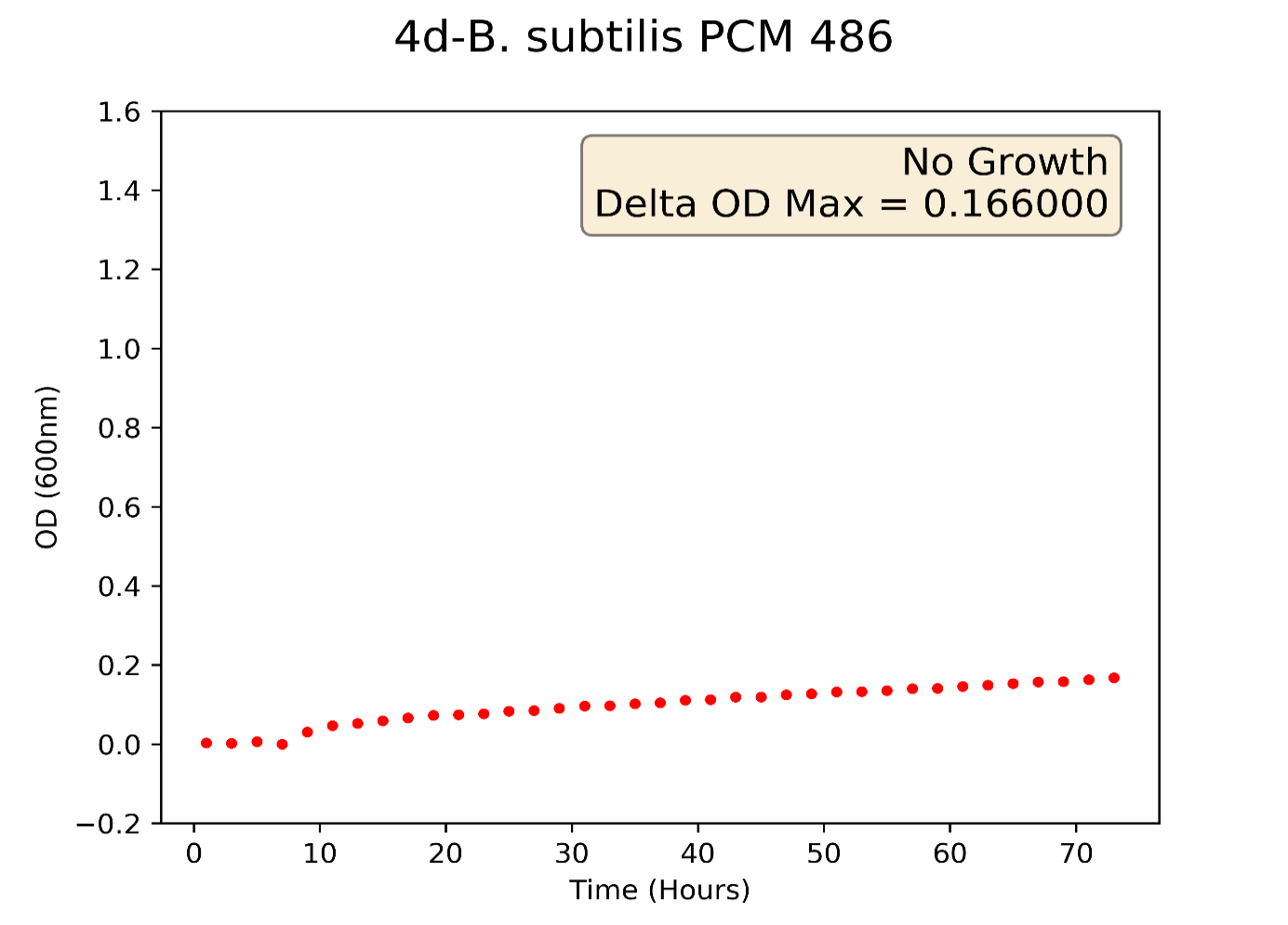


**Fig. S1.** Regression curves of selected data. *B. subtilis* (1, 2, 3, 4 – types of TiO2; a, b, c, d – concentration of TiO2 : 60, 150, 300 i 600 mg/L)

Supplement: Supplementary file 1 — (ZIP 8.20 MB) [file 12011_2021_2843_MOESM1_ESM.zip › 2S2 (B. subtillis)_ESM.docx]

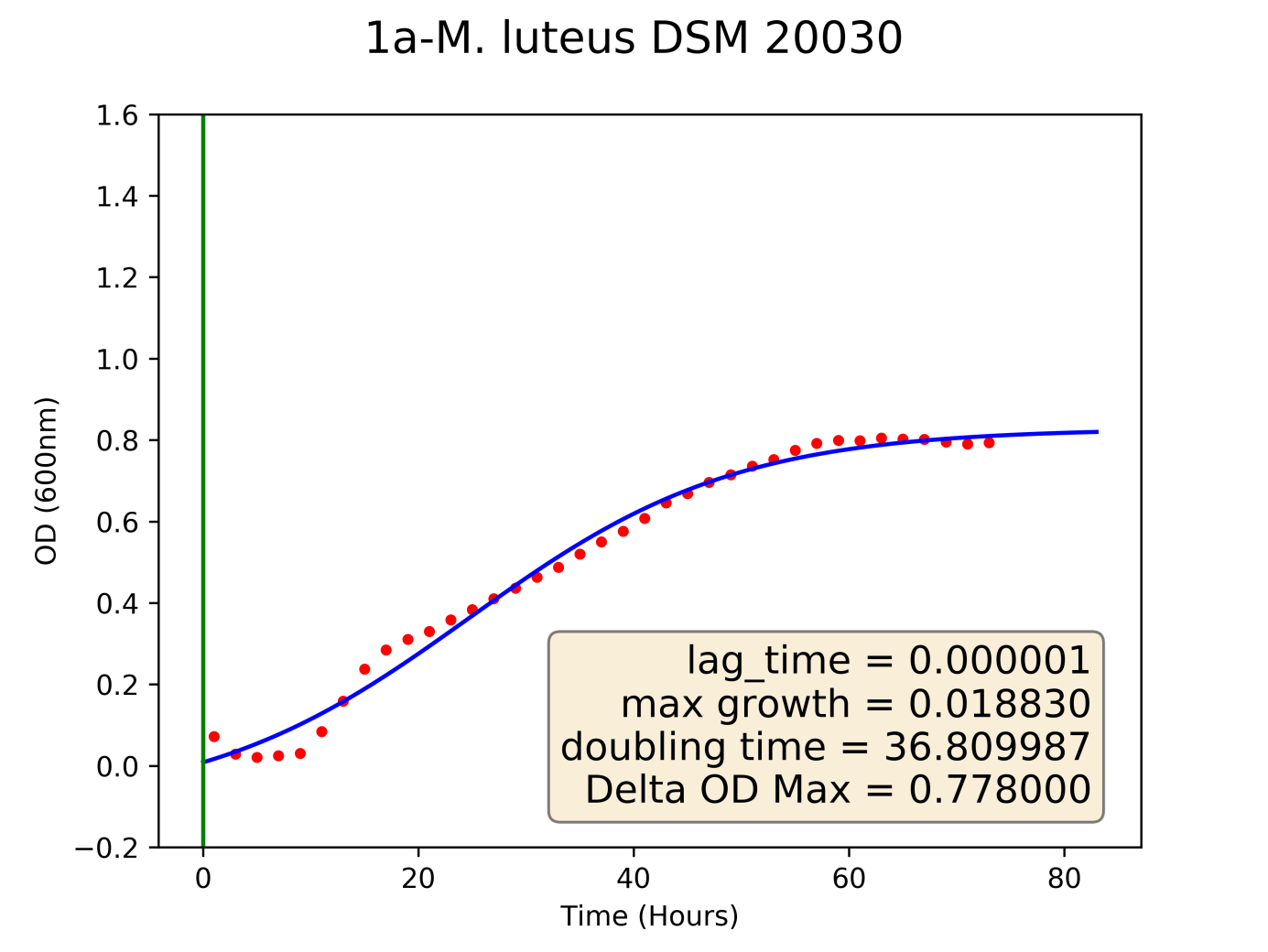


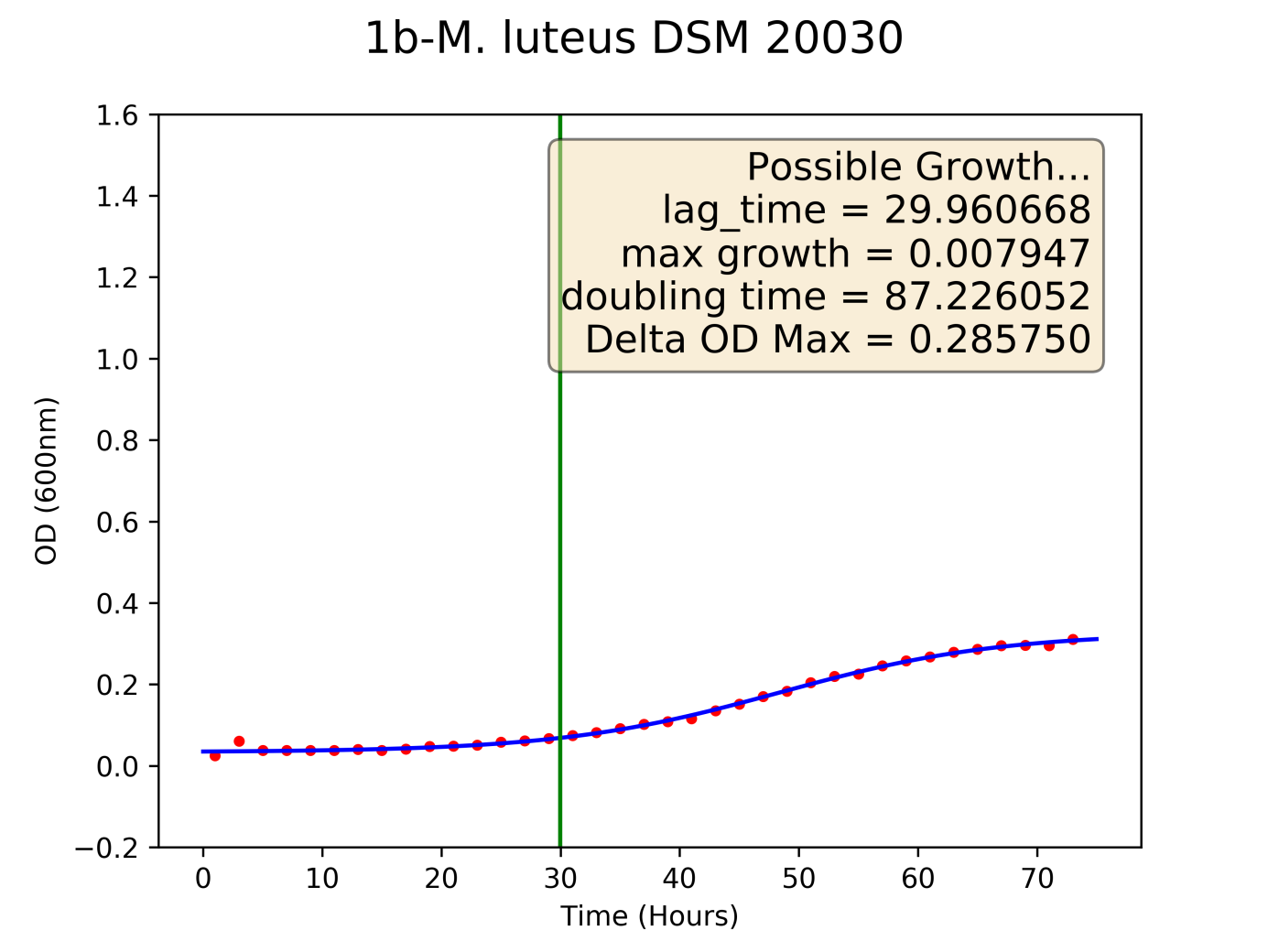


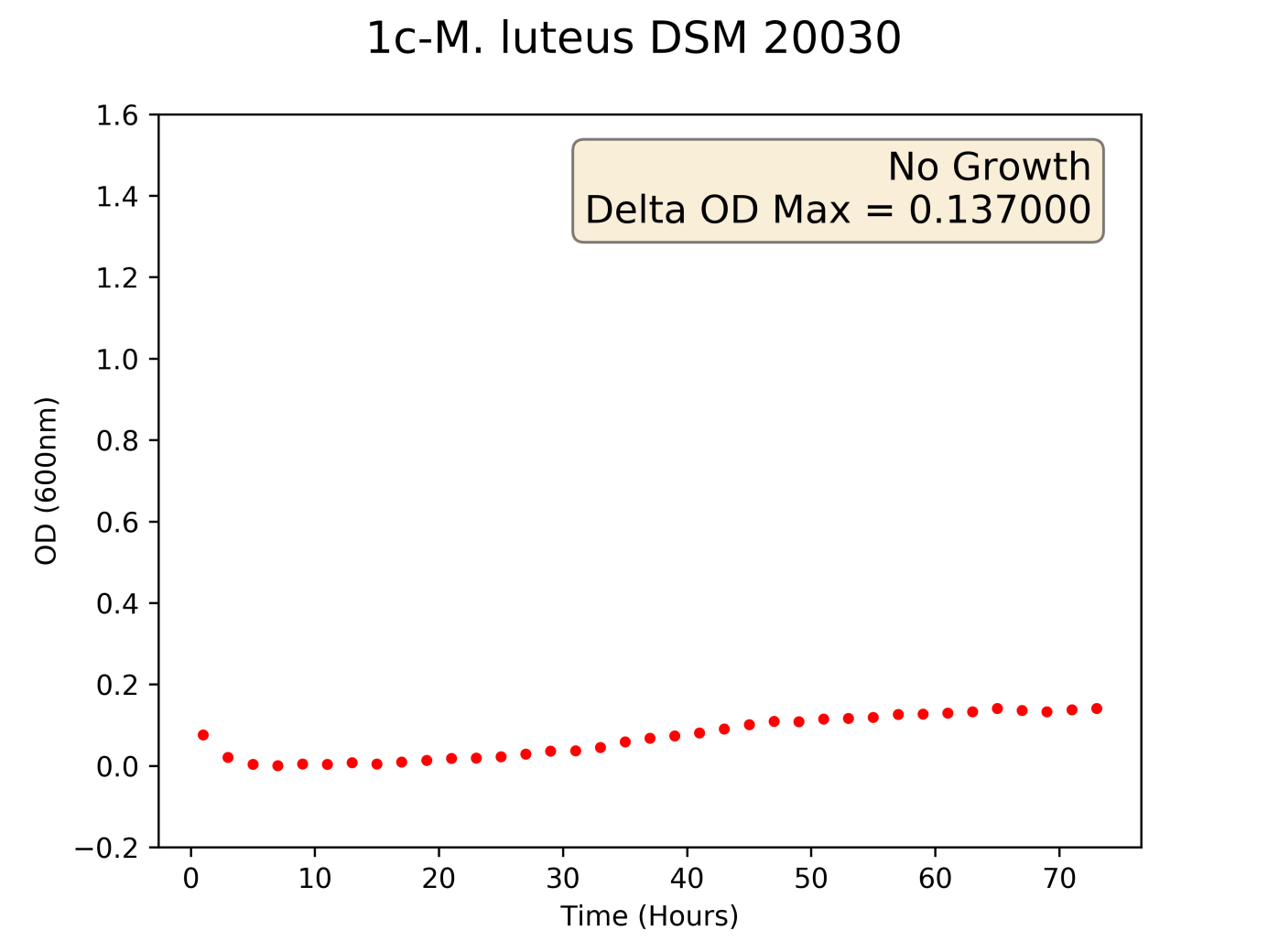


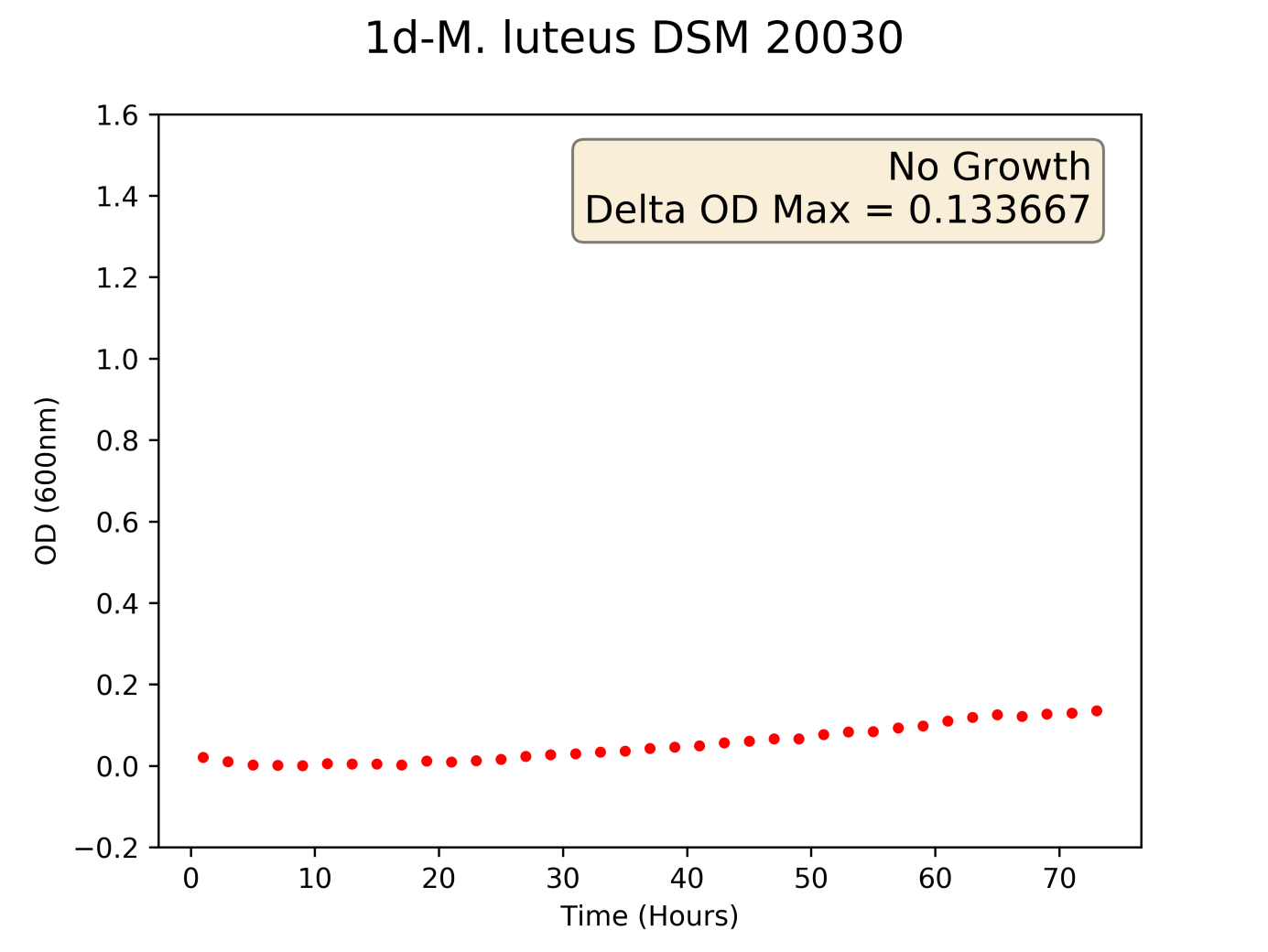


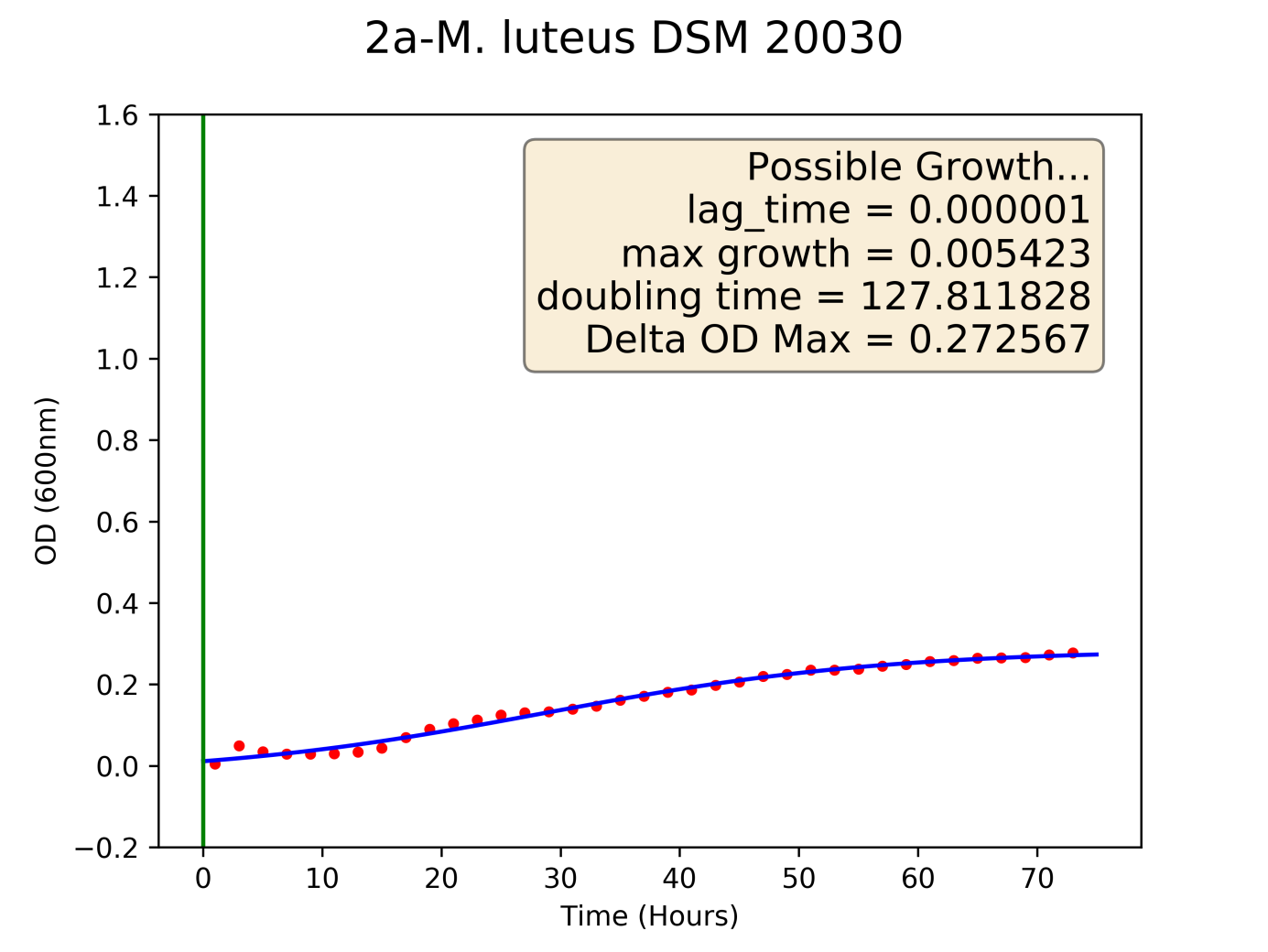


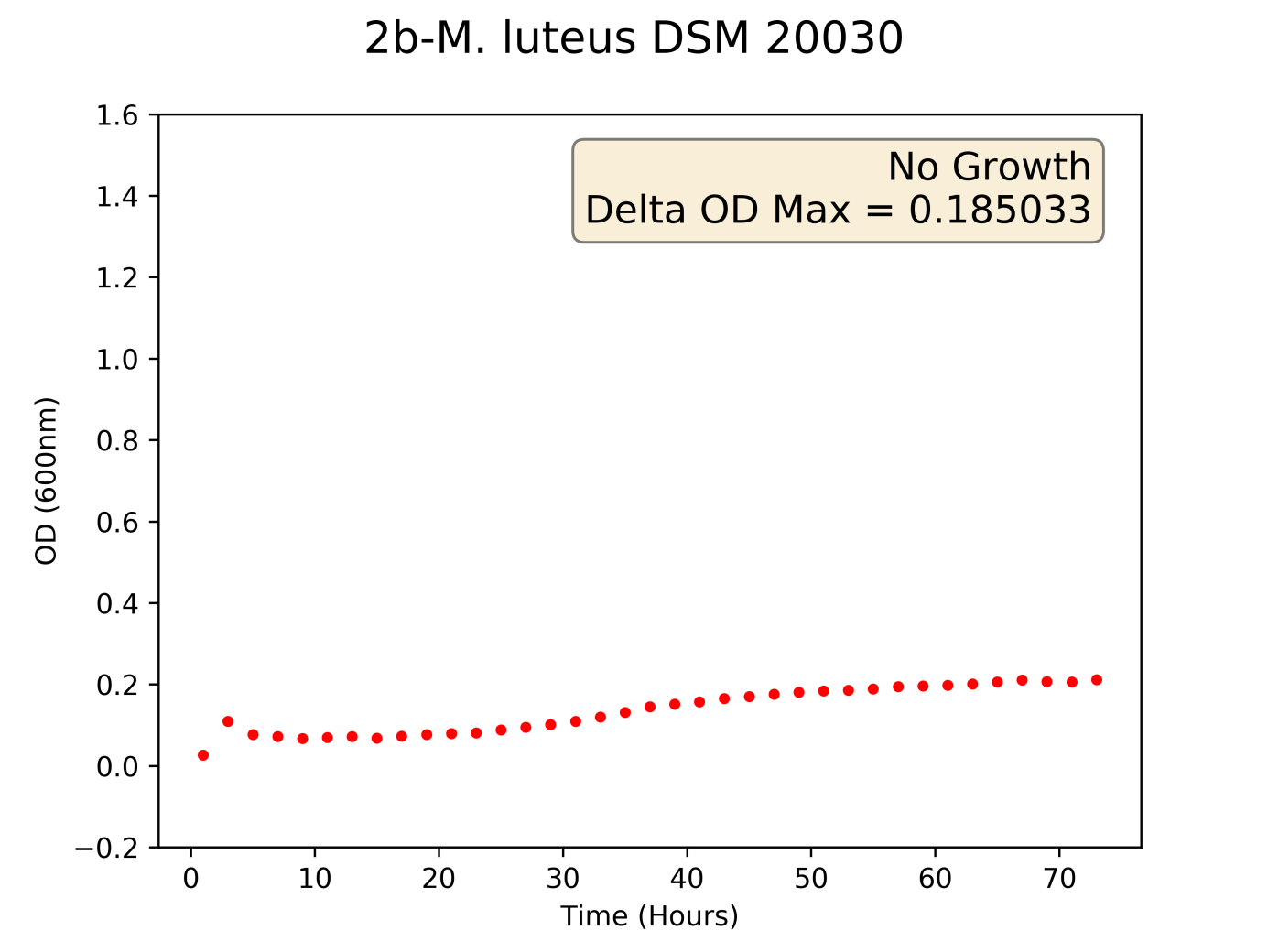


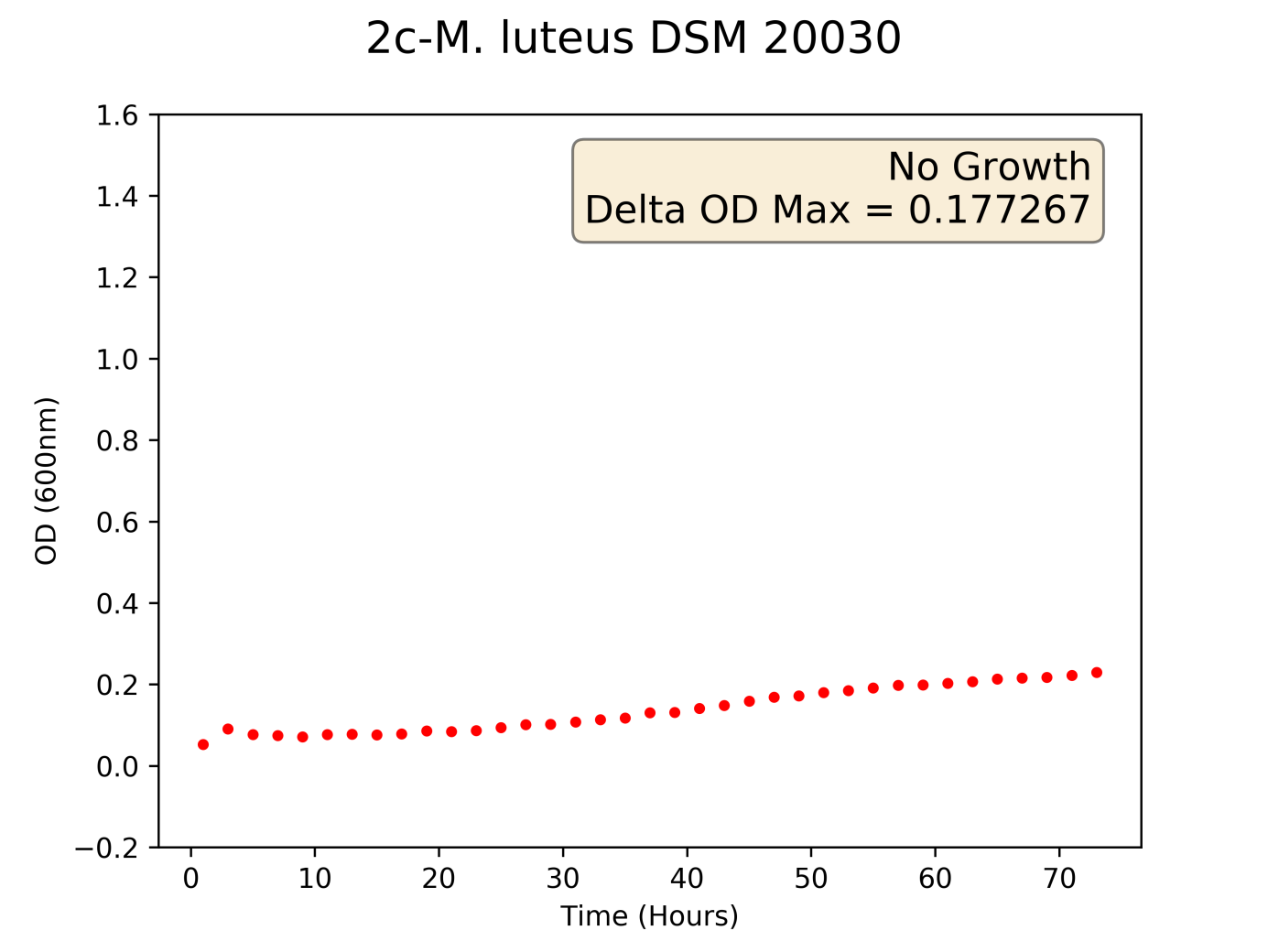


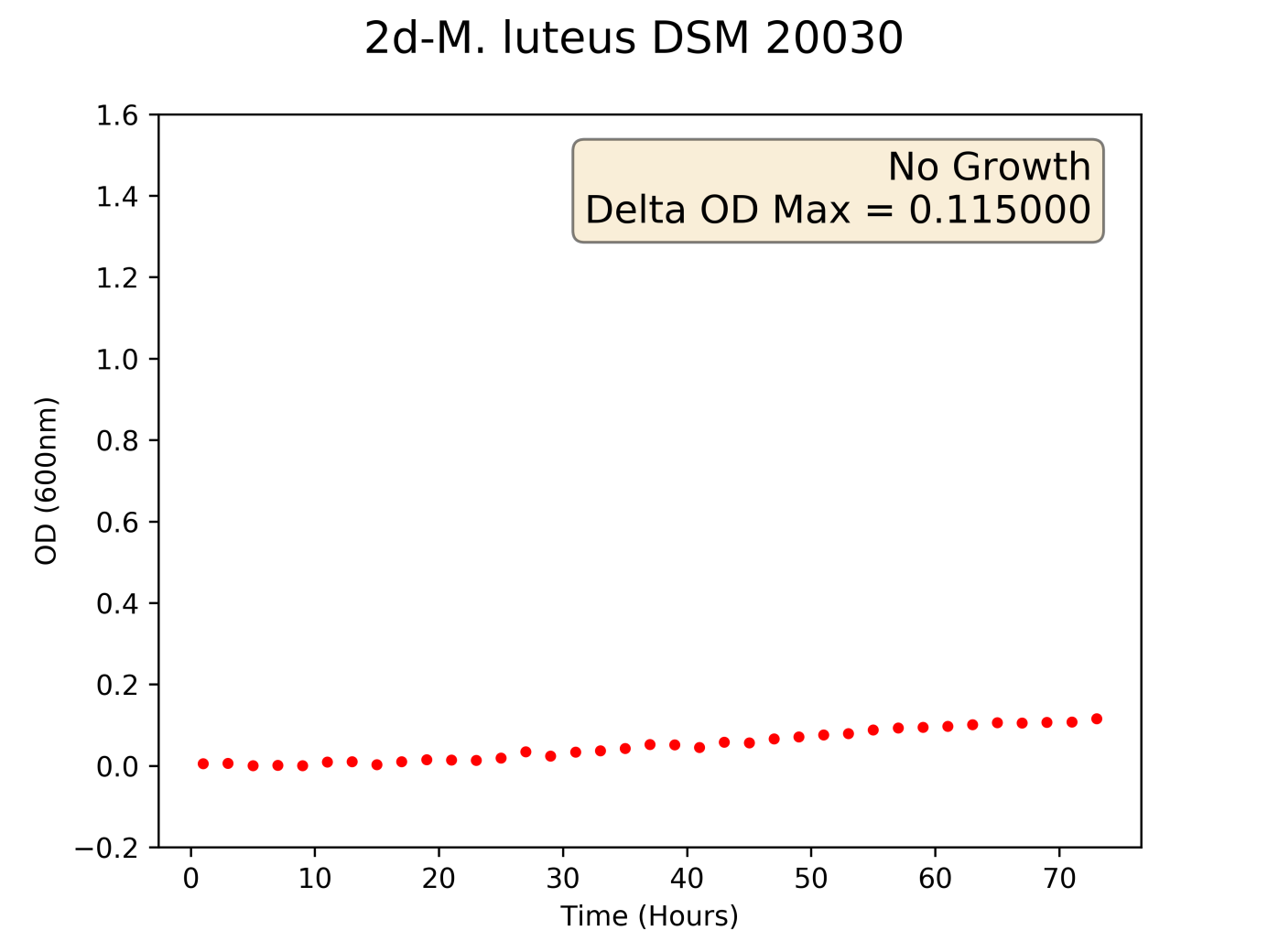


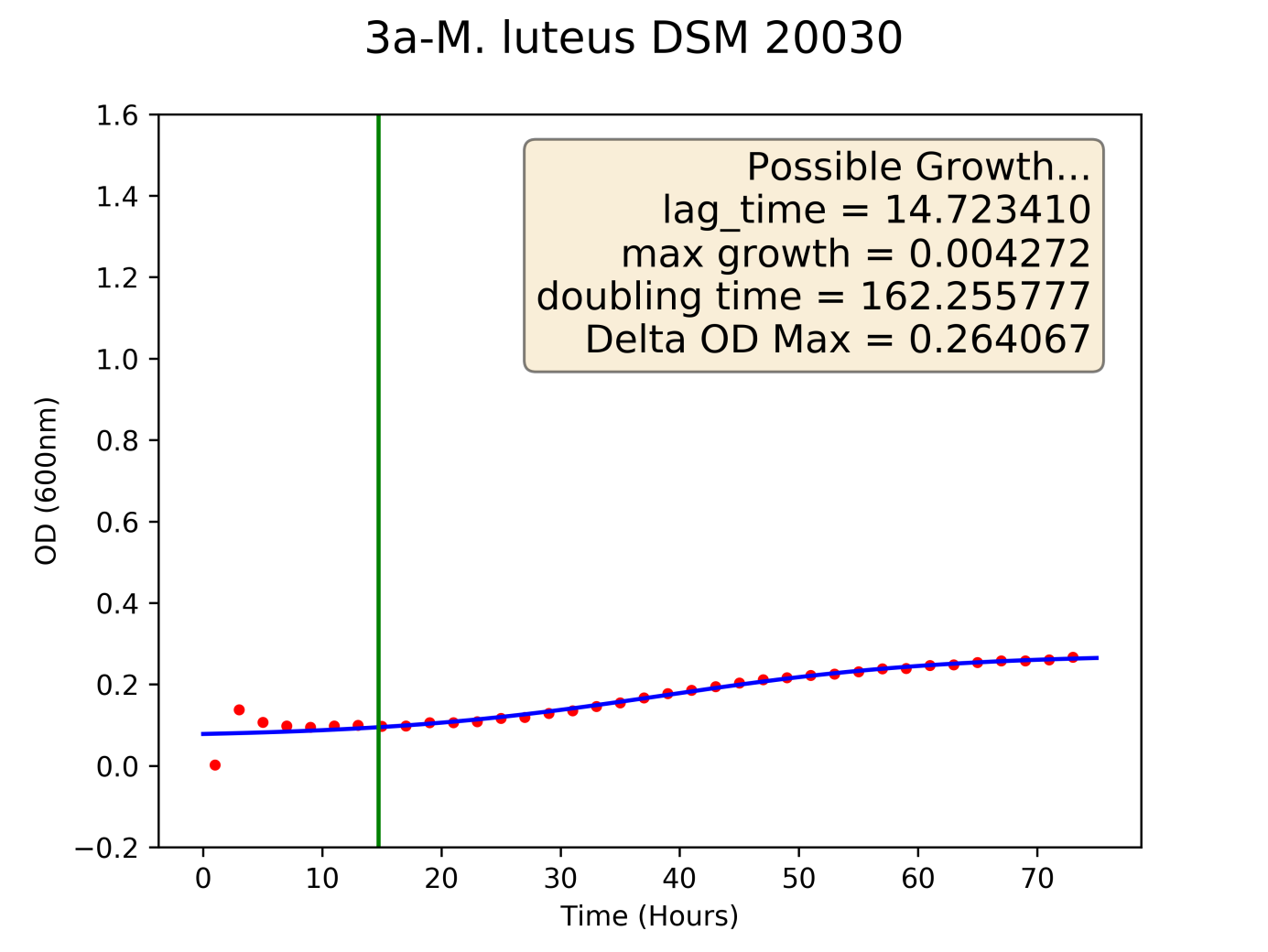


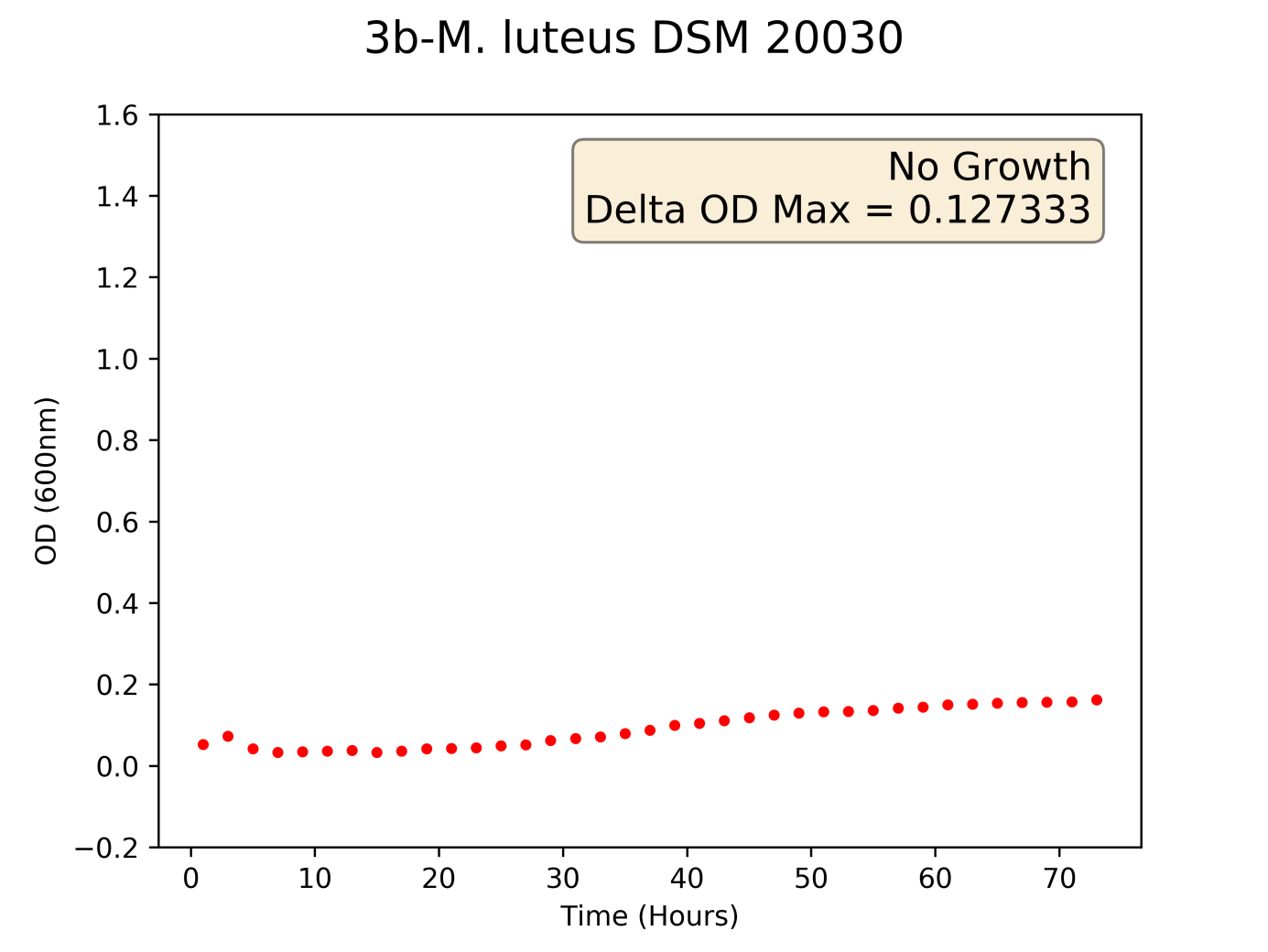


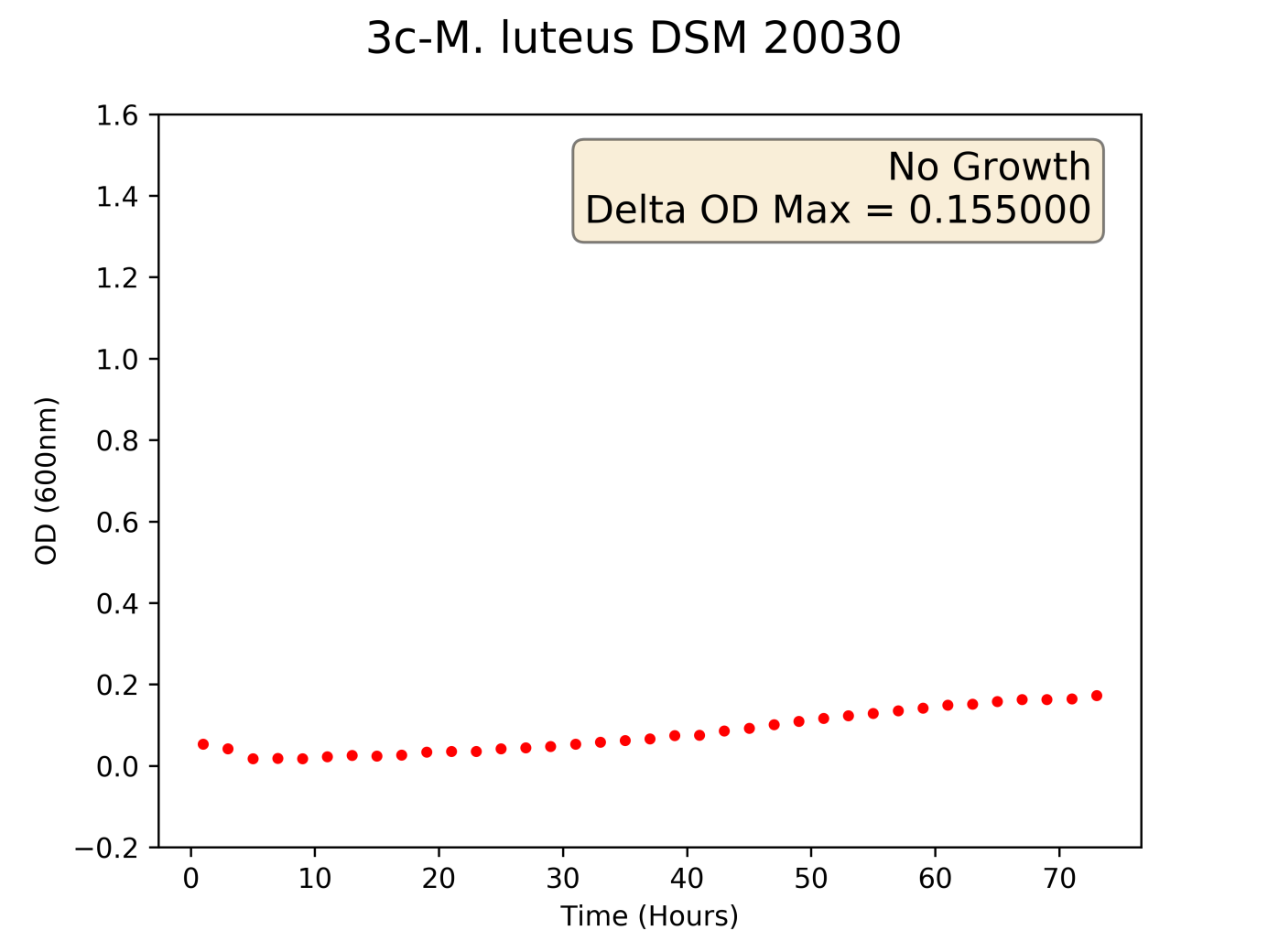


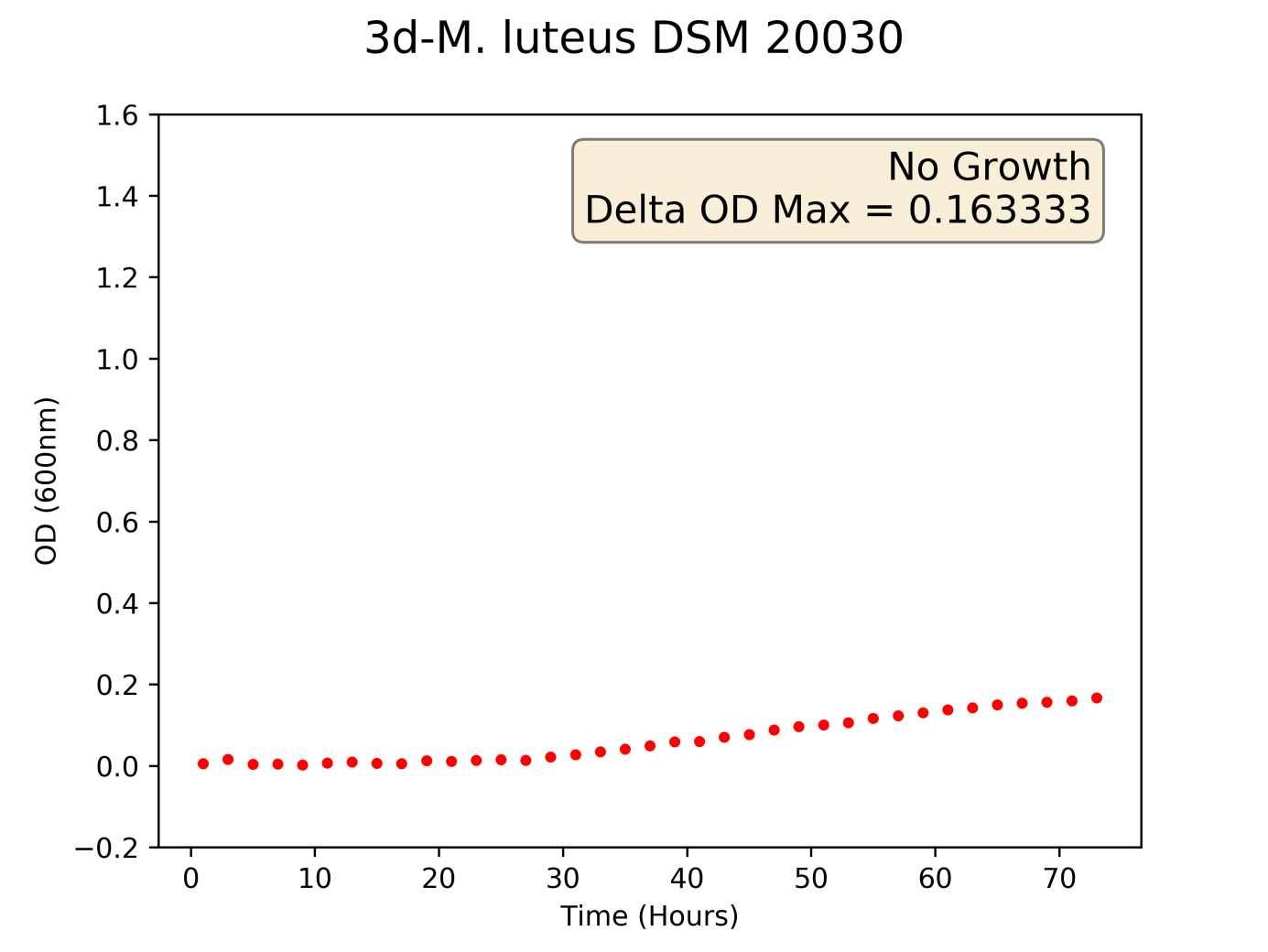


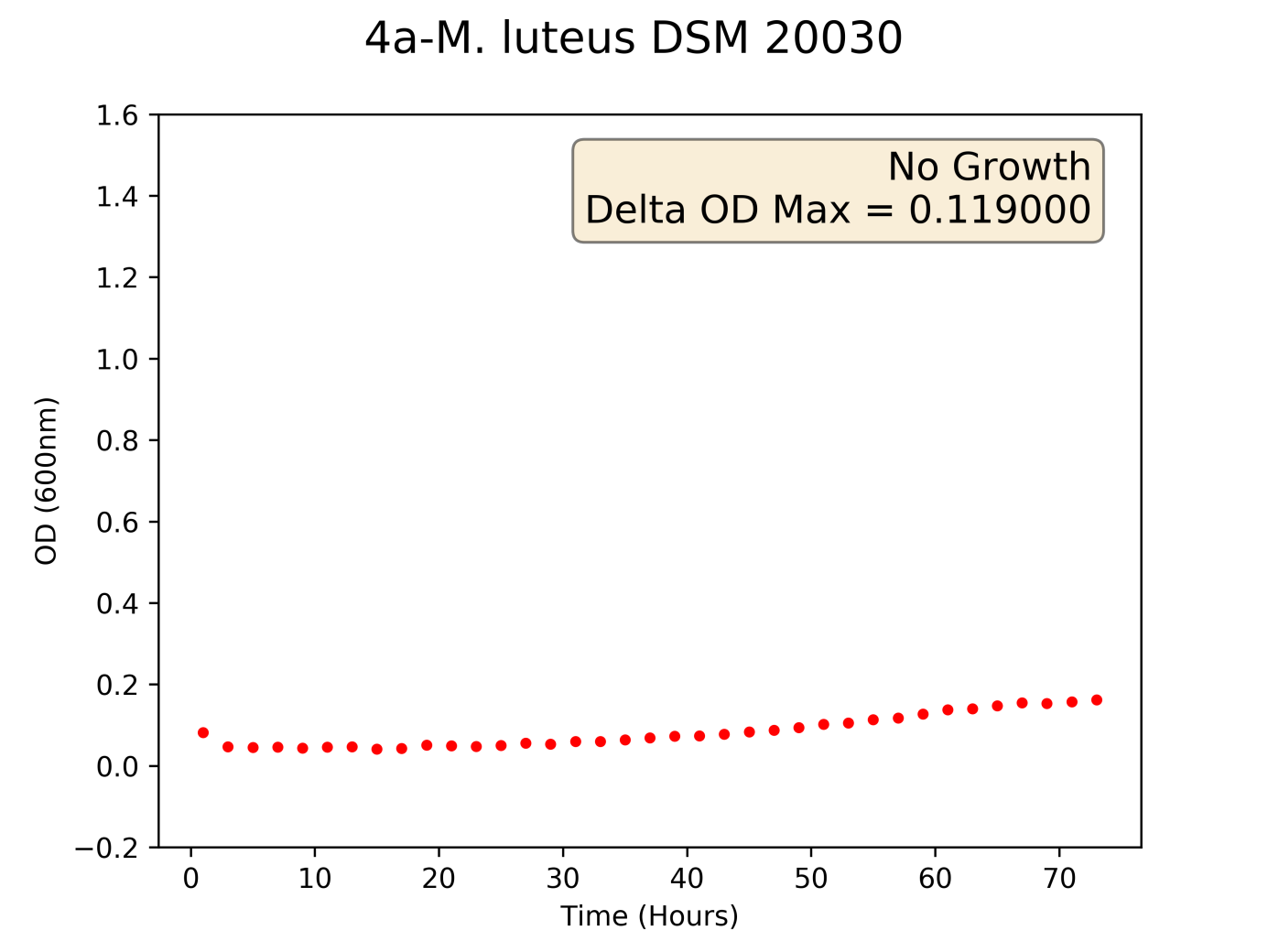


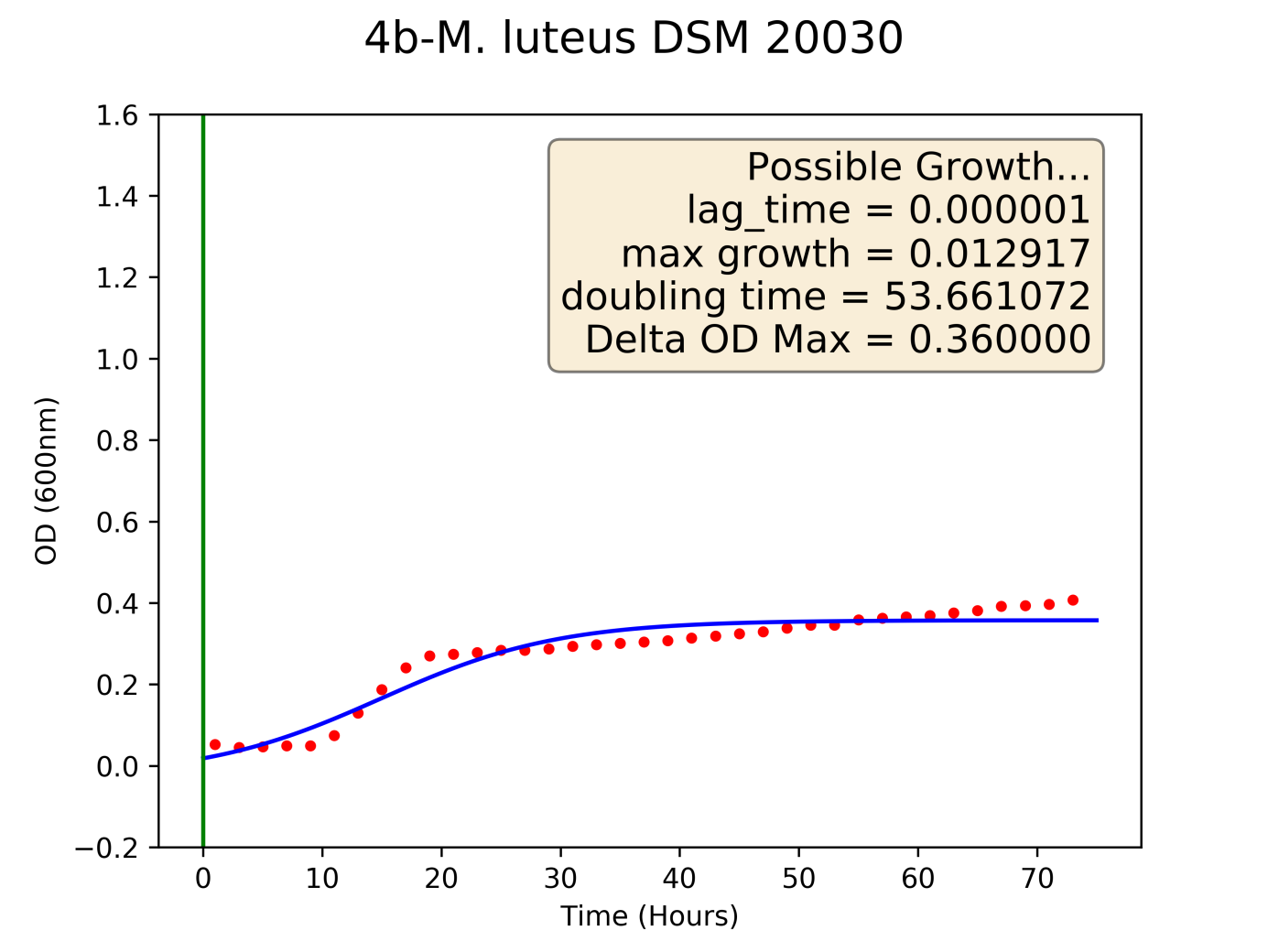


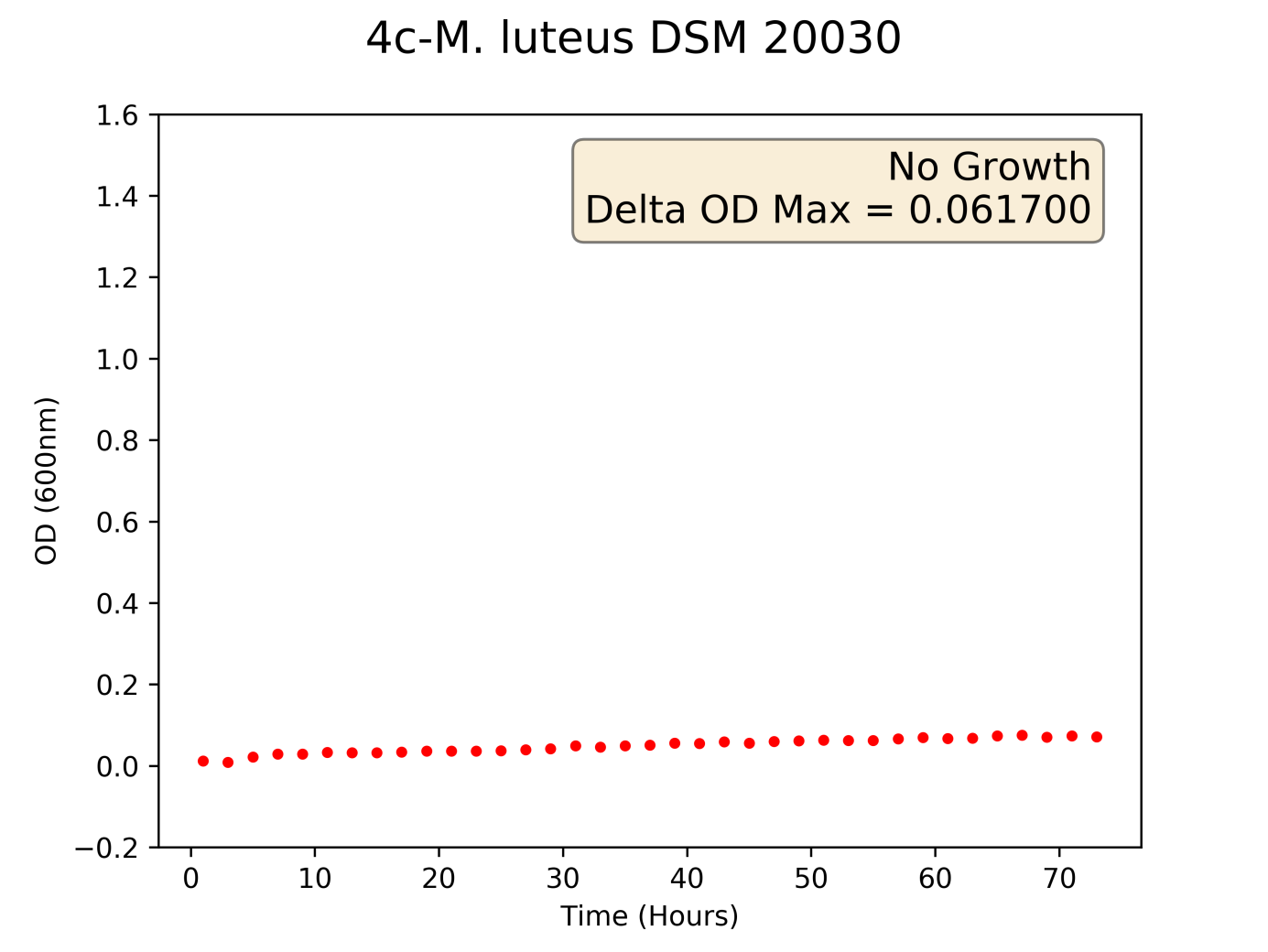


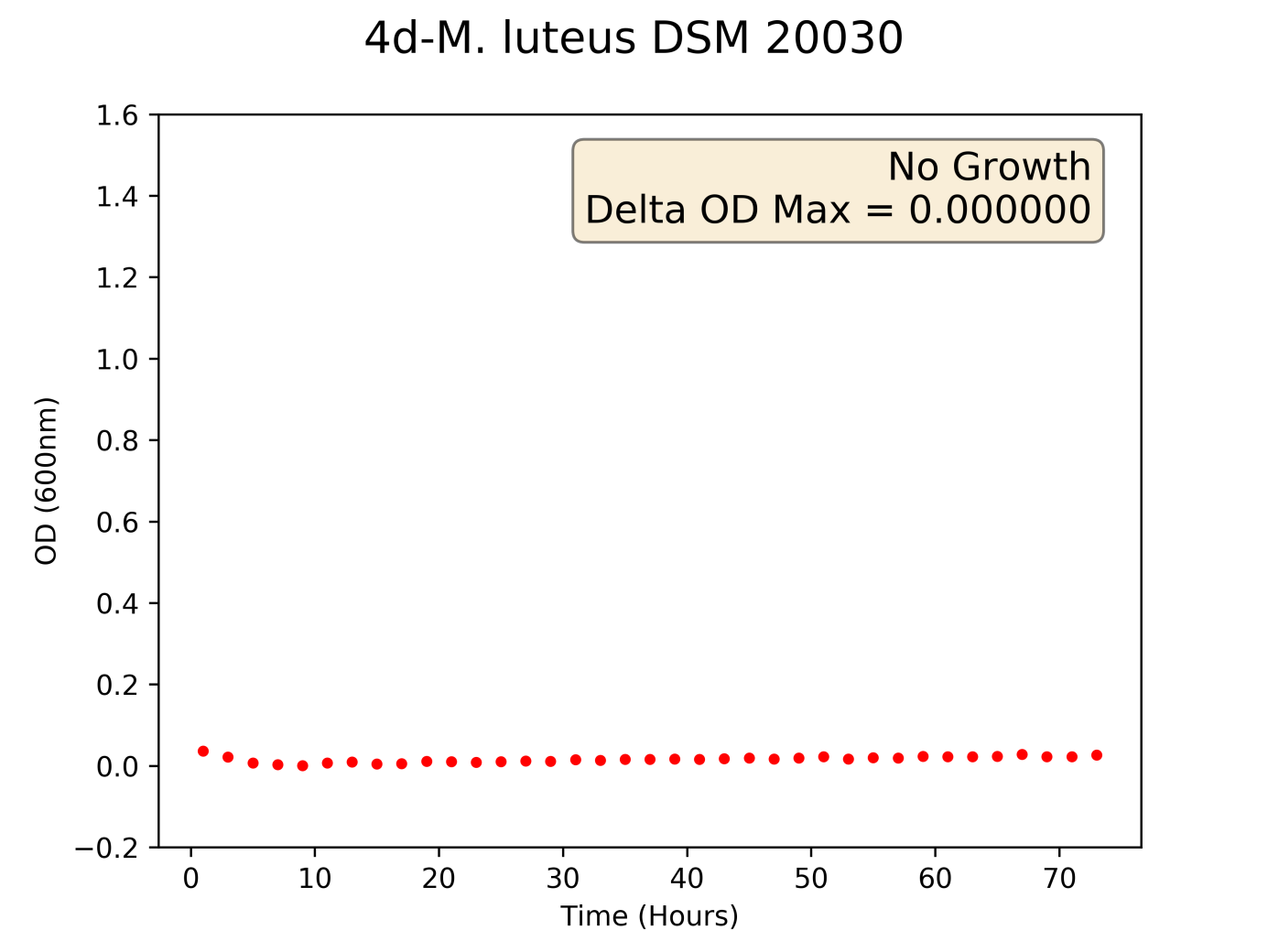


**Fig. S1.** Regression curves of selected data. *M. luteus* (1, 2, 3, 4 – types of TiO2; a, b, c, d – concentration of TiO2 : 60, 150, 300 i 600 mg/L)

Supplement: Supplementary file 1 — (ZIP 8.20 MB) [file 12011_2021_2843_MOESM1_ESM.zip › 2S3 (M. luteus)_ESM.docx]

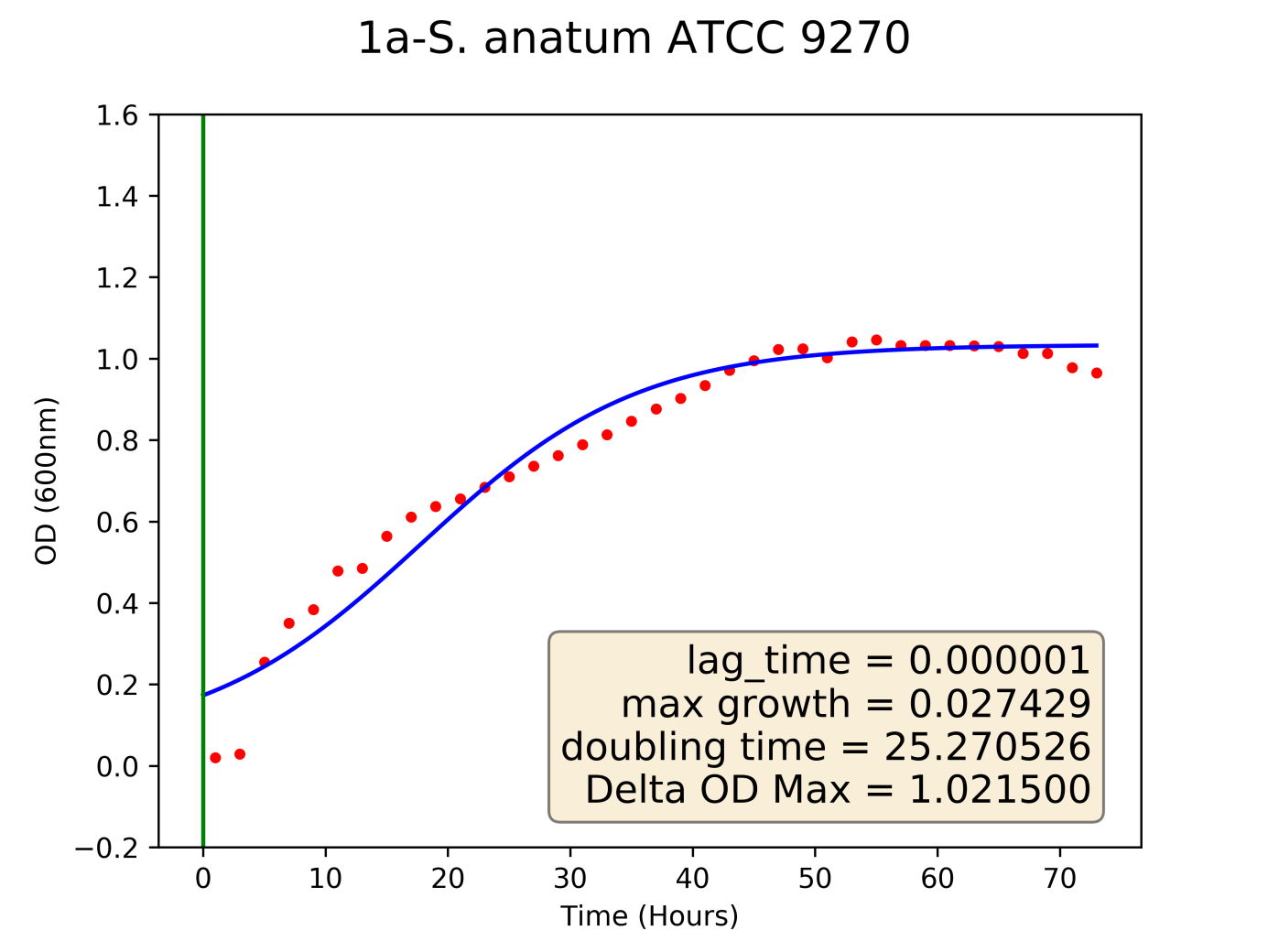


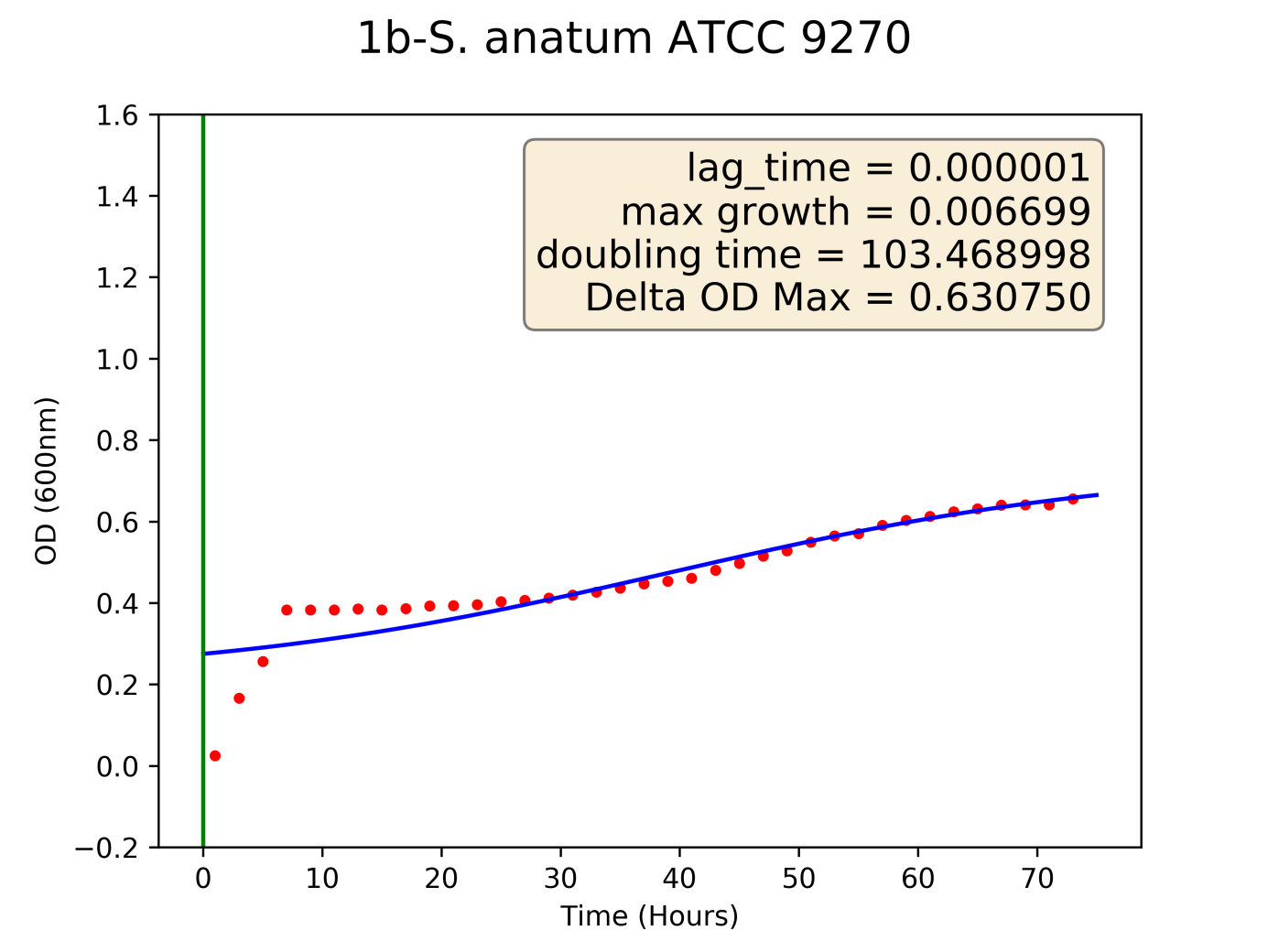


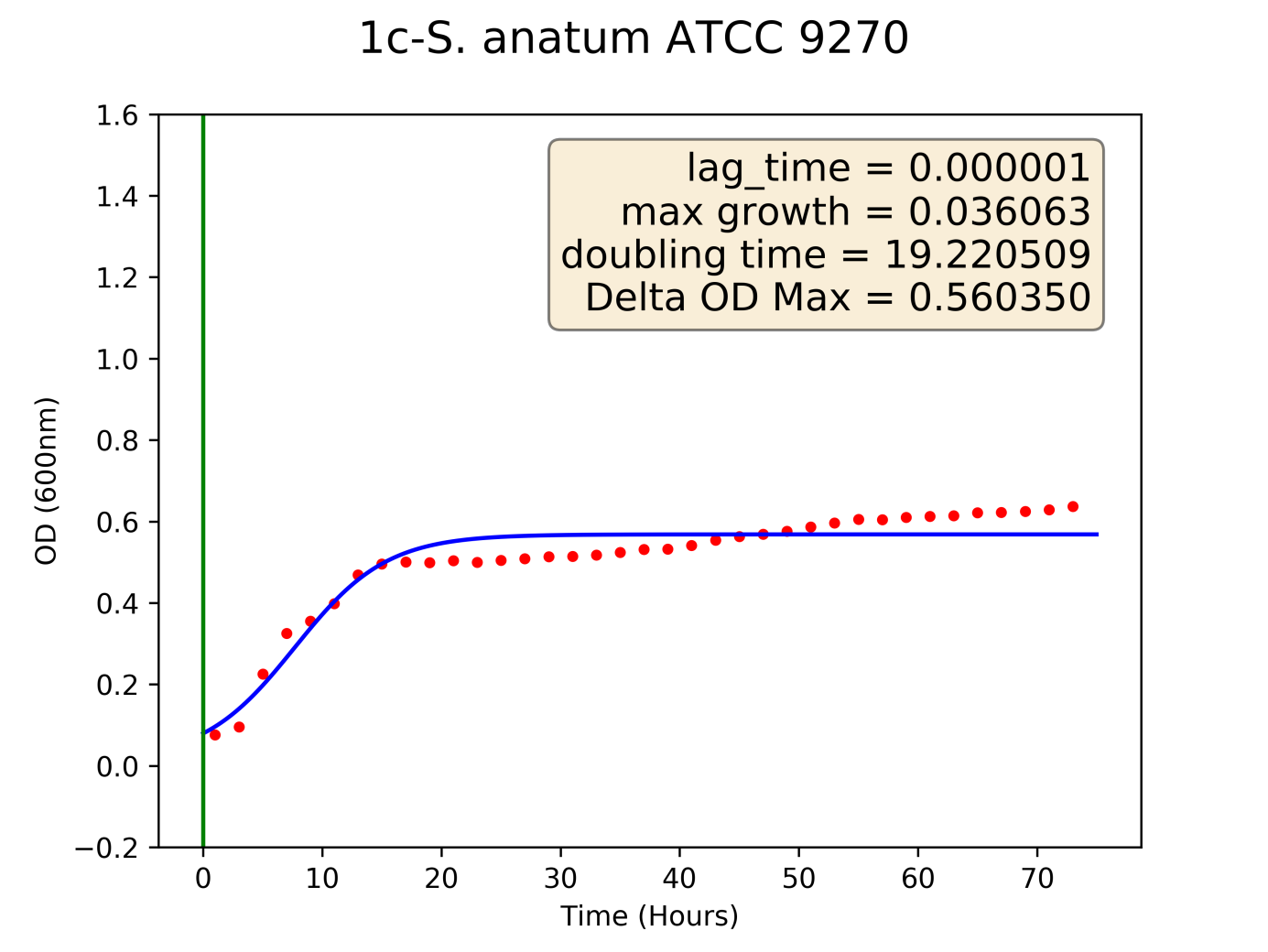


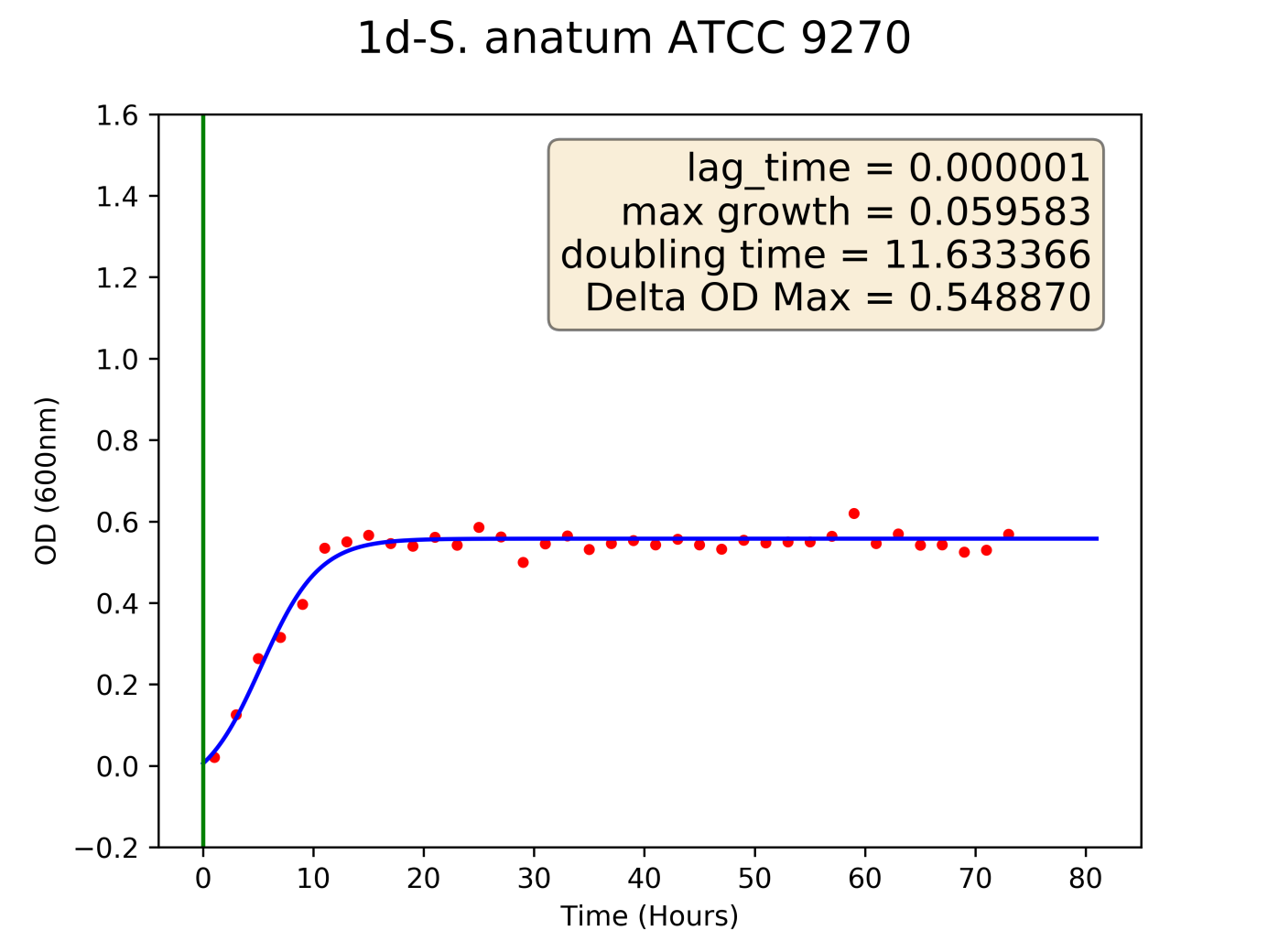


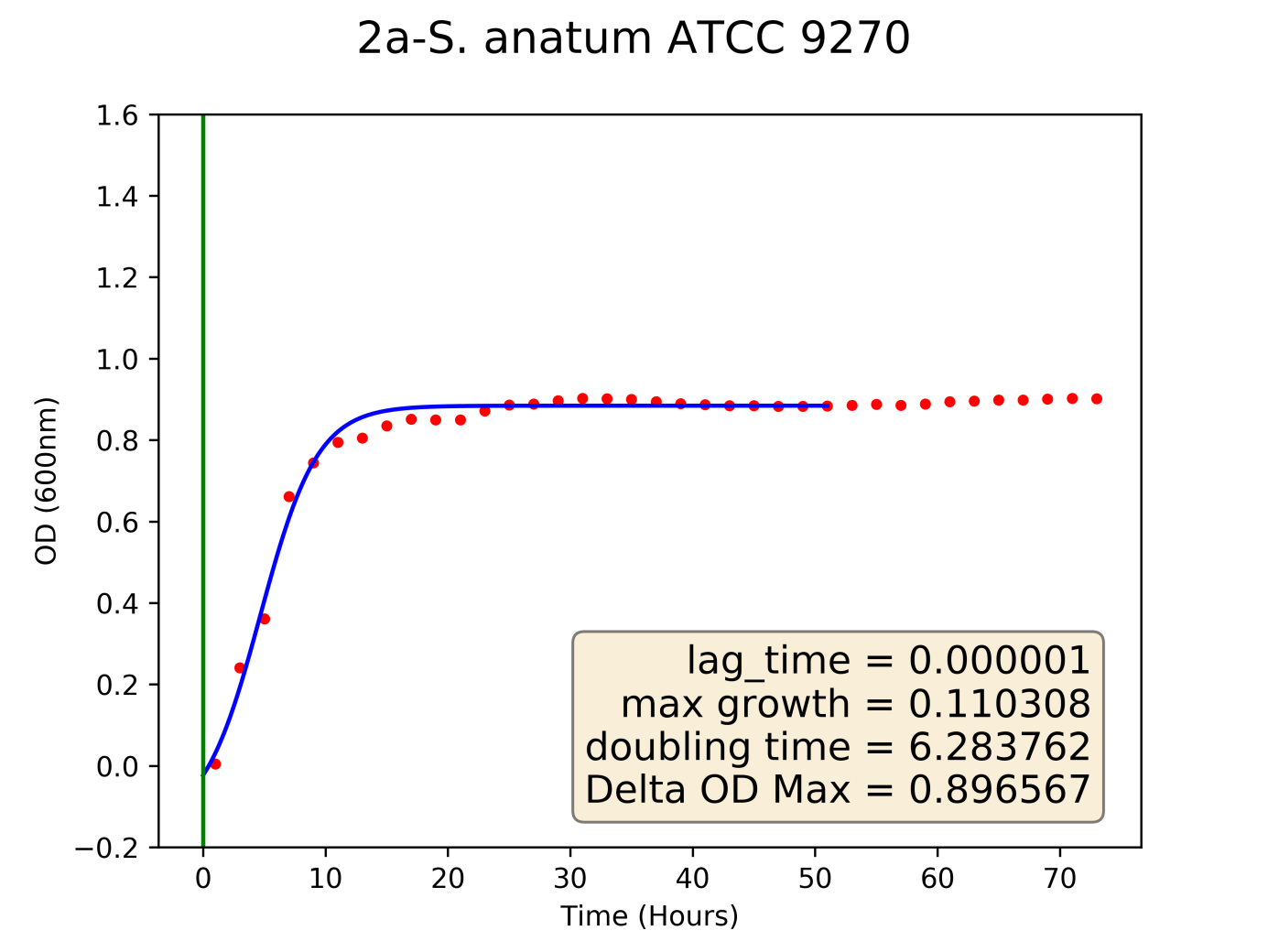


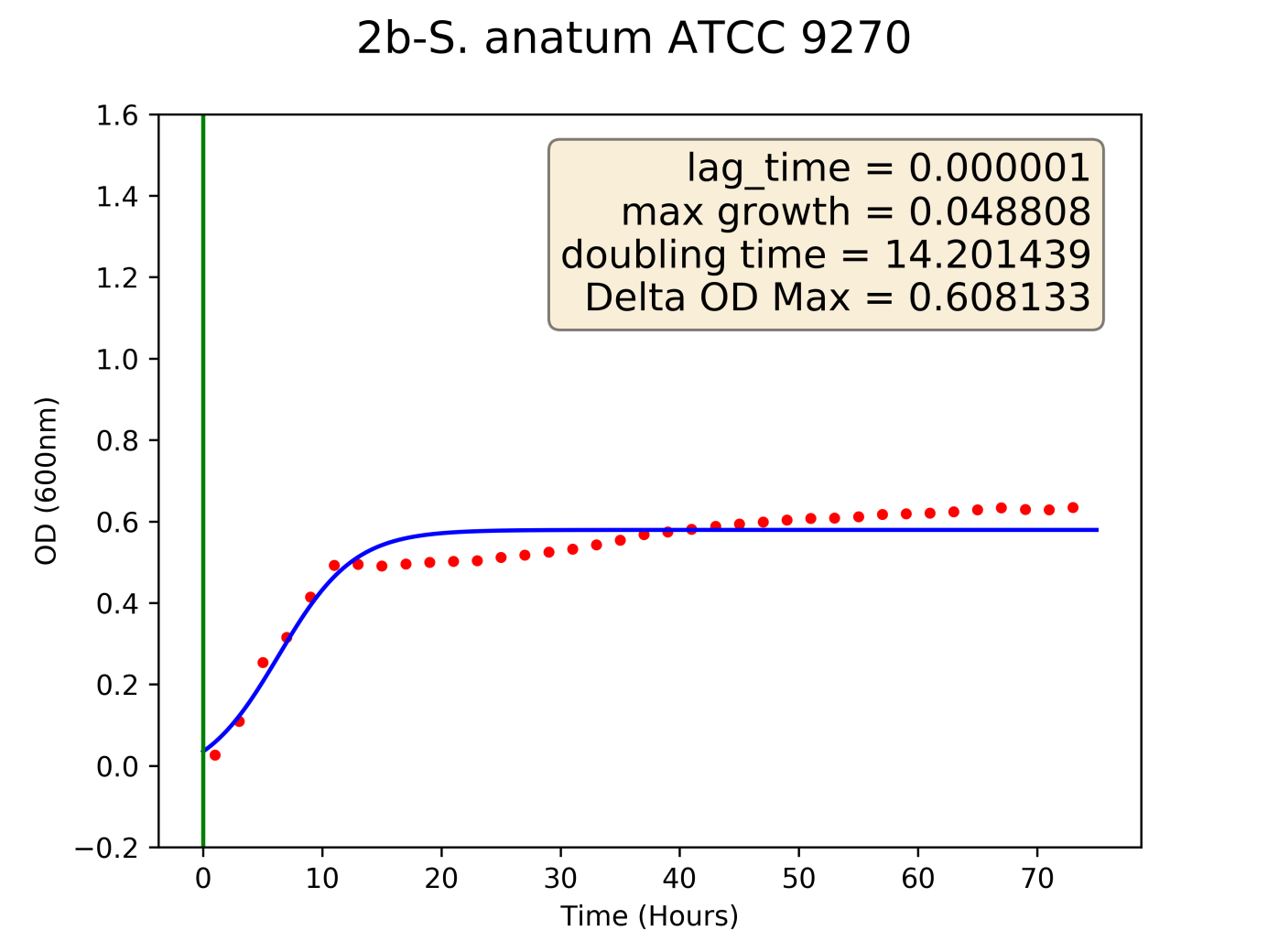


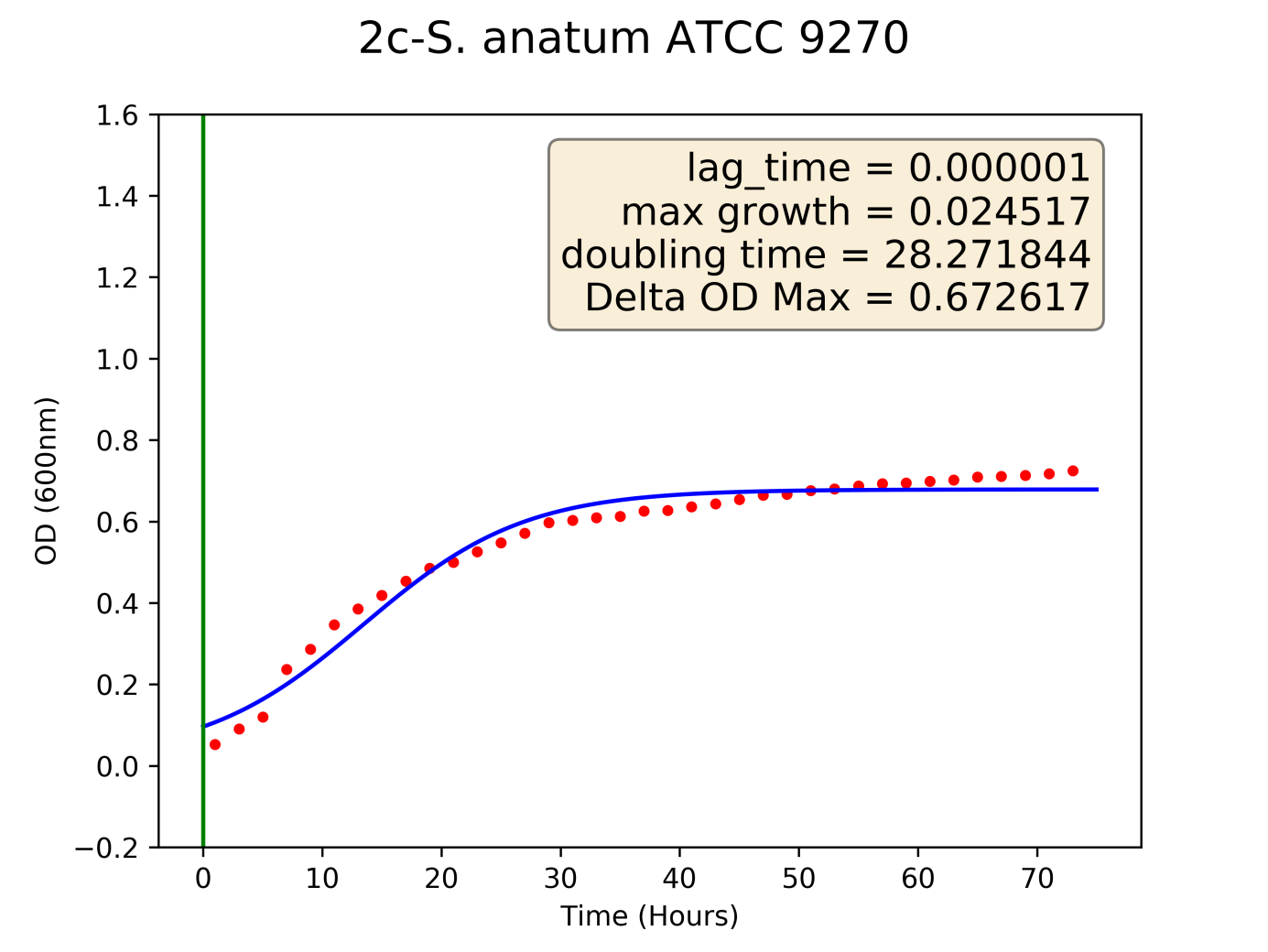


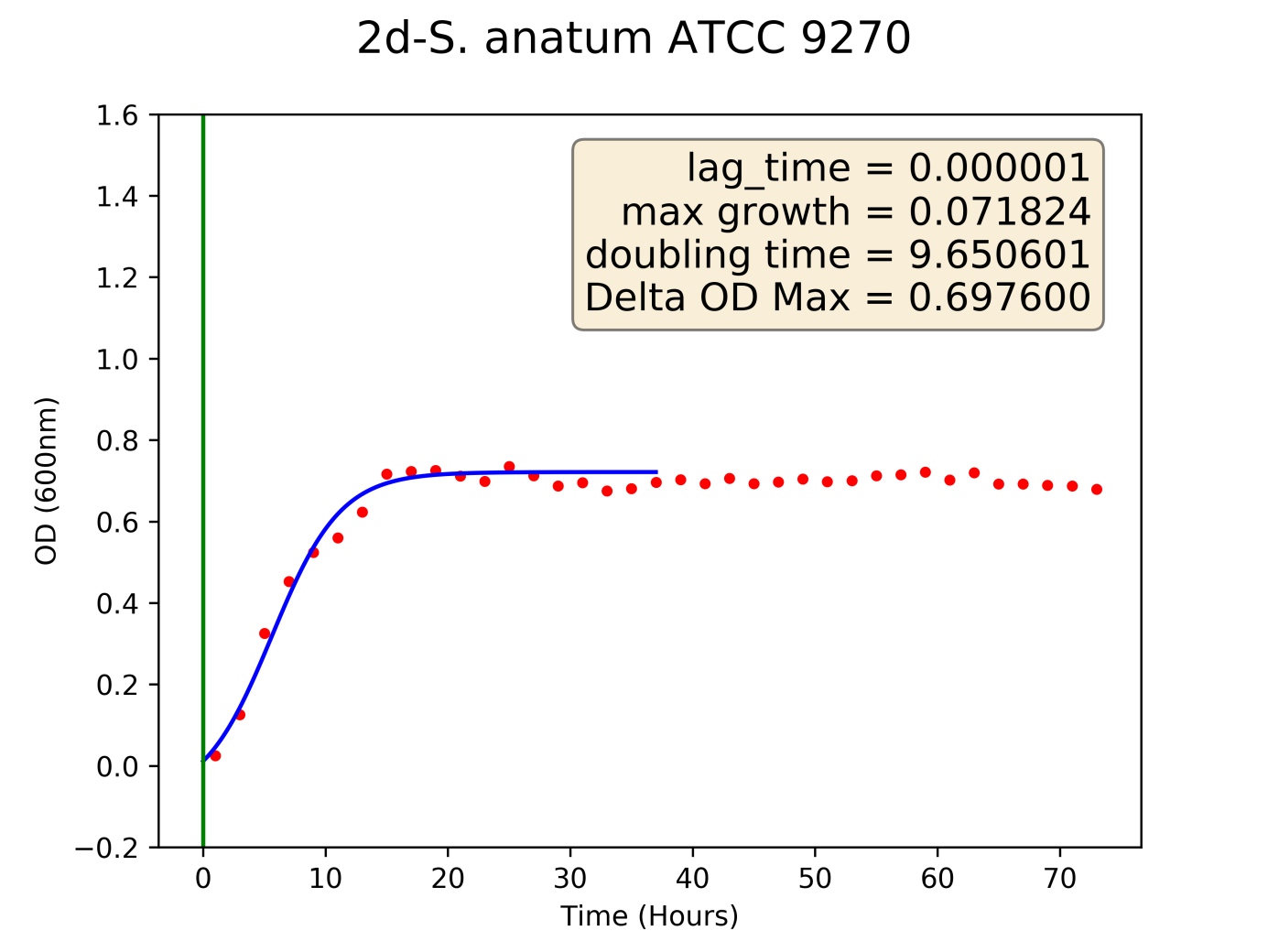

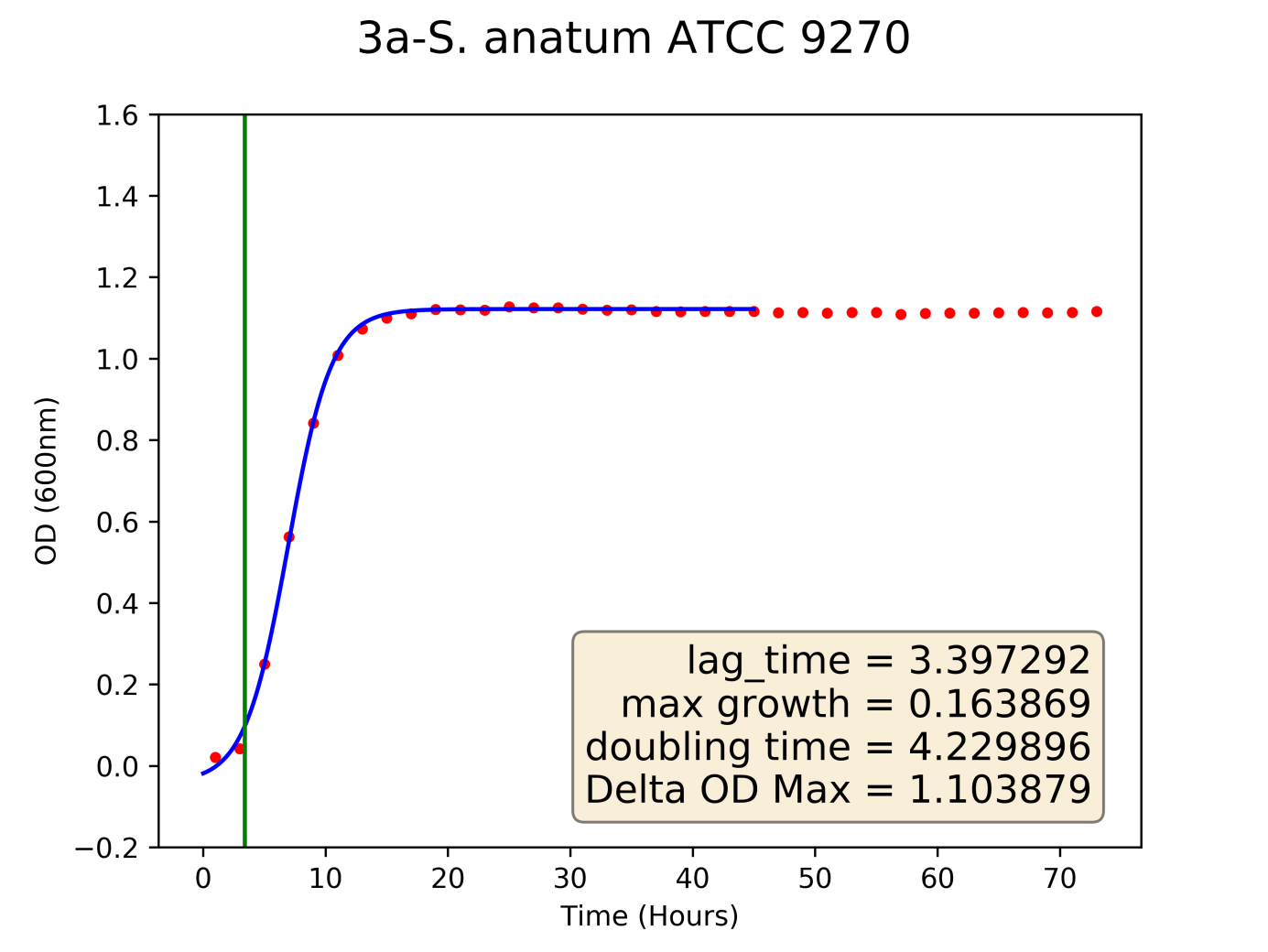


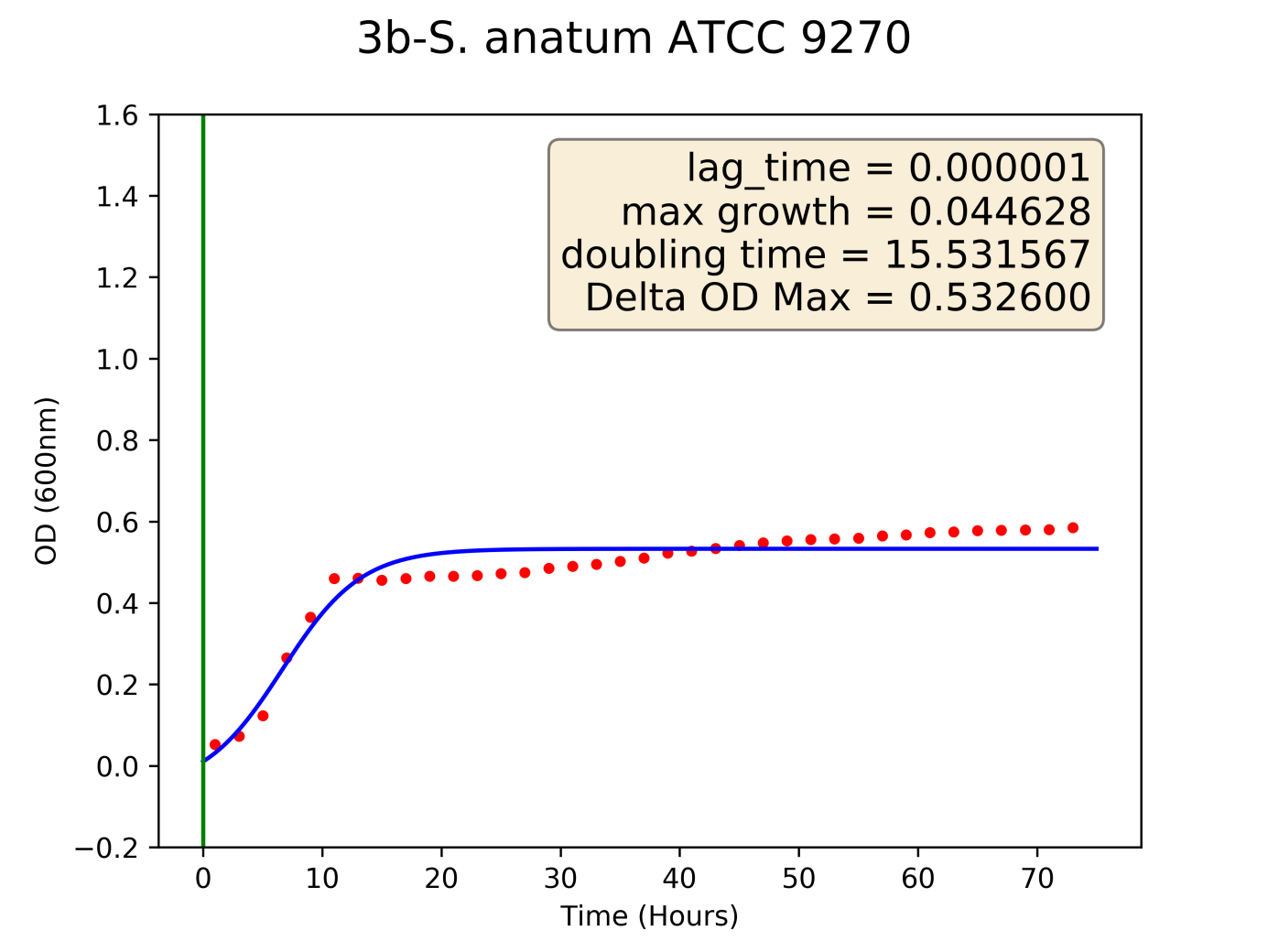


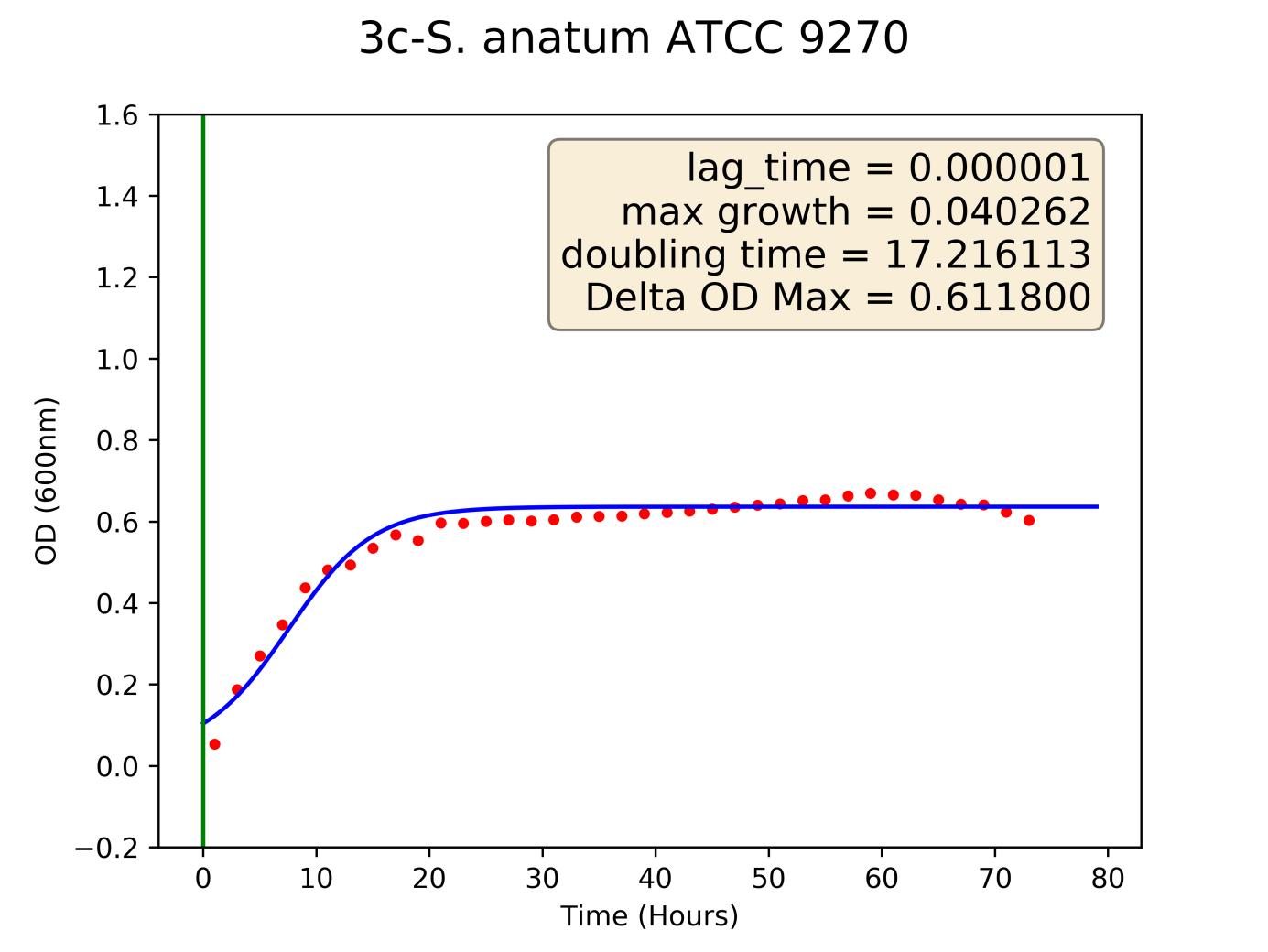


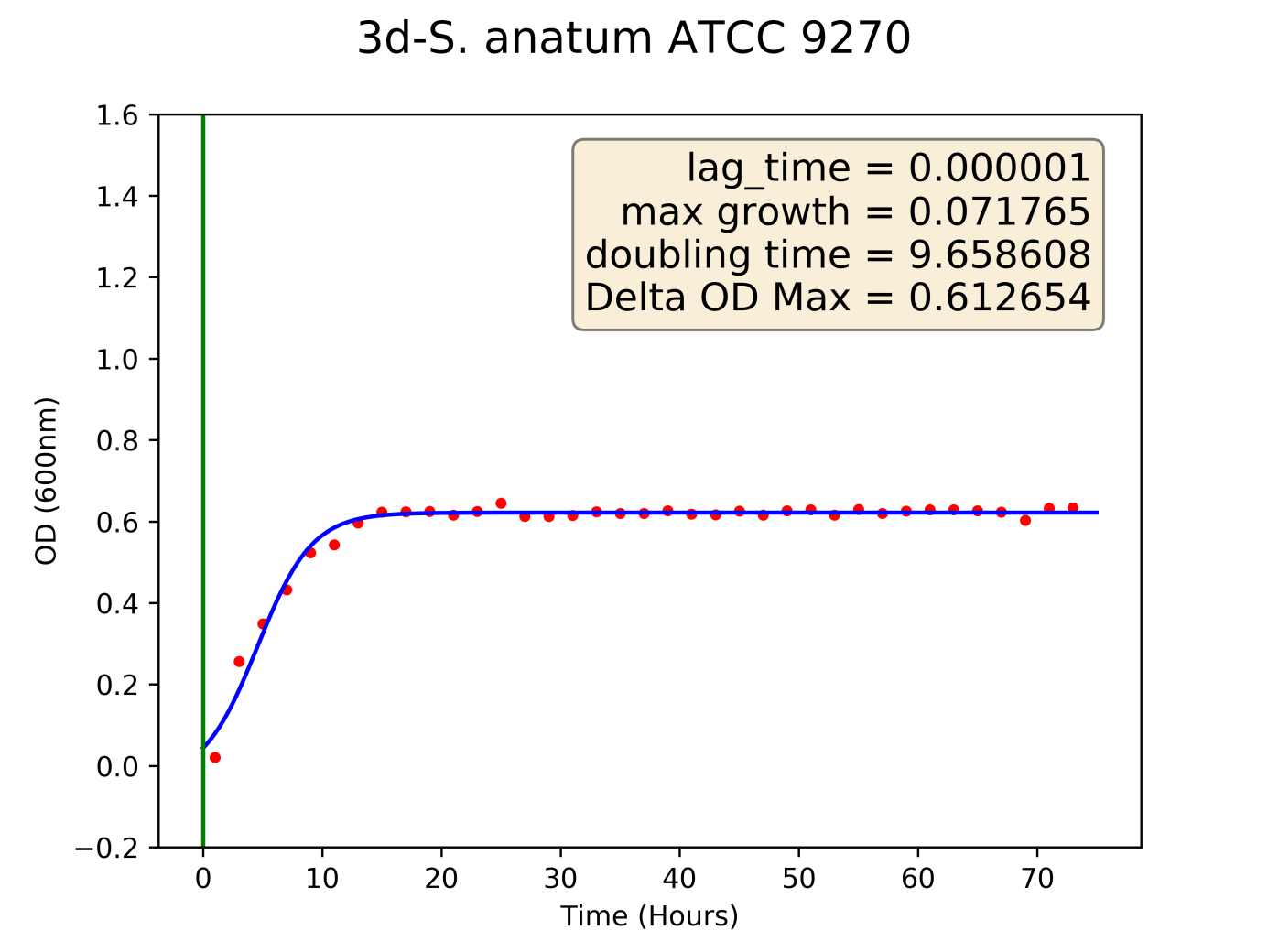


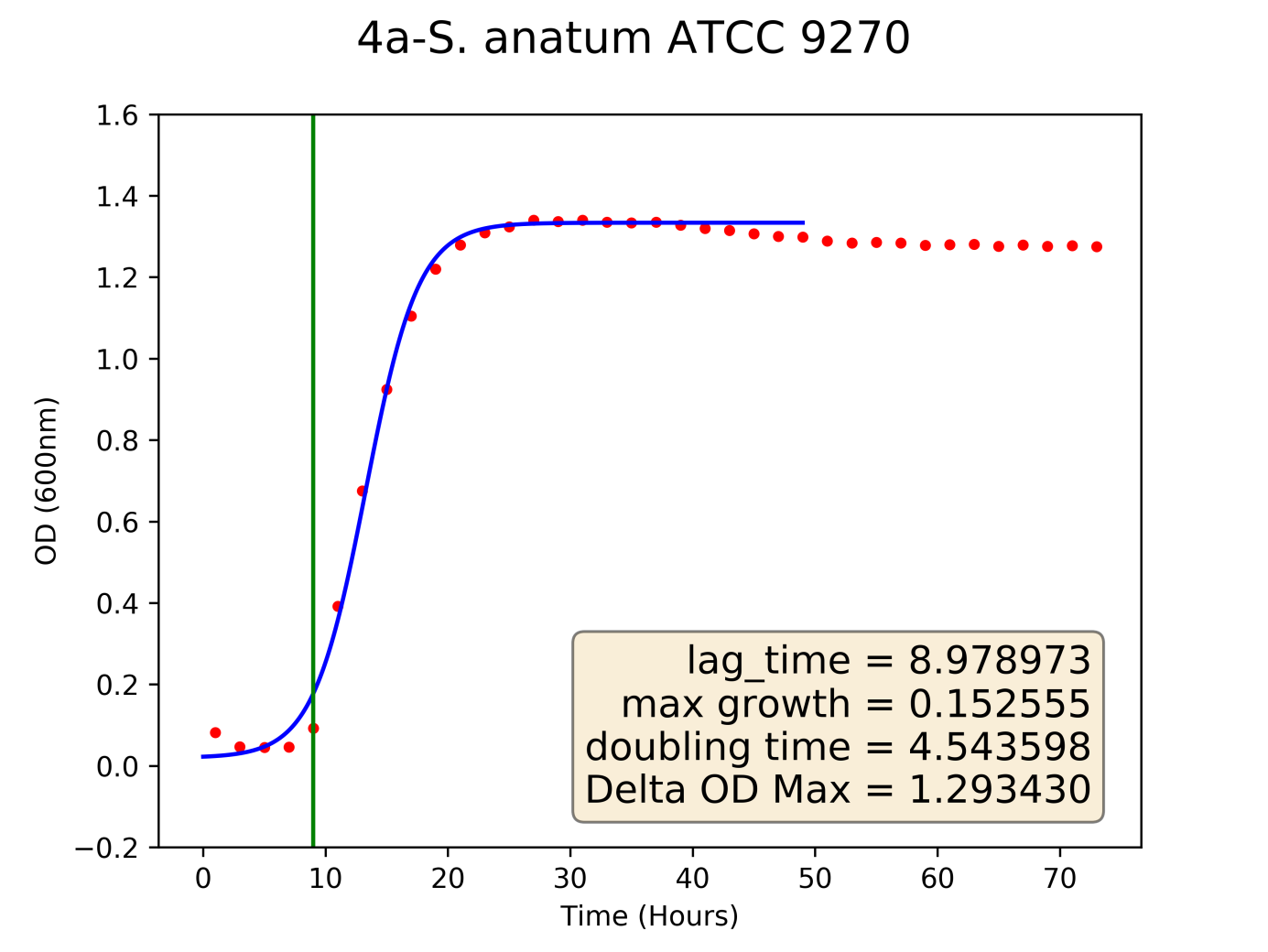


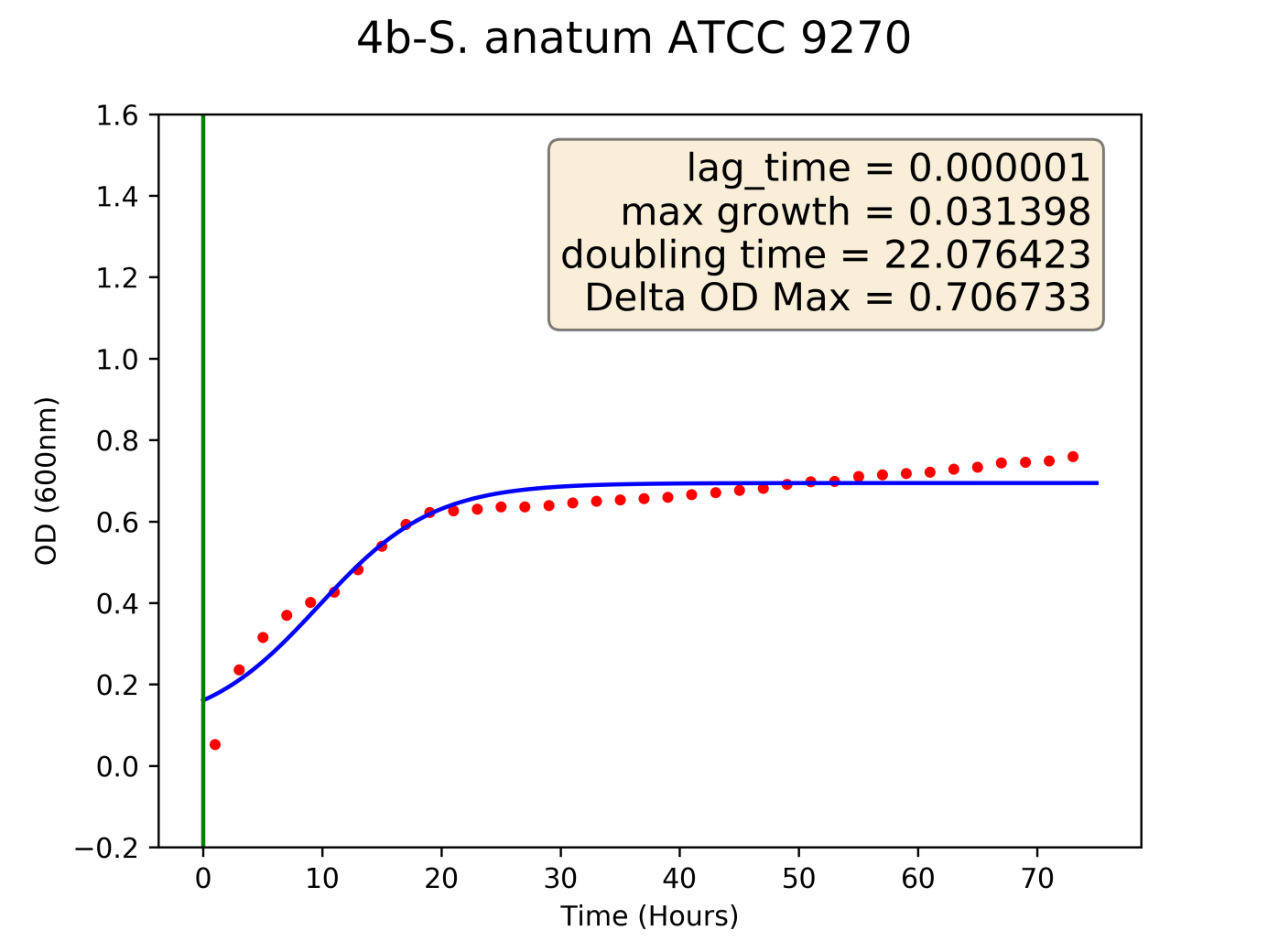


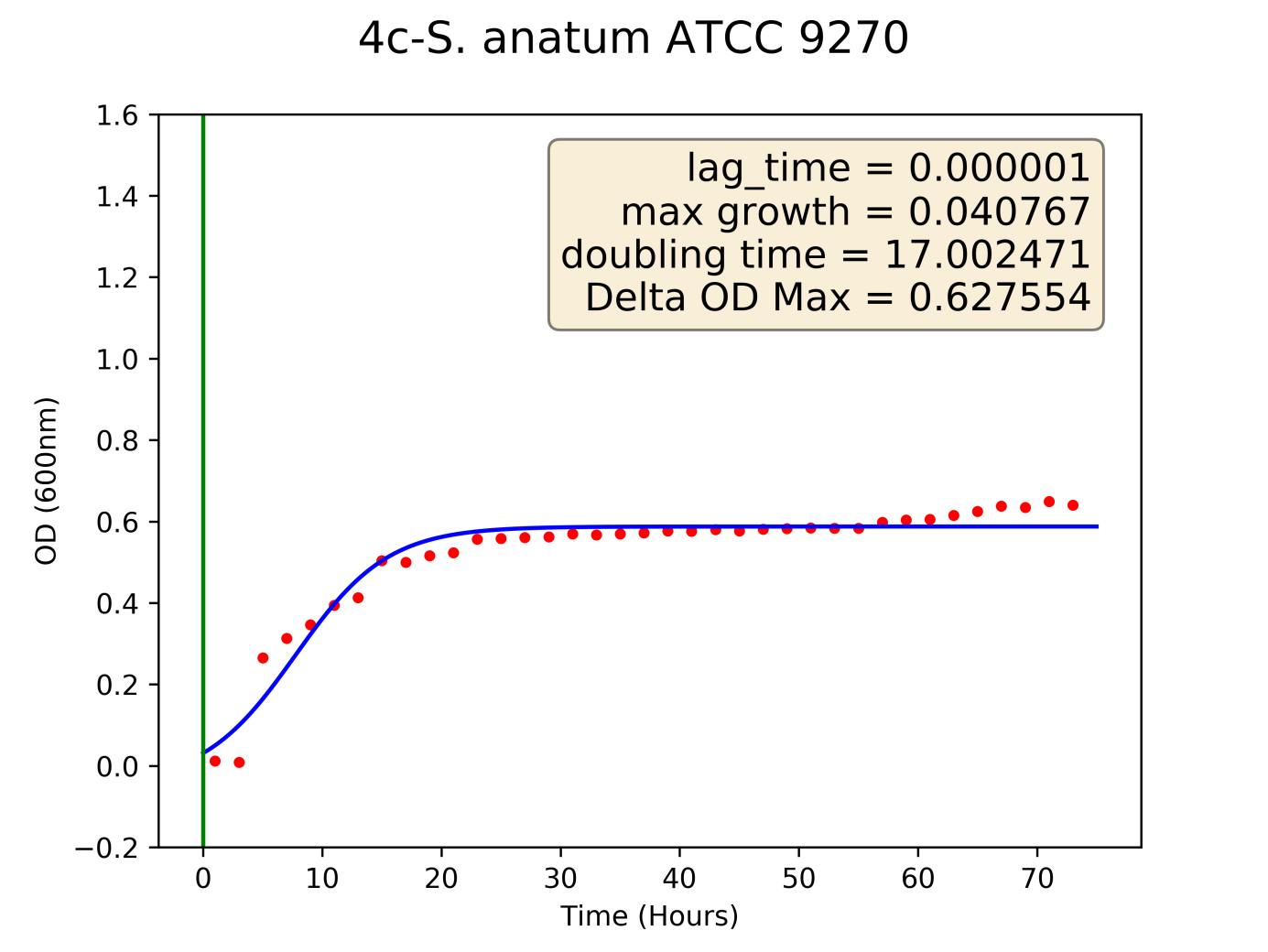


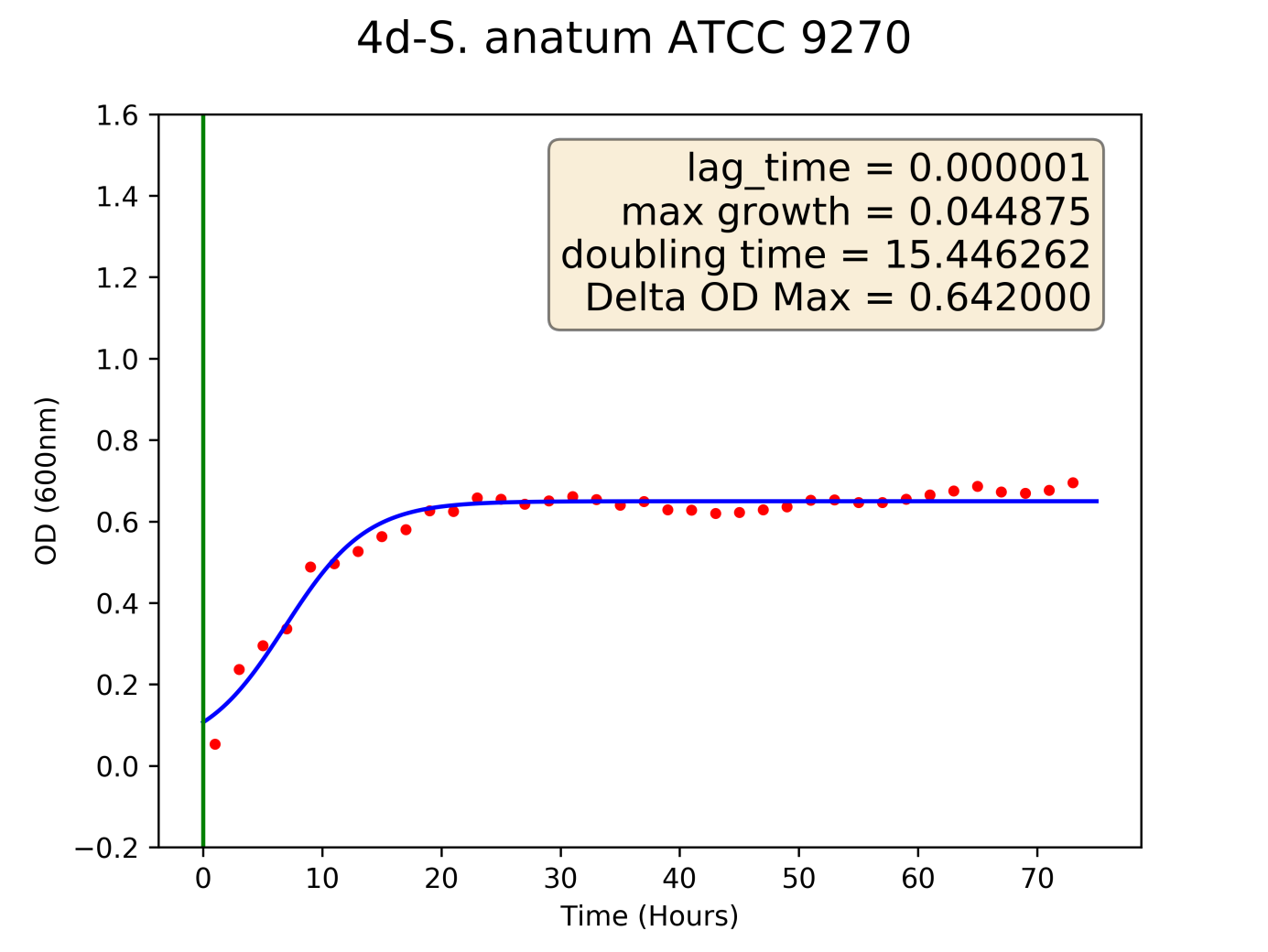


**Fig. S1.** Regression curves of selected data. *S. anatum* (1, 2, 3, 4 – types of TiO2; a, b, c, d – concentration of TiO2 : 60, 150, 300 i 600 mg/L)

Supplement: Supplementary file 1 — (ZIP 8.20 MB) [file 12011_2021_2843_MOESM1_ESM.zip › 2S4 (S. anatum)_ESM.docx]

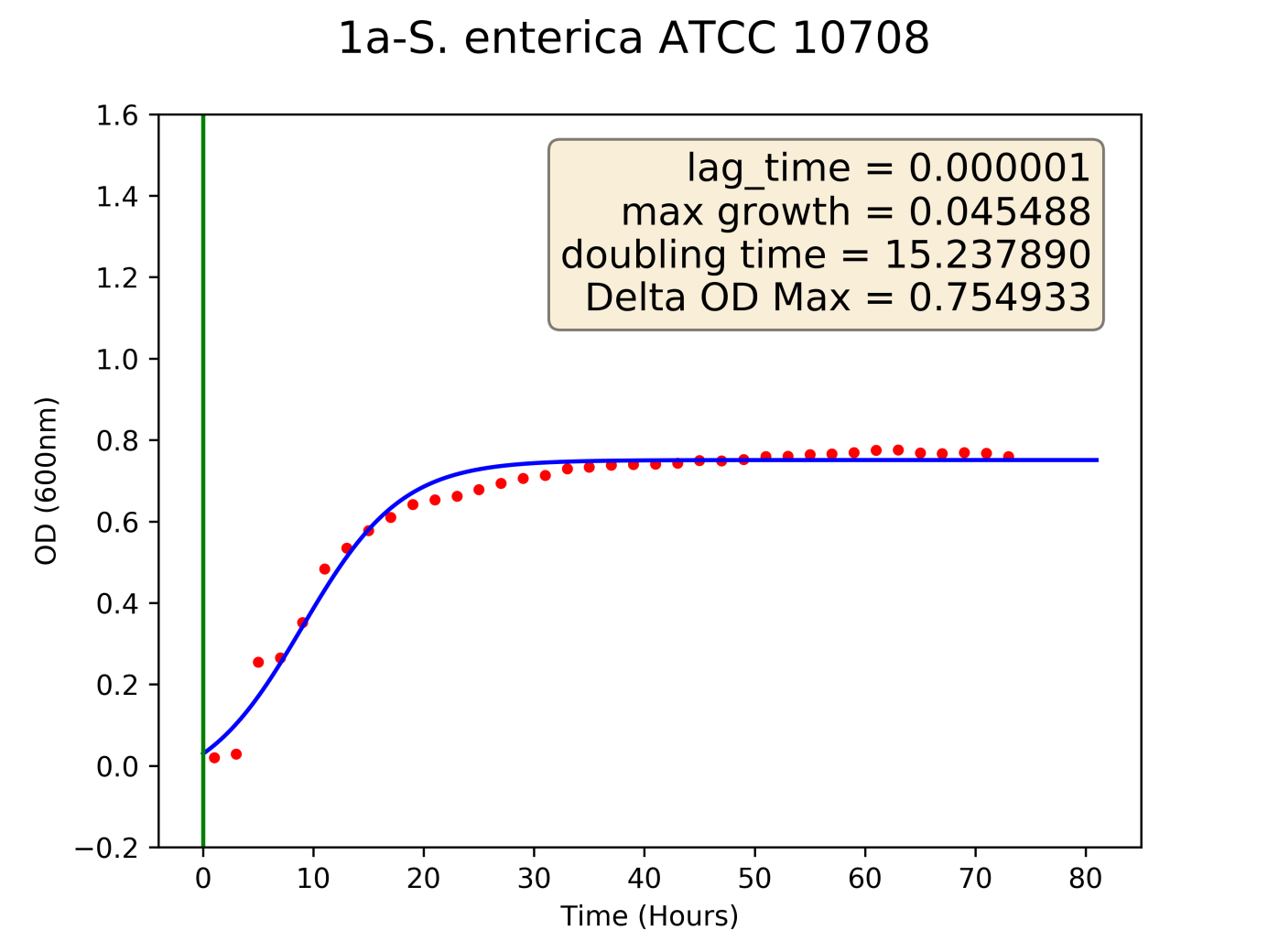


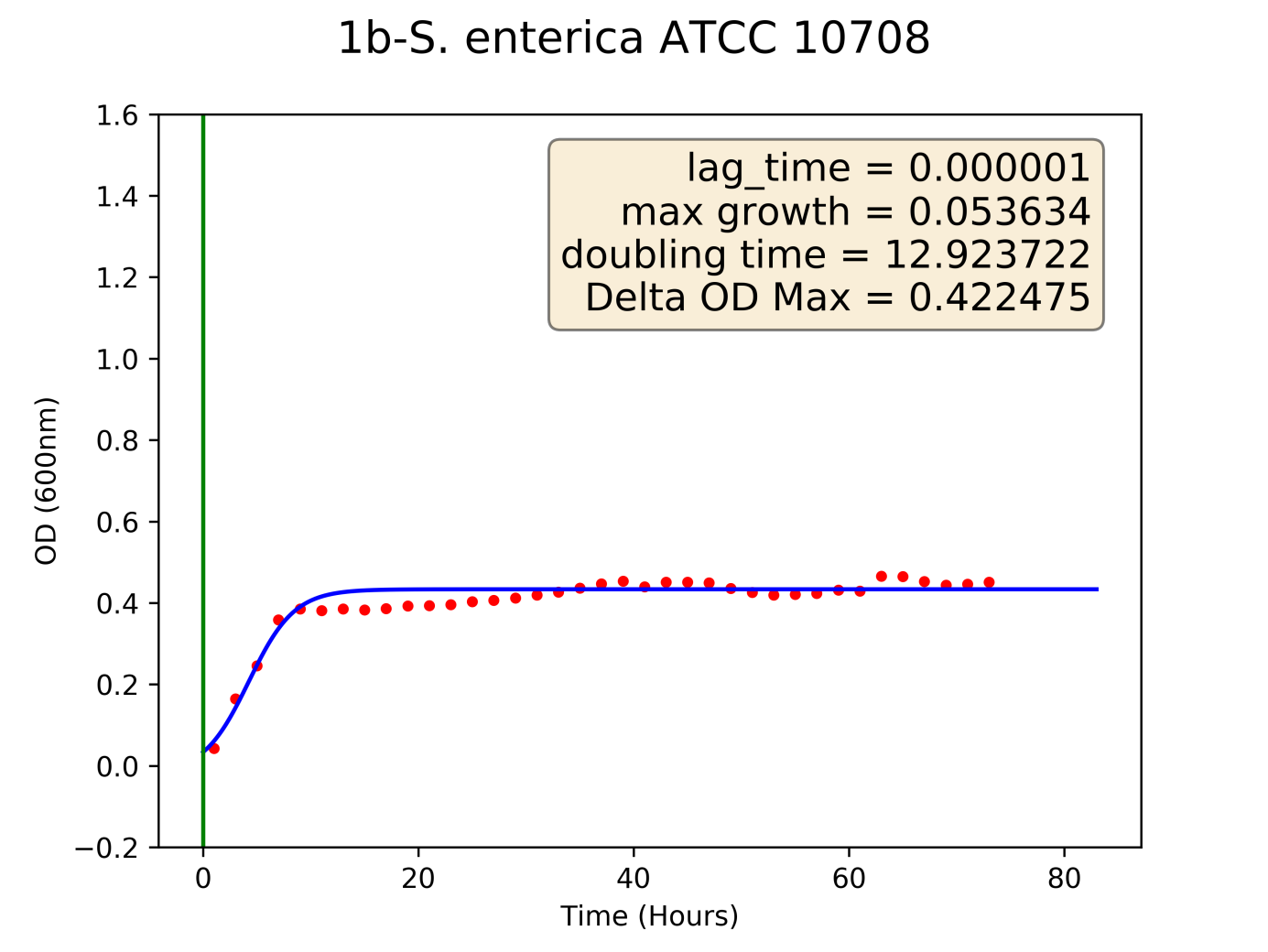


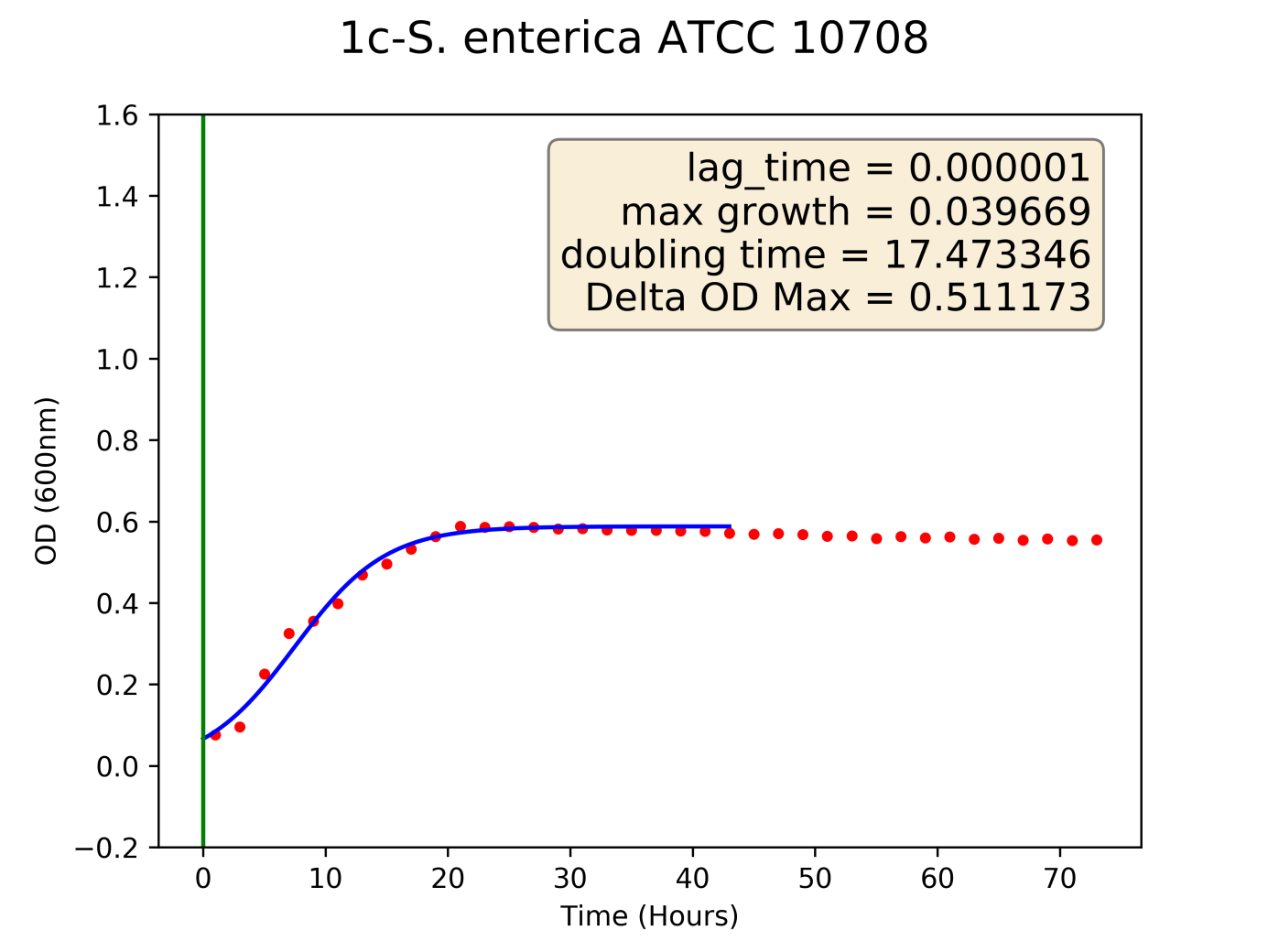


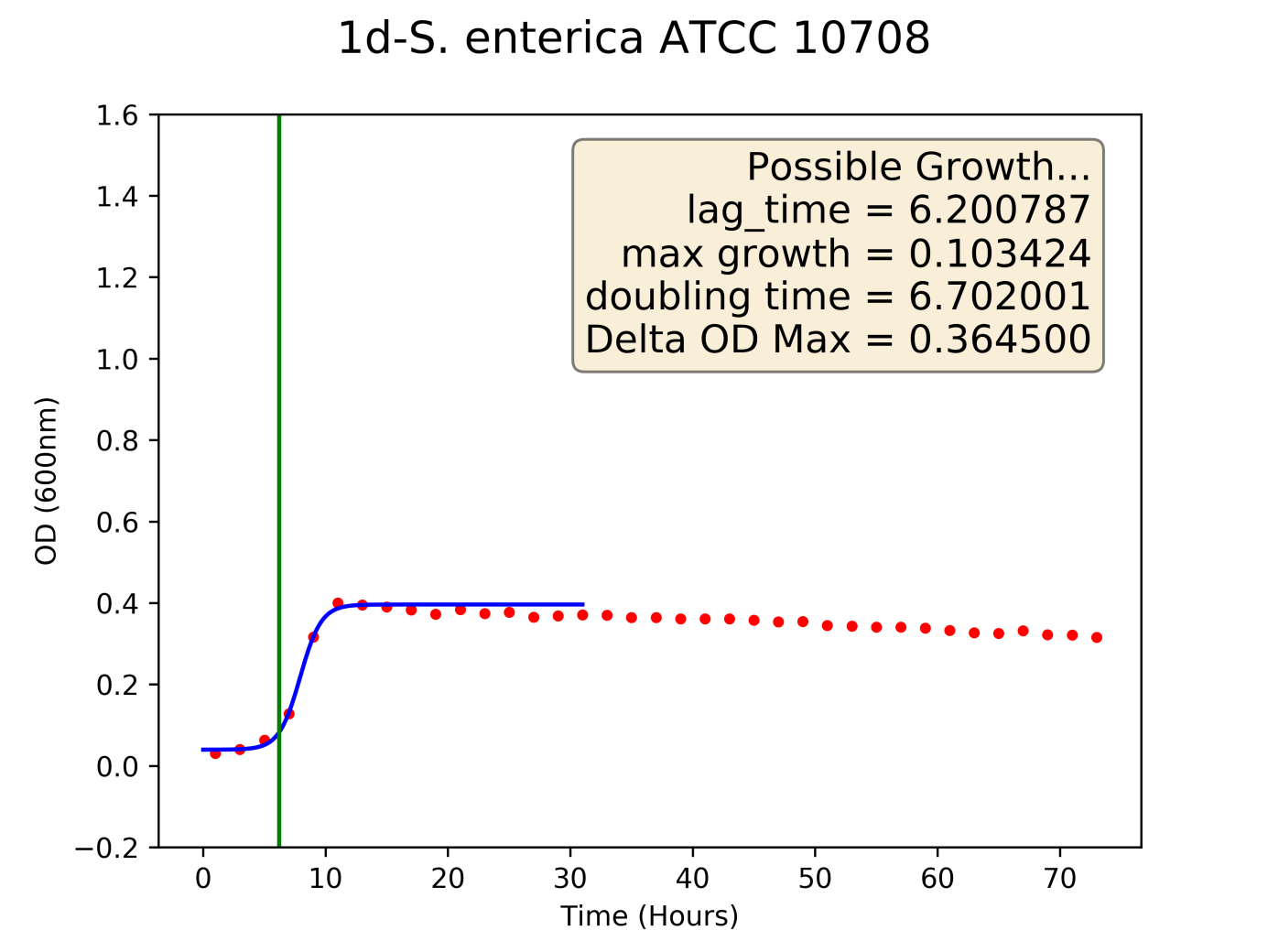


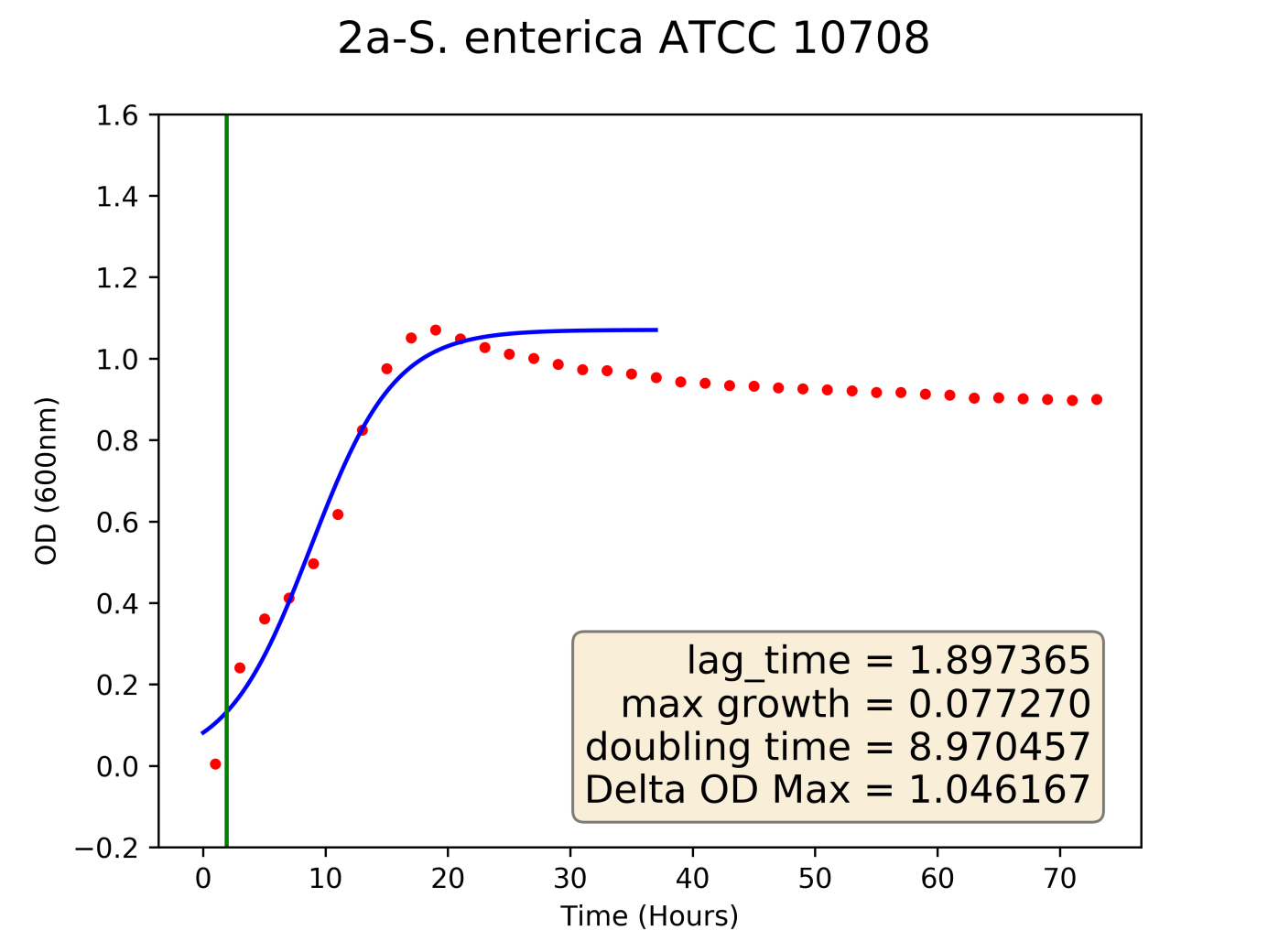


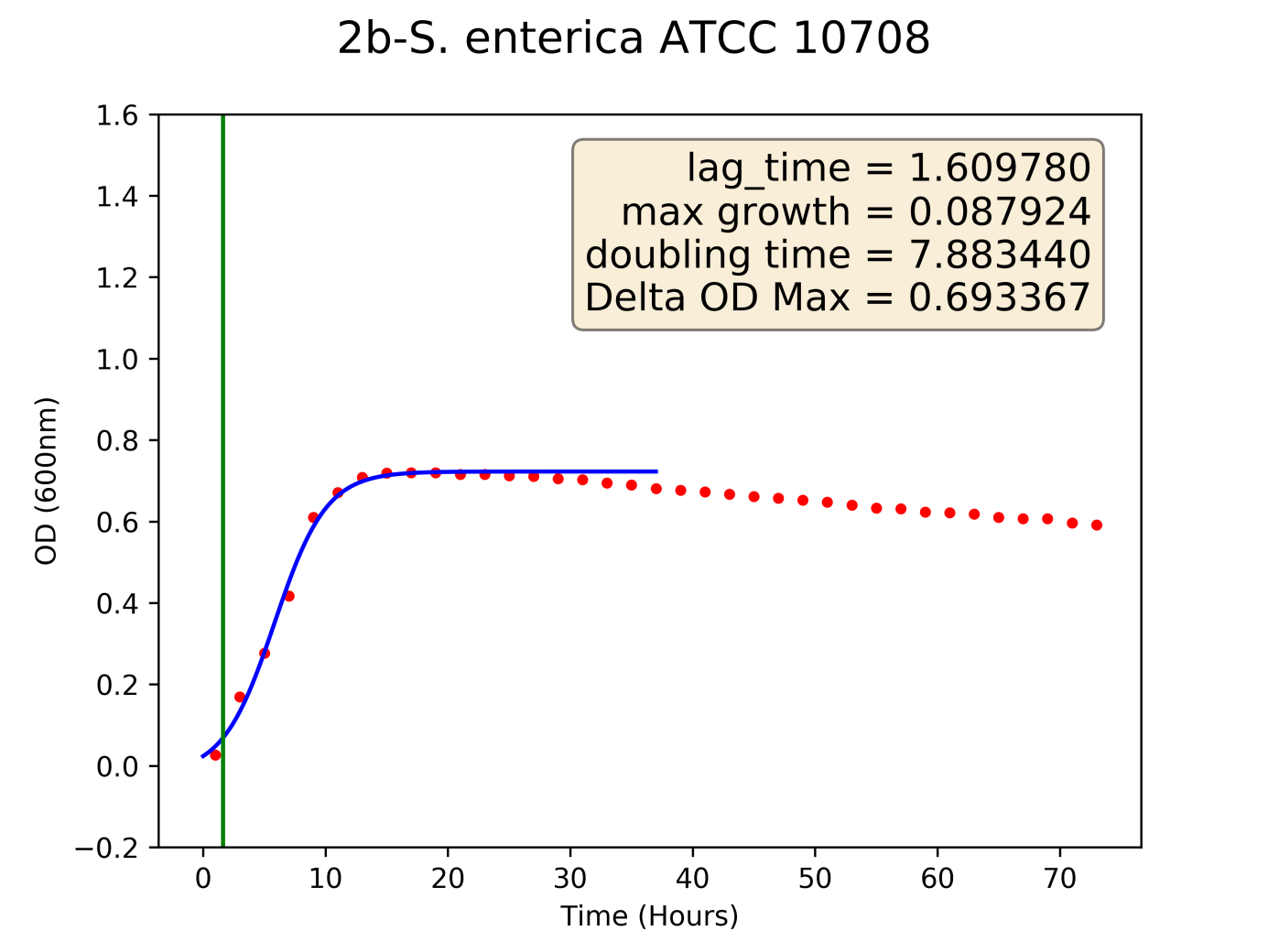


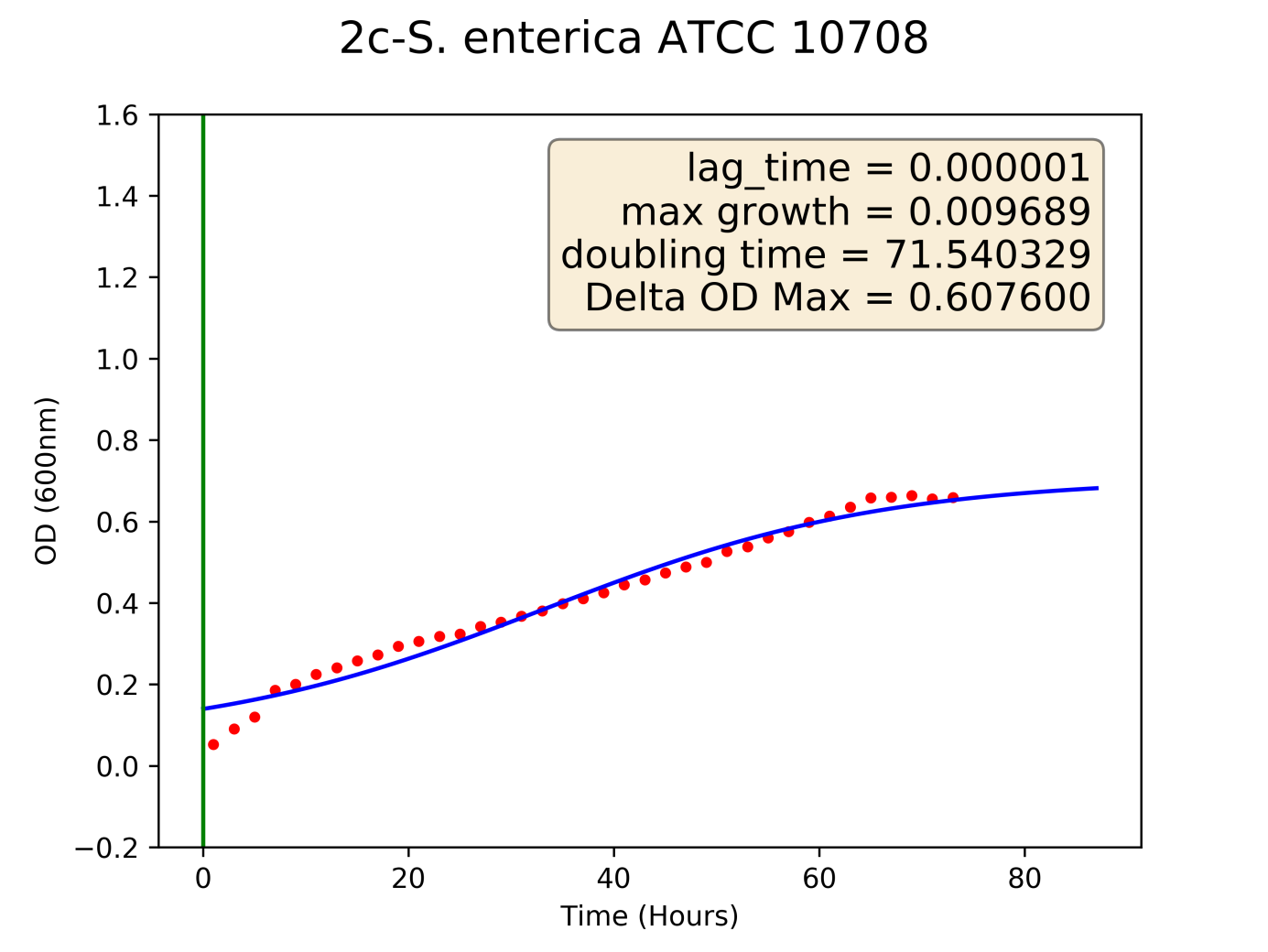


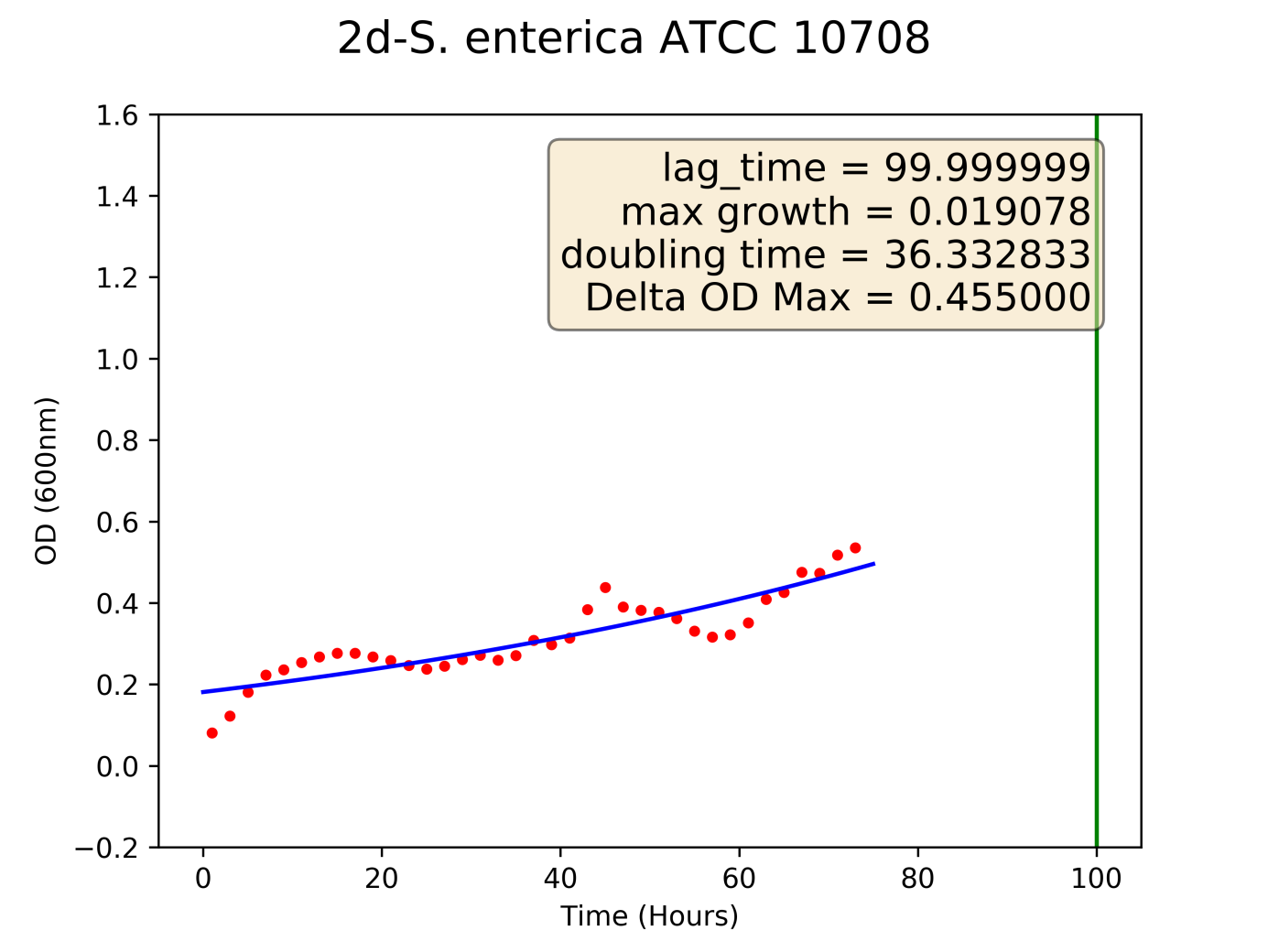


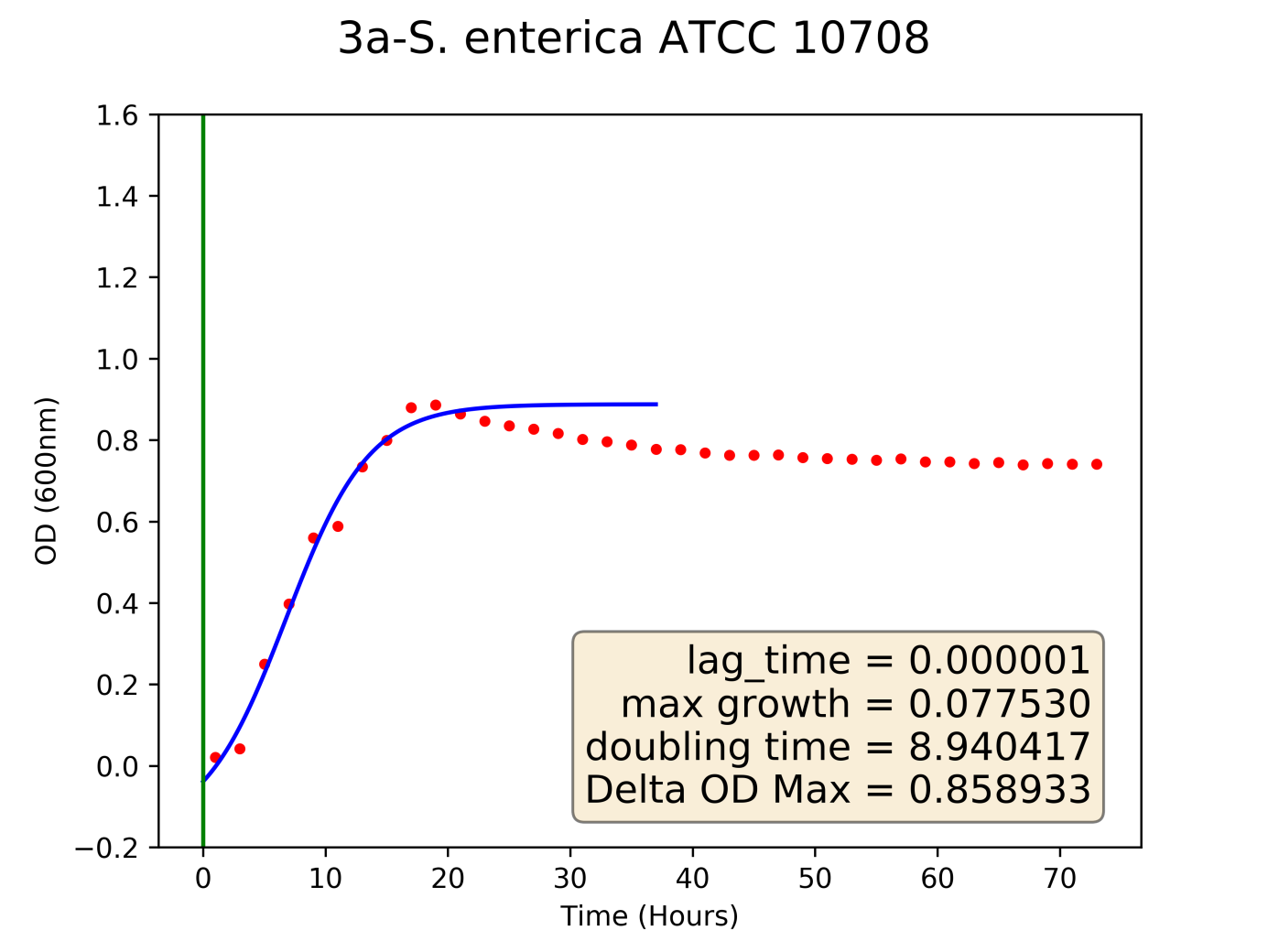


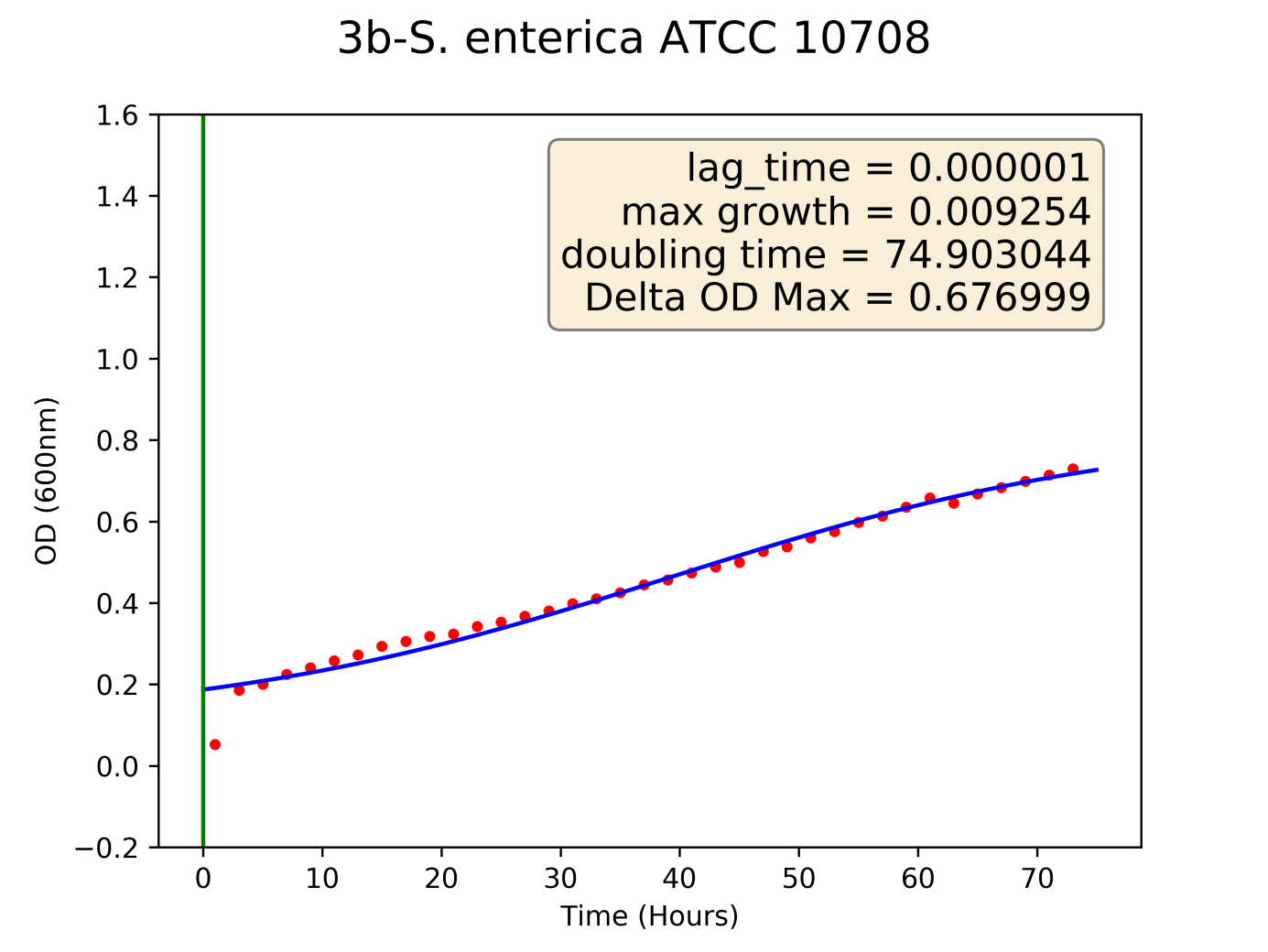


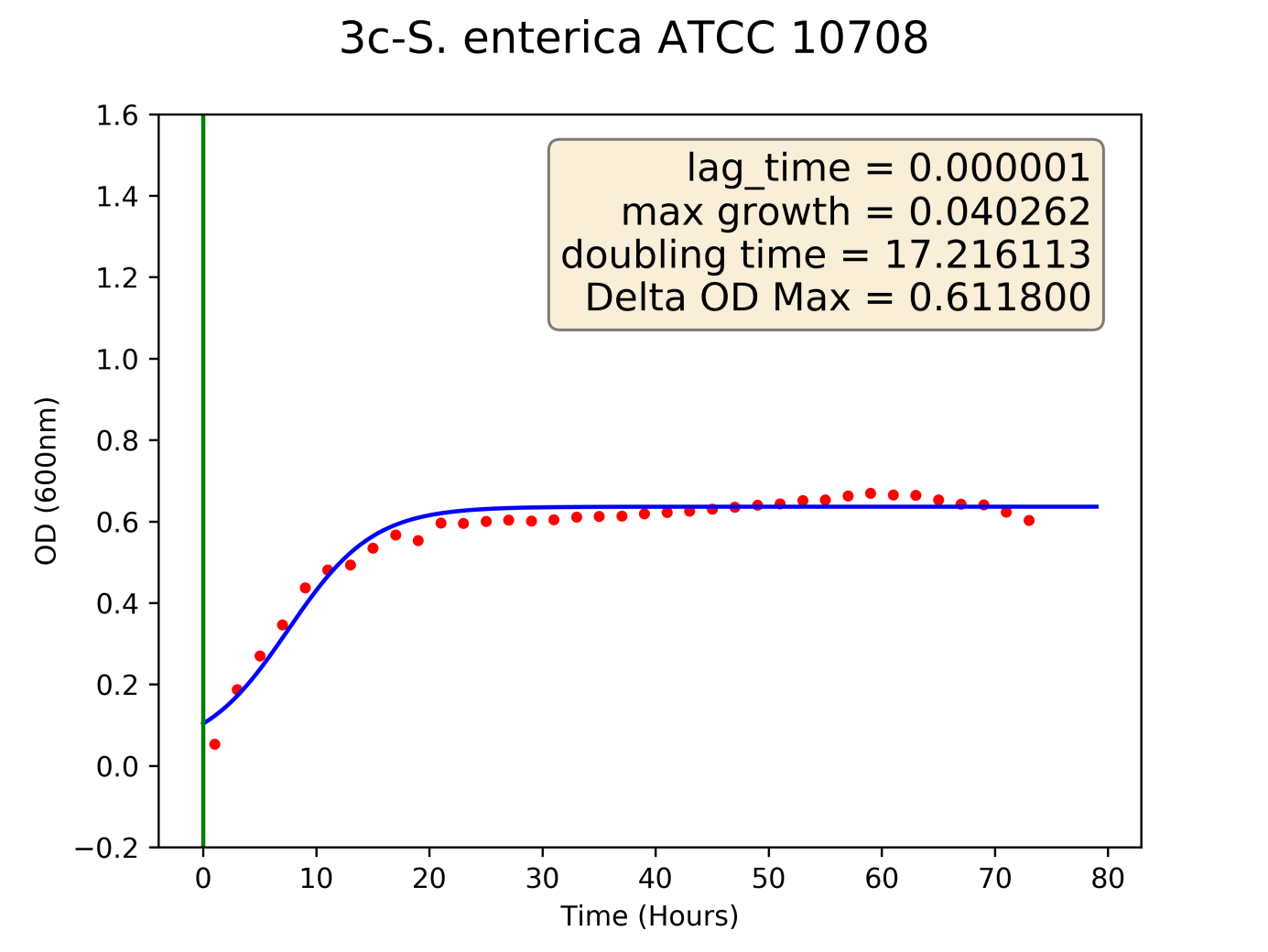


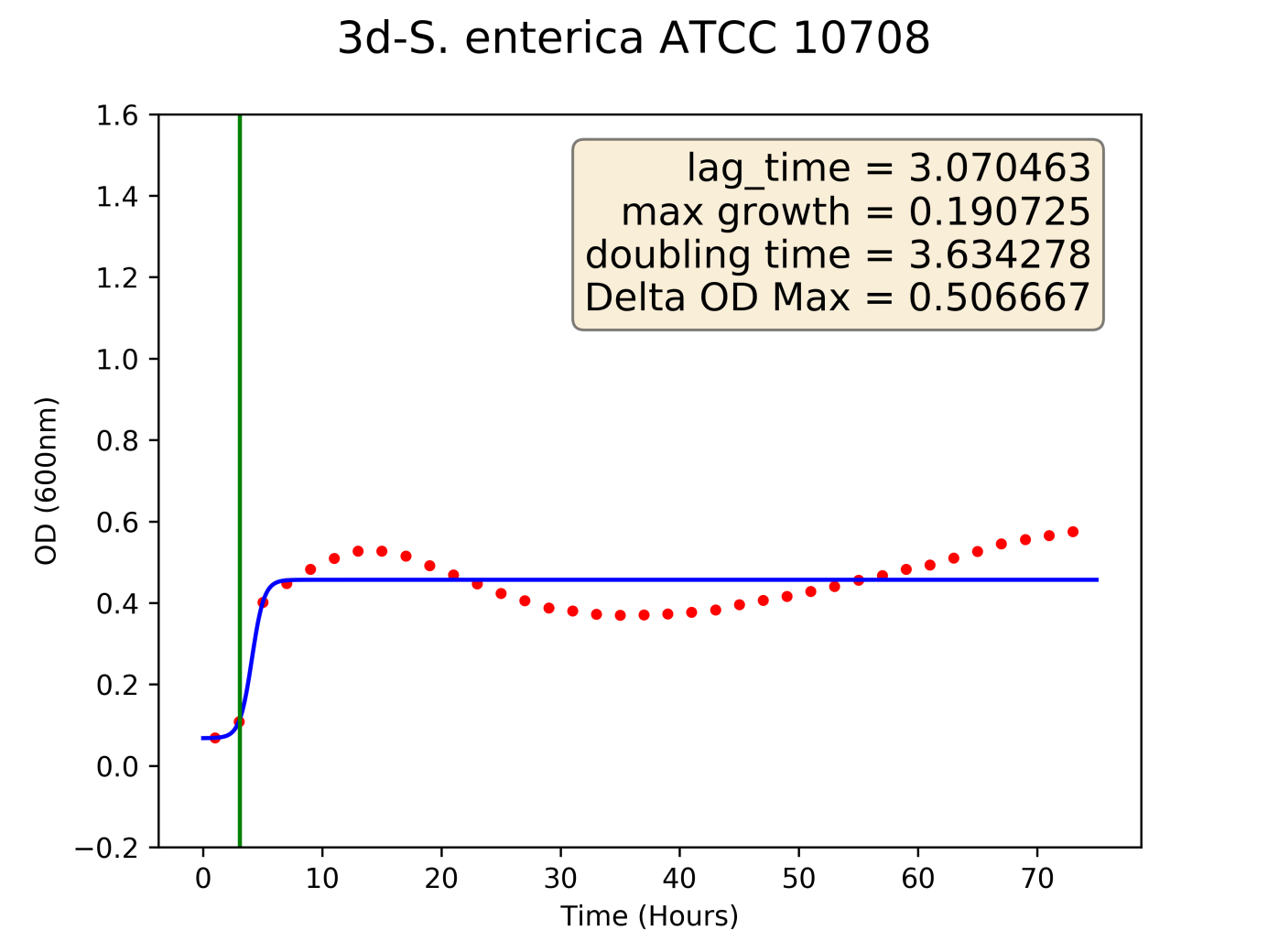


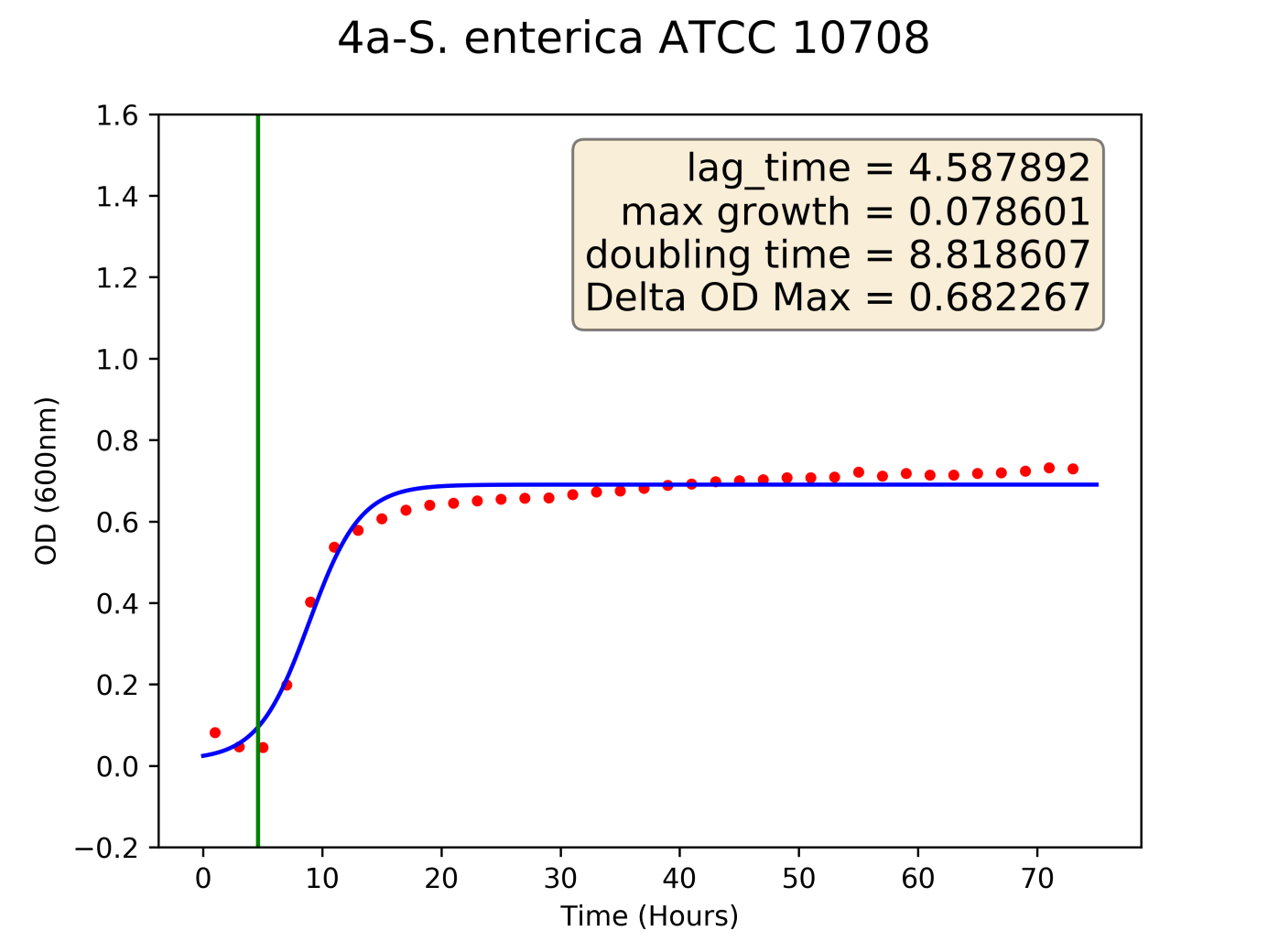


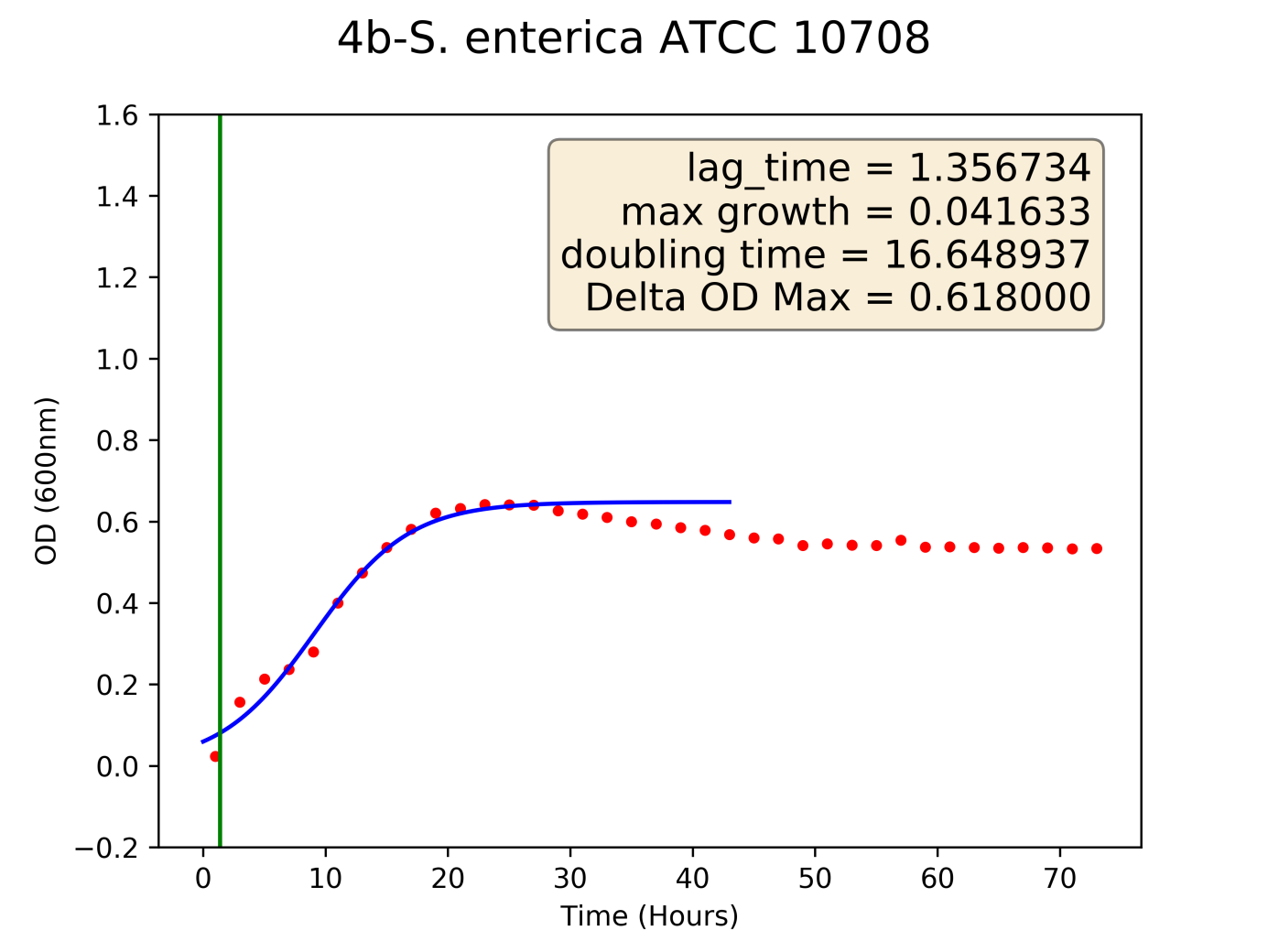


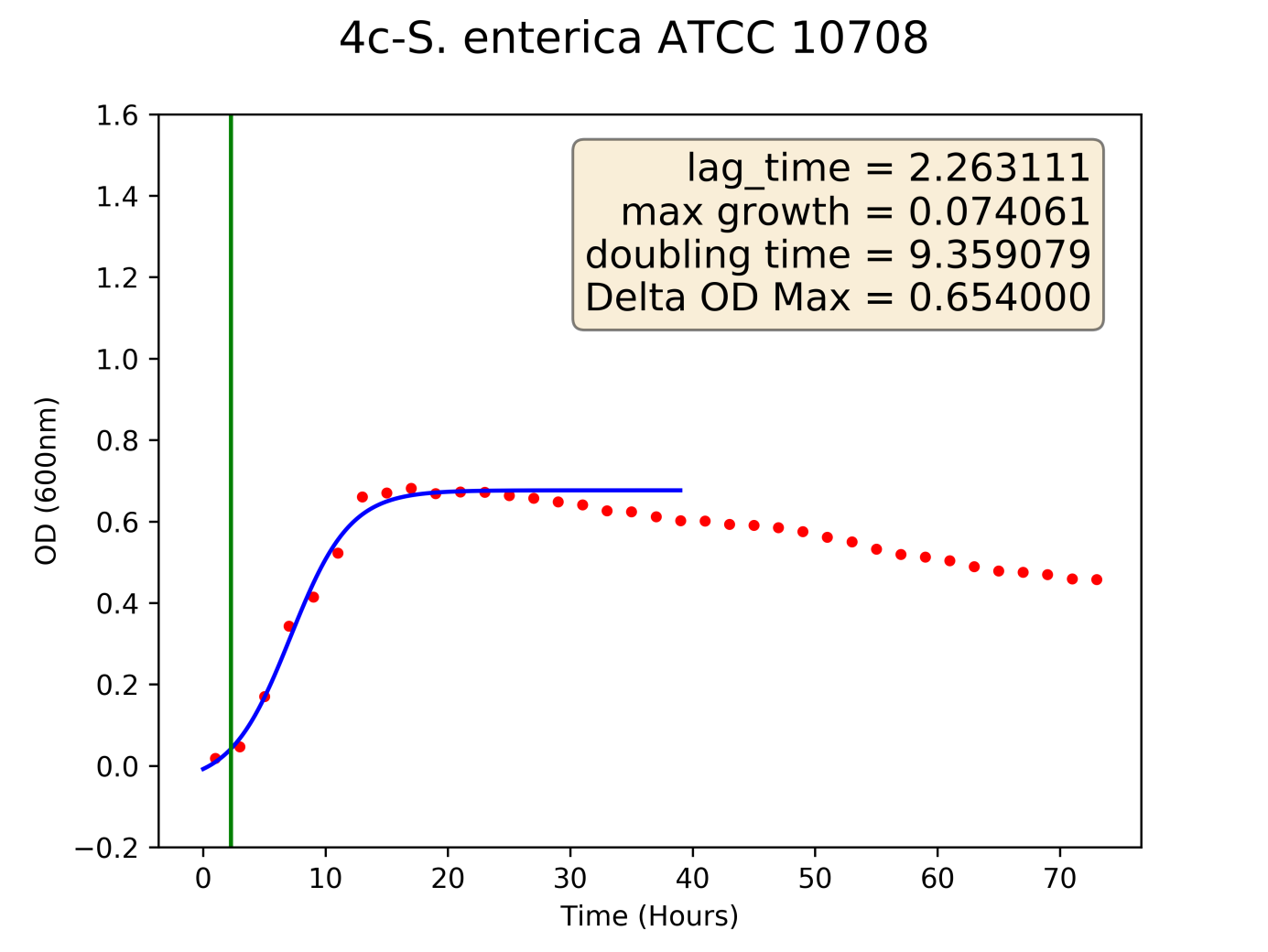


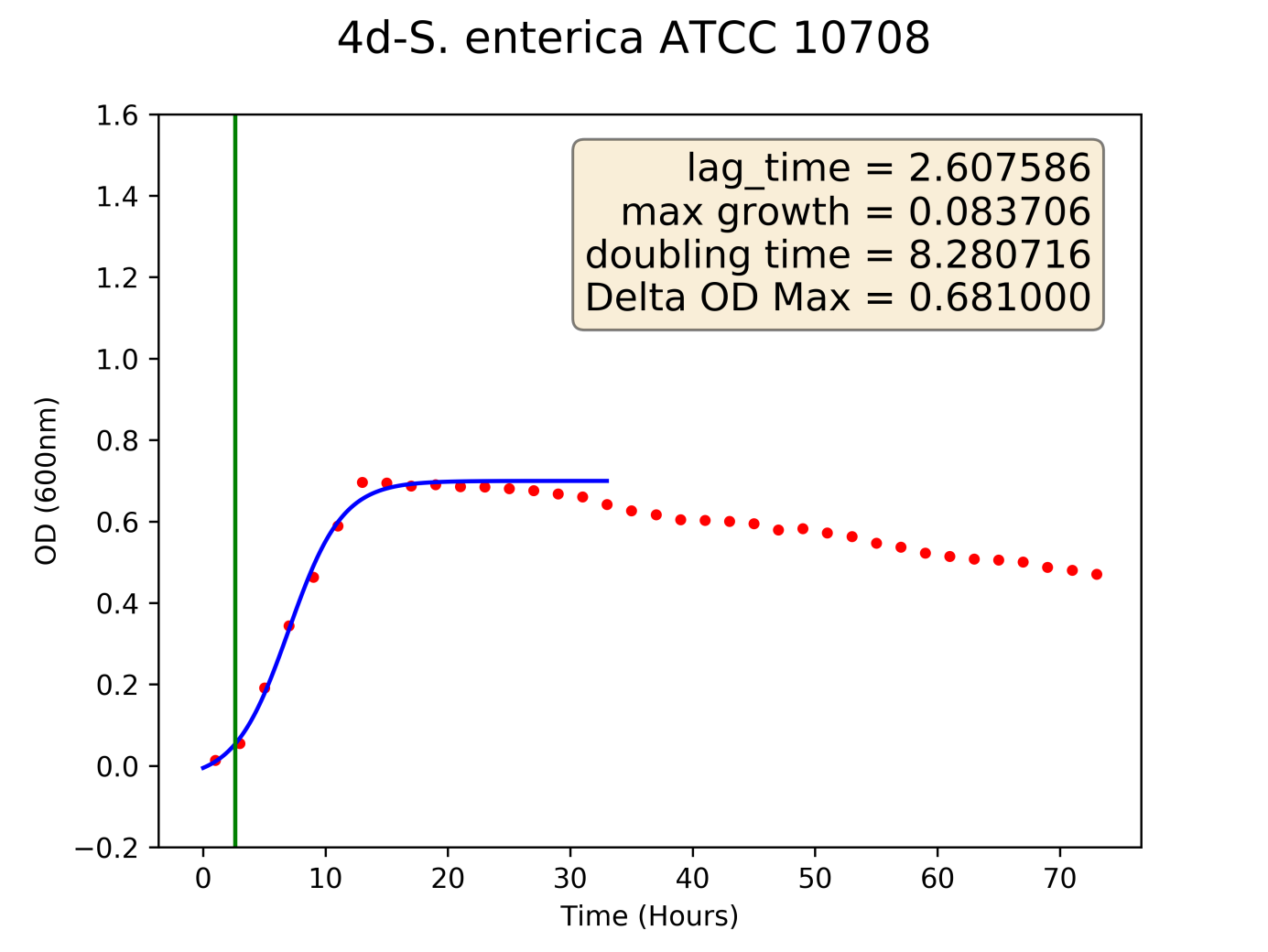


**Fig. S1.** Regression curves of selected data. *S. enterica* (1, 2, 3, 4 – types of TiO2; a, b, c, d – concentration of TiO2 : 60, 150, 300 i 600 mg/L)

Supplement: Supplementary file 1 — (ZIP 8.20 MB) [file 12011_2021_2843_MOESM1_ESM.zip › 2S5 (S. enterica)_ESM.docx]
