# Supplementary material for: Effect of TiO2 on Selected Pathogenic and Opportunistic Intestinal Bacteria
Source: Biol Trace Elem Res. 2021 Jul 23;200(5):2468–74. doi: 10.1007/s12011-021-02843-7 (PMC9023387; doi:10.1007/s12011-021-02843-7)
Supplement: Supplementary file 2 — (DOCX 438 KB) [file 12011_2021_2843_MOESM2_ESM.docx]

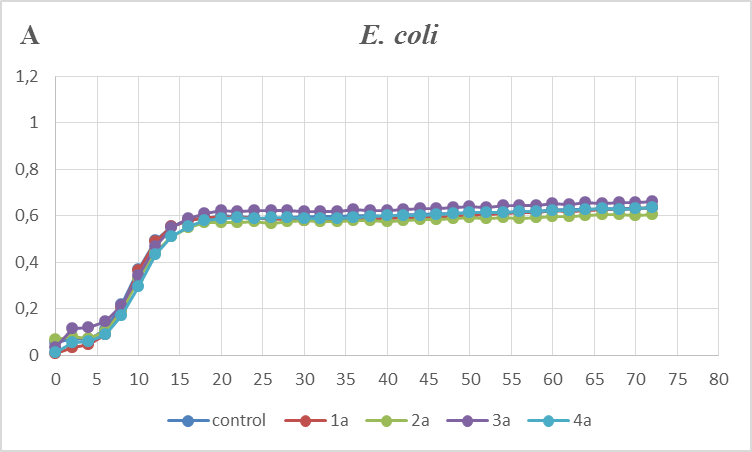


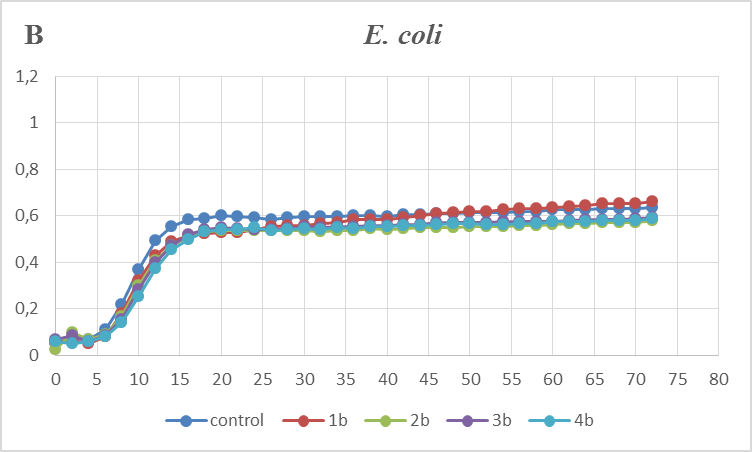


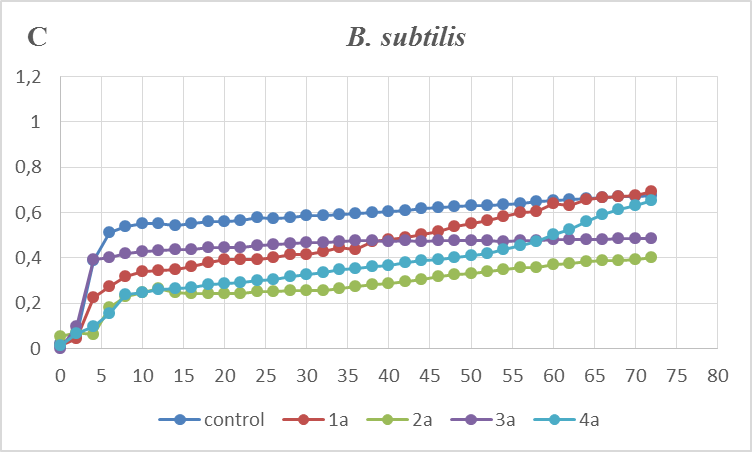


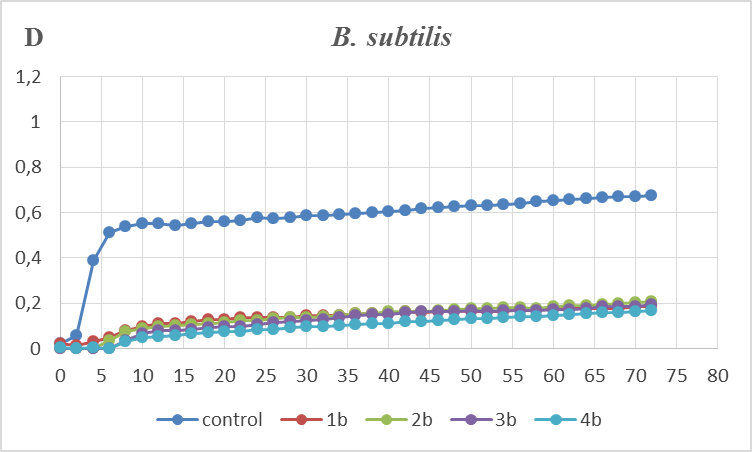


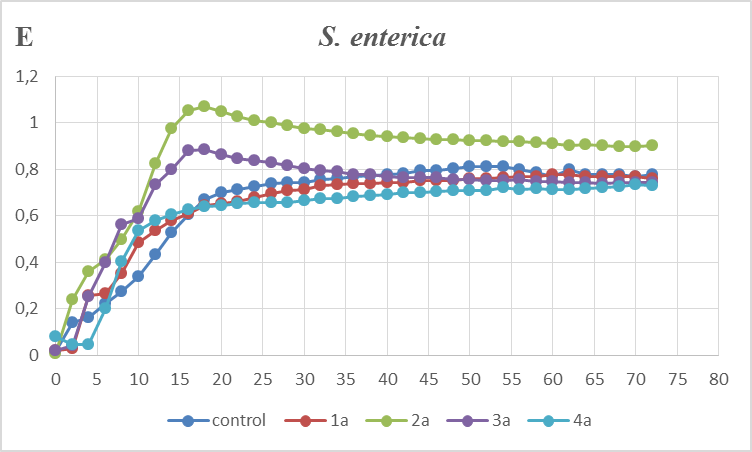


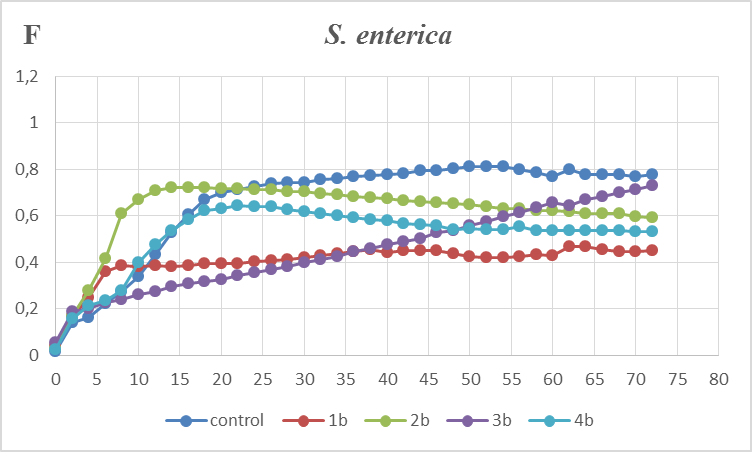


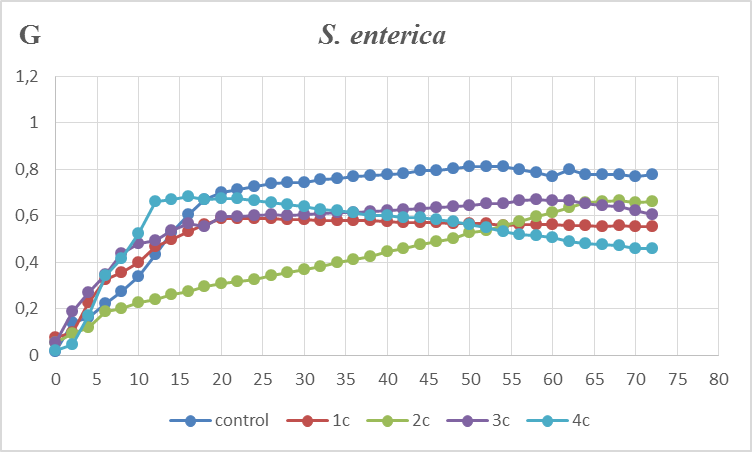


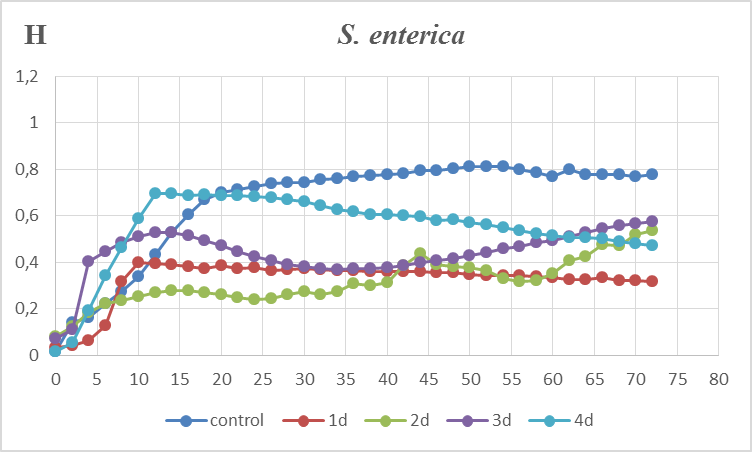


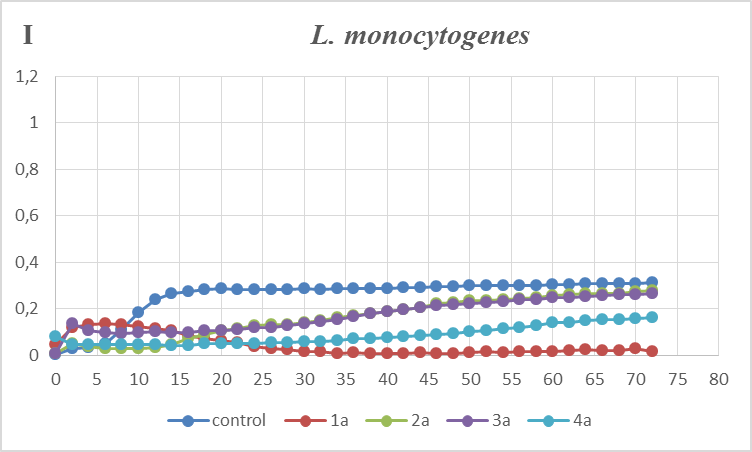


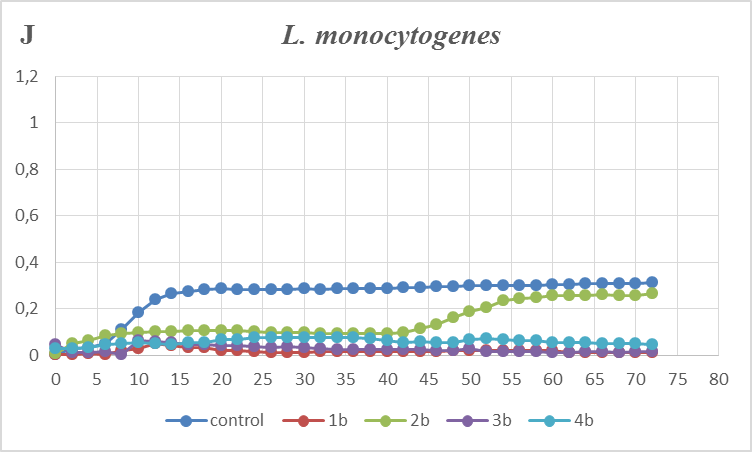


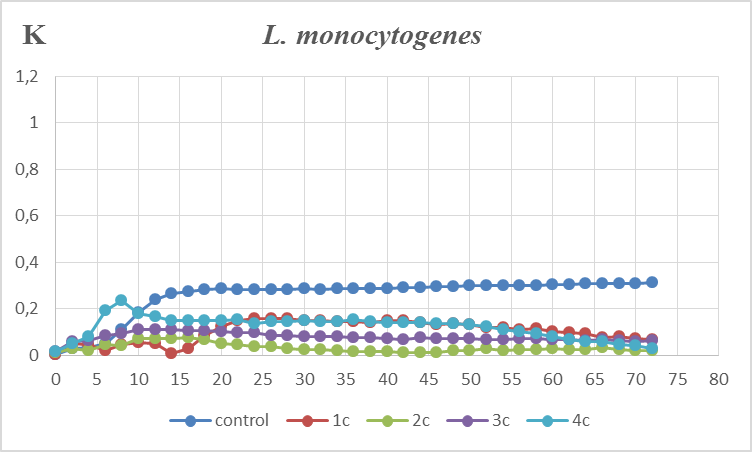


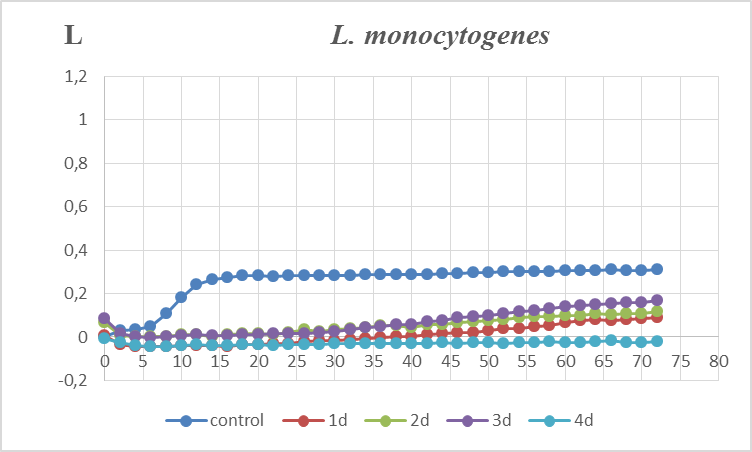


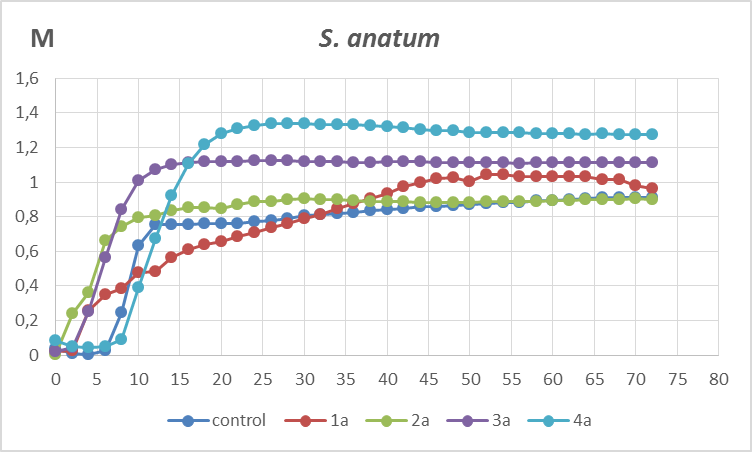


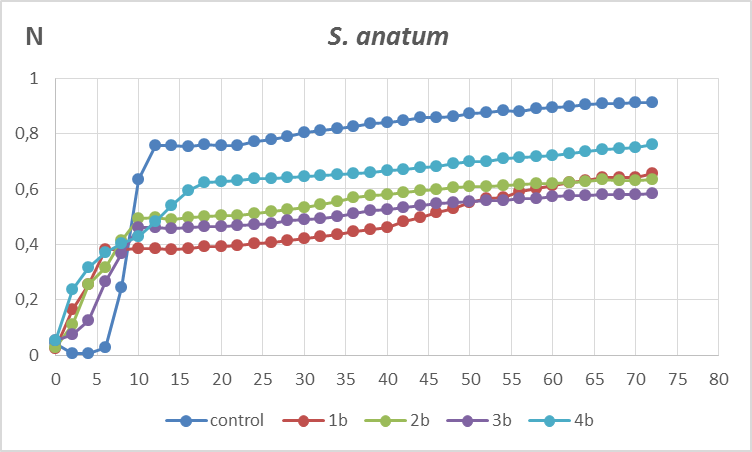


**Fig. 1S1** Growth of selected bacteria after application of four types of TiO_2_ at the concentration of 60 (a),150 (b),300 (c), 600 (d) mg/L; E171 (No. 1, 2, 3), TiO2 NPs (No. 4)
